# Proteinoids–Polyaniline Interaction with Stimulated Neurons on Living and Plastic Surfaces

**DOI:** 10.1021/acsomega.4c03546

**Published:** 2024-11-05

**Authors:** Panagiotis Mougkogiannis, Anna Nikolaidou, Andrew Adamatzky

**Affiliations:** Unconventional Computing Laboratory, UWE, Bristol, BS16 1QY, U.K.

## Abstract

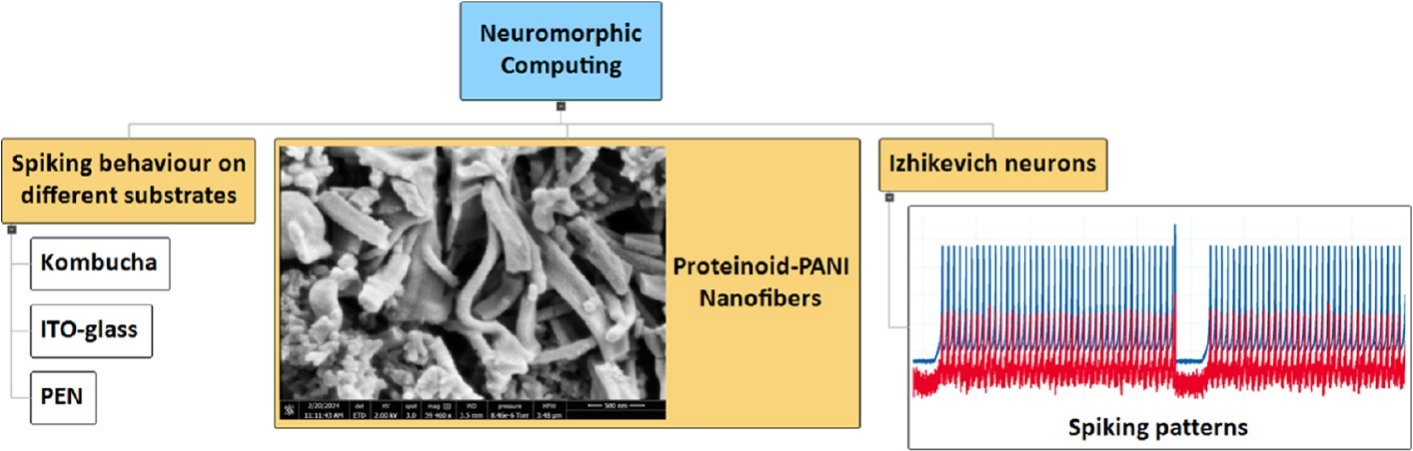

The integration of proteinoid-polyaniline (PANI) nanofibers with neuromorphic architectures shows potential for developing computer systems that are adaptable, energy-efficient, and have the capacity of tolerating faults. This work examines the capacity of proteinoid-PANI nanofibers to imitate different spiking patterns in stimulated Izhikevich neurons. The proteinoid-PANI nanofibers exhibit diverse spiking behaviors on different substrates, showcasing a broad range of control and programmability, as confirmed by experimental characterization and computational modeling. K-means clustering technique measures the extent and selectivity of the proteinoid-PANI spiking behavior in response to various stimuli and spiking patterns. The presence of strong positive correlations between membrane potential and time suggests that the system is capable of producing reliable and consistent electrical activity patterns. Proteinoid-PANI samples demonstrate enhanced stability and consistency in numerous spiking modes when compared to simulated input neurons. The results emphasize the capability of proteinoid-PANI nanofibers as a bioinspired substance for neuromorphic computing and open up possibilities for their incorporation into neuromorphic structures and bioinspired computer models.

## Introduction

Proteinoids–Polyaniline (PANI) composites are a highly promising group of materials that show great potential in a wide range of applications, especially in the areas of bioelectronics and brain interfaces. These composites include the distinct characteristics of proteinoids, which are artificially created proteins with clearly defined shapes and functions, and polyaniline, a conductive polymer known for its exceptional electrical and optical capabilities.^1,2^ The integration of proteinoids with polyaniline in a composite material has the potential to create advanced biomaterials that have improved biocompatibility, electrical conductivity, and functionality.^3,4^

Analyzing the interactions between Proteinoids–Polyaniline composites and simulated neurons is crucial for understanding the fundamental principles that drive neural interfaces and for developing efficient bioelectronic applications. Neural interfaces seek to develop a direct means of connection between the nervous system and electrical devices. This allows for the capturing, stimulating, and controlling of brain activity.^5,6^ It is essential to advance neural interface technology through the development of materials that can effectively interact with neurons and enable the transmission of electrical and chemical signals.

Conversely, polyaniline is a thoroughly researched conductive polymer that has attracted considerable interest because of its exceptional electrical conductivity, stability, and simple manufacture.^7^ Polyaniline nanostructures, including nanofibers and nanospheres, have been intensively studied for their potential uses in sensors, actuators, and energy storage devices.^8−10^ Studies have demonstrated that adding polyaniline to composite materials improves their electrical and mechanical characteristics, making them suitable for a range of bioelectronic applications.^11^ A substantial domain of experimental laboratory research on developing neuromorphic architectures with polyaniline has been established in refs (12−16).

Polyaniline’s chemical structure is made up of repeating units that are reduced (amine nitrogen atoms, −NH−) and oxidized (imine nitrogen atoms, =N−). Polyaniline’s oxidation state, leucoemeraldine (fully reduced), emeraldine (partially oxidized), and pernigraniline (fully oxidized), depends on the ratio of these units.^17^ When polyaniline is exposed to in an acidic environment, it undergoes deprotonation in its emeraldine form. This process will generate radical cations and a conductive salt called emeraldine salt. By conducting this chemical process, the electrical conductivity of polyaniline is improved, making it a highly attractive material for a wide range of applications.

**Figure 1:**
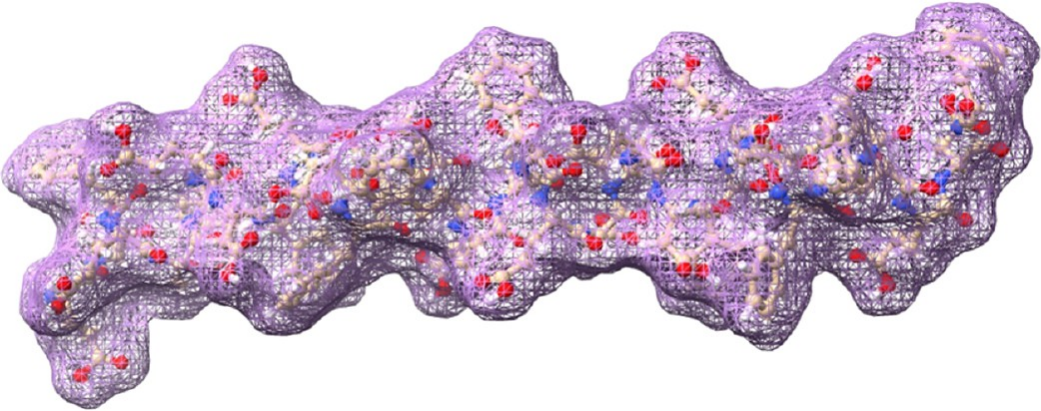
Proteinoid L–Glu:L–Asp:L–Phe, made up of 11 amino acid sequences, is shown here using a mesh visualization. With the mesh representation, you can get a clear view of the proteinoid’s three–dimensional structure, revealing the spatial arrangement of the amino acid sequences. The proteinoid is composed of repeating units of glutamic acid (Glu), aspartic acid (Asp), and phenylalanine (Phe). These amino acids are connected through peptide bonds that are formed during the thermal condensation reaction. Examining the mesh visualization provides a clear view of the network of connections between the amino acid residues, allowing us to understand the complex architecture of the proteinoid molecule. By examining the molecular representation, one can gain valuable insights into the proteinoid’s structural features. These features are responsible for its distinct properties and potential uses in a range of fields, including drug delivery, tissue engineering, and biosensing.

Proteinoids, or thermal proteins (Figure 1), are created by heating amino acids to their melting point, which triggers polymerization and forms polymeric chains. This process takes place at temperatures ranging from 160 to 200 °C, without the need for a solvent, initiator, or catalyst, and in an inert atmosphere. During this process, tri-functional amino acids such as glutamic acid, aspartic acid, or lysine undergo cyclization at high temperatures, serving as both solvents and initiators for the polymerization of other amino acids.^18,19^ This simple thermal condensation reaction allows for the creation of either acidic or basic proteinoids.

When these proteinoids are swollen in an aqueous solution at moderate temperatures (around 50 °C), they form structures called microspheres^19^ (Figure 1), which are usually hollow and filled with an aqueous solution. The growth of these microspheres can be programmed, with sizes ranging from 20 to 200 μm, by selecting specific amino acids and adjusting the thermal conditions.^19^ Proteinoid microspheres can maintain a steady–state membrane potential of 20 to 70 mV without any external stimulation, and some microspheres even exhibit a steady opposite polarization.^20^

When proteinoid microspheres are impaled with microelectrodes, they display electrical membrane potentials, oscillations, and action potentials, including action–potential–like spikes. This electrical activity consists of spontaneous bursts of electrical potential (flip–flops) and miniature potential activities during flopped phases.^21^ The amplitude of these spikes varies depending on the size of the microspheres and the presence of lecithin, with 20 μm microspheres having an amplitude of 20 mV and 200 μm microspheres with lecithin reaching amplitudes of 70 mV. In phospholipid–free microspheres, the amplitude of spiking is regular.^22^ Membrane, action, and oscillatory potentials recorded from microspheres composed of thermal protein, glycerol, and lecithin^21,22^ can be observed for several days.^23^ These microspheres remain stable^24^ in water at pH levels above 7 and continue to oscillate for weeks.^20^

Future uses of Proteinoids–Polyaniline composites in neural interfaces are extensive and promising. These composites can be used for neural recording, allowing them to detect and amplify the electrical impulses produced by neurons, thereby enabling the monitoring of neural activity.^5^ Proteinoids–Polyaniline composites have the potential to be used for brain stimulation. This involves delivering electrical stimuli to targeted areas of the nervous system in order to regulate neural activity and address neurological problems.^6^ Furthermore, these composite materials can function as biocompatible frameworks for the engineering of brain tissue, enhancing the development and specialization of neurons and aiding in the restoration of injured neural tissues.^25^

Simulated neural models are commonly used to investigate the interactions between Proteinoids–Polyaniline composites and neurons. The Hodgkin–Huxley model and the Izhikevich model are examples of simplified representations of neural behavior. They are used to study how neurons respond to different stimuli.^26^ By replicating the electrical behavior of neurons and their interactions with the Proteinoids–Polyaniline composite, we can obtain essential information on the compatibility with living organisms, transmission of signals, and overall functionality of the composite material under controlled conditions. Simulated neuronal models provide a cost–effective and efficient method for understanding the intricate dynamics of neural interfaces prior to conducting in vitro or in vivo experiments. The Izhikevich model is a simple spiking neuron model that can reproduce the rich firing patterns of real biological neurons. The model used for simulating neuron behavior is a two–dimensional system of ordinary differential equations with a reset condition, as described by eqs 1, 2, and 3:
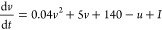
1
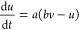
2The reset condition is
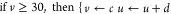
3Here, *v* represents the membrane potential of the neuron, *u* represents a membrane recovery variable accounting for the activation of K^+^ ionic currents and inactivation of Na^+^ ionic currents, and *a*, *b*, *c*, and *d* are dimensionless parameters. The variable *I* represents synaptic currents or injected dc-currents. The model can exhibit various intrinsic firing patterns, including those of regular spiking (RS), intrinsically bursting (IB), and chattering (CH) neurons, depending on the values of the parameters *a*, *b*, *c*, and *d*. It is computationally efficient yet biologically plausible, making it suitable for large-scale simulations of spiking neural networks.^26,27^ The parameter *a* controls the rate of recovery of *u*, and *b* determines the sensitivity of recovery to subthreshold fluctuations of the membrane potential. The parameters *c* and *d* define the after–spike reset values for *v* and *u*, respectively. By choosing suitable combinations of these parameters, the model can display different firing patterns and responses that are observable in biological neurons. The membrane potential *v* is determined by eq 1, which consists of a quadratic term, a linear term, and the effects of the recovery variable *u* and external input *I*. The recovery variable *u* follows the dynamics described by eq 2, which are influenced by the membrane potential *v* and the parameters *a* and *b*. The reset condition, as defined by eq 3, is activated when the membrane potential *v* hits or surpasses a threshold value of 30. Once the threshold is reached, the membrane potential *v* is reset to the value *c*, and the recovery variable *u* is increased by *d*. This reset process replicates the spike production and following hyperpolarization observed in real neurons. By integrating this model into the simulation framework, we can analyze the dynamics and functioning of individual neurons and investigate the impact of various parameter combinations on their firing patterns and sensitivity to external stimuli. Table 1 showcases the Izhikevich model’s capacity to capture diverse firing patterns. The table contains 20 distinct neuronal types, each defined by a unique set of parameters *a*, *b*, *c*, *d*, and *I*. These parameters govern the dynamics of the membrane potential *v* and the recovery variable *u*, allowing the model to create a variety of firing patterns seen in biological neurons.

**Table 1:** Parameter Combinations for the Izhikevich Model to Reproduce Various Neuronal Firing Patterns^a^

neuron type	*a*	*b*	*c*	*d*	*I*
Regular Spiking Neurons					
tonic spiking	0.02	0.2	–65	6	14
phasic spiking	0.02	0.25	–65	6	0.5
spike latency	0.02	0.2	–65	6	7
Bursting Neurons					
tonic bursting	0.02	0.2	–50	2	15
phasic bursting	0.02	0.25	–55	0.05	0.6
mixed mode	0.02	0.2	–55	4	10
Adapting Neurons					
spike frequency adaptation	0.01	0.2	–65	8	30
accommodation	0.02	1	–55	4	0
Resonant Neurons					
class 1	0.02	–0.1	–55	6	0
class 2	0.2	0.26	–65	0	0
resonator	0.1	0.26	–60	–1	0
Oscillatory Neurons					
subthreshold oscillations	0.05	0.26	–60	0	0
integrator	0.02	–0.1	–55	6	0
Rebound Neurons					
rebound spike	0.03	0.25	–60	4	0
rebound burst	0.03	0.25	–52	0	0
Other Specialized Types					
threshold variability	0.03	0.25	–60	4	0
bistability	1	1.5	–60	0	–65
DAP	1	0.2	–60	–21	0
inhibition-induced spiking	–0.02	–1	–60	8	80
inhibition-induced bursting	–0.026	–1	–45	0	80

a The table lists 20 different types of neurons and their corresponding parameter values for a, b, c, d, and I. These parameter sets showcase the versatility of the Izhikevich model in capturing a wide range of biologically observed neuronal behaviors.^28^

Proteinoids-Polyaniline composites are promising materials for neuromorphic computing due to their ability to mimic real neurons. These neurons can be used in spiking neural networks, reservoir computing, neuromorphic sensors, and neuromorphic robots to enhance computational capabilities, efficiency, and robustness. Their biocompatibility and reliable electrical activity make them suitable for brain-machine interfaces. We investigate the interaction of proteinoid-polyaniline nanofibers and simulated neurons on Kombucha, ITO-glass, and PEN substrates to assess their potential for neuromorphic computing, with each substrate offering unique advantages and limitations. We investigate the capacity of proteinoid–PANI nanofibers to replicate various spiking patterns, including thalamocortical, chattering, phasic, triggered, integrator, tonic, mixed mode, and accommodation spiking, observed in biological neurons. This work aims to examine the interactions between Proteinoids–Polyaniline composites and simulated neurons, with a specific focus on the electrical characteristics, biocompatibility, and functional performance of the composite material. Our objective is to analyze how simulated neurons react to Proteinoids–Polyaniline composites in order to understand the neural interface mechanisms and determine the crucial aspects that determine the composite material’s performance in bioelectronic applications.

## Methods and Materials

### Materials

Aniline (*d* = 1.022 g/mol, *M* = 93.13 g/mol, CAS-No: 62-53-3, ACS reagent >99.5%), *p-*Toluenesulfonic acid monohydrate (CAS-No 6192-52-5, ACS reagent >98.5%), and ammonium persulfate (*d* = 1.980 g/cm^3^, *M* = 228.20 g/mol, CAS: 7727-54-0, ACS reagent >98.0%) were obtained from Sigma-Aldrich. Hydrochloric acid (1 mol/L, 1 N) was procured from Merck KGaA. All chemicals were used as received without additional purification. l -Phenylalanine, l-Aspartic acid, and l-Glutamic acid, with purity levels surpassing 98%, were acquired from Sigma-Aldrich and employed as precursors for proteinoid synthesis. 1-Methyl-2-pyrrolidinone, having a purity exceeding 98%, was also purchased from Sigma-Aldrich. The PEN plastic was supplied by Merck under the catalog number GF80046851. This material is a film with a thickness of 0.125 mm and a length of 0.5 m, in its natural color. The ITO glass used was also sourced from Merck and is described as an Indium Tin Oxide coated glass slide in a square shape, featuring a surface resistivity of 8–12 Ω/sq.

### Synthesis of PANI Nanotubes–Nanospheres

The synthesis of PANI nanotubes–nanospheres was achieved through the chemical oxidative polymerization of aniline, employing ammonium persulfate as the oxidizing agent and *p*-Toluenesulfonic acid monohydrate as the dopant. In a typical synthesis, 5 mL of aniline was mixed with ammonium persulfate and *p*-Toluenesulfonic acid monohydrate in a molar ratio of 1:1:0.5. The mixture was stirred at room temperature for 1 h to ensure complete dissolution and homogenization. Following the stirring process, the mixture was kept at −8 °C for 3 days to enable a slow polymerization reaction, promoting the formation of nanotubes and nanospheres. The low temperature helps to regulate the growth rate and morphology of the PANI nanostructures. After polymerization, the resulting dark green precipitate was collected by filtration and washed several times with deionized water and ethanol to eliminate any unreacted monomers and impurities. The PANI nanotubes–nanospheres were then dried in a vacuum oven at 30 °C for 24 h.^29−31^

### Synthesis of Proteinoid Microspheres

Proteinoid microspheres were prepared following the thermal polycondensation method described by Mougkogiannis et al.^32^ The synthesis procedure involved the following steps:

1. A mixture of 5 g of l-phenylalanine, l-aspartic acid, and l-glutamic acid was prepared.
2. The amino acid mixture was placed in a 100 mL reaction vessel and heated at 180 °C under reflux.
3. The thermal polycondensation reaction was carried out for 180 min.
4. The synthesized proteinoids were then dispersed in an aqueous solution and stirred for 3 hours at 80 °C.
5. The proteinoid solution was lyophilized (freeze–dried) to remove any residual moisture and obtain a dry powder.
6. The lyophilized proteinoids were stored at room temperature for further characterization and use.

### Synthesis of PANI–Proteinoid Suspension

To prepare the PANI–proteinoid suspension, the synthesized PANI was first dissolved in 1-Methyl-2-pyrrolidinone (NMP) to create a 1 mg/L solution. NMP was chosen as the solvent due to its ability to effectively dissolve PANI and its compatibility with the proteinoid solution. In a separate container, the lyophilized proteinoids were dissolved in deionized water to form a proteinoid–water solution. The concentration of the proteinoid solution was adjusted based on the desired ratio of PANI to proteinoids in the final suspension. Next, the PANI–NMP solution was gradually added to the proteinoid–water solution under continuous stirring. The mixing process was carried out at room temperature to ensure a homogeneous distribution of PANI within the proteinoid solution.

### Preparation of kombucha mat

A kombucha mat, originally sourced from Freshly Fermented Ltd. (Lee–on–the–Solent, PO13 9FU, U.K.), was used to produce kombucha biofilms. The infusion was prepared by boiling 5 L of tap water in a plastic container and then adding 500 g of white granulated sugar (Tate & Lyle, U.K.) and 10 tea bags (Taylors Yorkshire Teabags 125 g, U.K.). The solution was allowed to cool down to room temperature before placing the kombucha mat into the container. The container was then stored in a dark environment at a temperature range of 20–23 °C.

### Characterization Techniques

The morphology and structural features of the synthesized PANI nanotubes–nanospheres were investigated using scanning electron microscopy (SEM) and Fourier–transform infrared spectroscopy (FT–IR).

#### Scanning Electron Microscopy (SEM)

SEM imaging was performed using a Quanta 650 microscope to visualize the morphological aspects of the PANI nanotubes–nanospheres. Prior to imaging, the samples were coated with a thin layer of gold using a sputter coater to increase their conductivity and enhance the imaging quality. The SEM images were acquired at various magnifications to observe the overall morphology and surface features of the nanostructures.

#### Fourier–Transform Infrared Spectroscopy (FT–IR)

FT–IR spectroscopy was employed to characterize the chemical structure and functional groups present in the PANI nanotubes–nanospheres. The FT–IR spectra were recorded using a Nicolet iS 5 FTIR Spectrometer (Thermo Scientific) in the wavenumber range of 400 to 4000 cm^–1^ with a resolution of 4 cm^–1^. The obtained FT–IR spectra were analyzed using the Omnic software (OMNIC Series Software, Thermo Scientific) to identify the characteristic absorption bands corresponding to the functional groups and chemical bonds present in the PANI nanostructures. Figure 25a–c in the Supporting Information provides detailed FT–IR spectra, peak assignments, and comparative analysis of PANI synthesized using different oxidizing agents (ferrous chloride, ferrous nitrate and ammonium persulfate). The graphical representation of the FT–IR spectra, along with the peak lists and their corresponding intensities, are included in Tables 13−15 in the Supporting Information.

### Electrical Characterization of PANI–Proteinoid Samples

**Figure 2:**
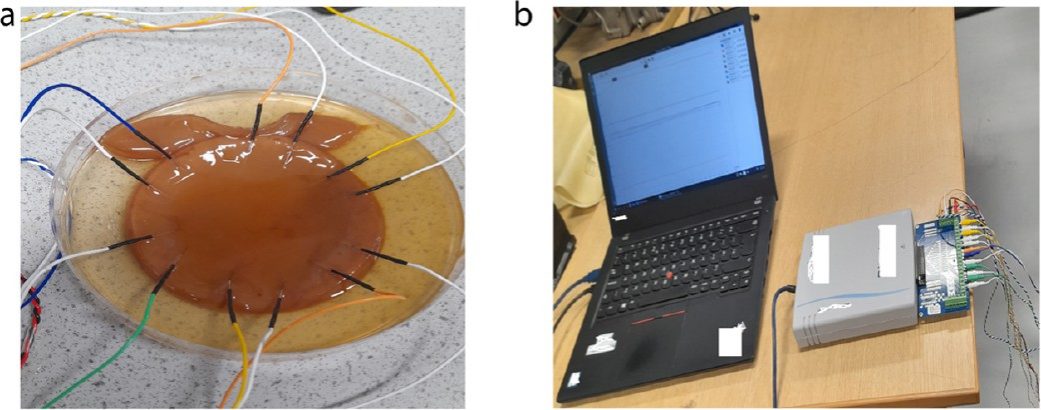
(a) Kombucha mat with embedded PANI–proteinoid solution and electrodes. The custom–made platinum–iridium coated stainless steel needle electrodes are inserted into the kombucha mat approximately 10 mm apart to record the electrical activity of the PANI–proteinoid mixture within the interconnected matrix. (b) Experimental configuration for the continuous monitoring of electrical voltage spikes. The Pico Technology ADC–24 data logger, with its 16 channels, is connected to a laptop to enable real–time monitoring of the electrical signals produced by the PANI–proteinoid samples. The 24–bit data acquisition system with high resolution allows for accurate monitoring of voltage changes while minimizing noise interference.

The recording of electrical activity from the proteinoid–PANI samples used custom–made stainless steel needle electrodes coated with platinum–iridium, which were fabricated by Spes Medica Srl. The electrodes were specifically positioned at a distance of roughly 10 mm from each other within the samples. This was done to accurately record and analyze the spread of responses across the linear distance within the interconnected matrix. We used a high–resolution 24-bit Pico Technology ADC–24 data recorder to capture the signals. This allowed us to accurately track voltage variations while reducing the effect of noise. The electrochemical cell arrangement used for conducting the tests is depicted in Figure 2.

## Results

In the Results section, we present a comprehensive analysis of the proteinoid-polyaniline (PANI) system and its interaction with stimulated neurons on various substrates. We begin by providing insights into the structural composition and properties of the PANI-proteinoid system through morphological characterization. We then conduct a statistical analysis of the proteinoid-PANI spiking behavior to understand its response to different stimuli. K-means clustering is employed to identify patterns in the PANI-proteinoid spiking activity, followed by the estimation of firing rates in proteinoid-PANI samples. We also present raster plots of neuronal spike times to visualize the temporal dynamics of the system. Additionally, we characterize the capacitance, resistance, and impedance of the proteinoid-PANI system to gain a deeper understanding of its electrical properties. Finally, we model spiking neurons of PANI-proteinoid on PEN substrate, ITO glass, and kombucha substrate to investigate the influence of different substrates on the system’s behavior.

### Morphological Characterization of the PANI–Proteinoid System: Insights into Structural Composition and Properties

**Figure 3:**
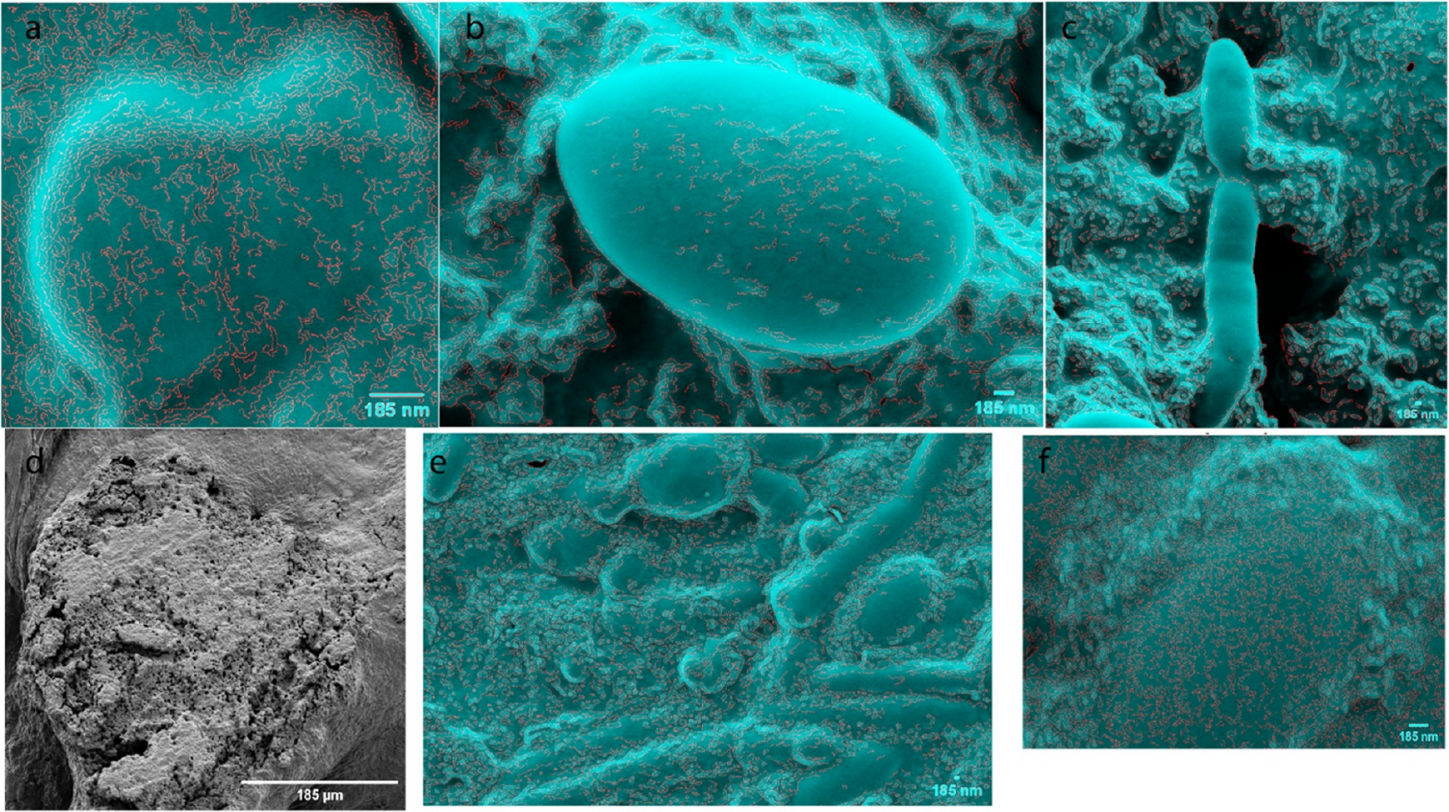
Scanning electron micrographs (SEM) of polyaniline (PANI) film deposited on a kombucha mat. The kombucha film was coated with gold. (a–c) SEM images at a 185 nm scale reveal the diverse yeast morphologies present in the kombucha mat, including (a) heart-shaped yeast, (b) yeast microspheres, and (c) elongated yeast microspheres, indicating various stages of reproduction. (d) A SEM image at a scale of 185 μm demonstrates the deposition of PANI on the yeast cells within the kombucha mat. (e, f) SEM images at a scale of 185 nm showcase the PANI powder mixed with kombucha yeast, resulting in the formation of microspheres and elongated spheres. The red color, inserted using MATLAB, highlights the edges of the yeast cells and PANI structures, providing a clear visualization of their morphological features.

Using scanning electron microscopy (SEM), the morphology and structure of the polyaniline (PANI) film deposited on the kombucha mat were analyzed. At a resolution of 185 nm, Figure 3a–c shows the different yeast shapes found in the kombucha mat. These include (a) heart-shaped yeast, (b) yeast microspheres, and (c) elongated yeast microspheres, which show different stages of reproduction. The presence of these distinct yeast morphologies suggests a complex and dynamic environment within the kombucha mat. Figure 3d demonstrates that PANI was successfully attached to the yeast cells in the kombucha mat, with a size of 185 μm. The PANI coating seems to adhere closely to the surface of the yeast cells, forming a strong bond between the two components. The combination of PANI and yeast cells in the kombucha mat indicates the possibility of synergistic effects and enhanced characteristics of the hybrid material. Furthermore, Figure 3e and f, at a scale of 185 nm, showcase the PANI nanofibers mixed with kombucha yeast, resulting in the formation of microspheres and elongated spheres. The red color, inserted using MATLAB, highlights the edges of the yeast cells and PANI structures, providing a clear visualization of their morphological features.

This illustrates the intricate connection that exists between the nanospheres and the substrate. The precise morphology and interconnectivity of the polyaniline nanostructures on the kombucha substrate are visually apparent in the SEM images, which serve as demonstration of their successful deposition and formation. The observed compatibility and interaction between the polyaniline and the kombucha surface indicate that this composite material may have considerable potential for implementation in a range of applications, such as bio–inspired electronics and neuromorphic computation.

**Figure 4:**
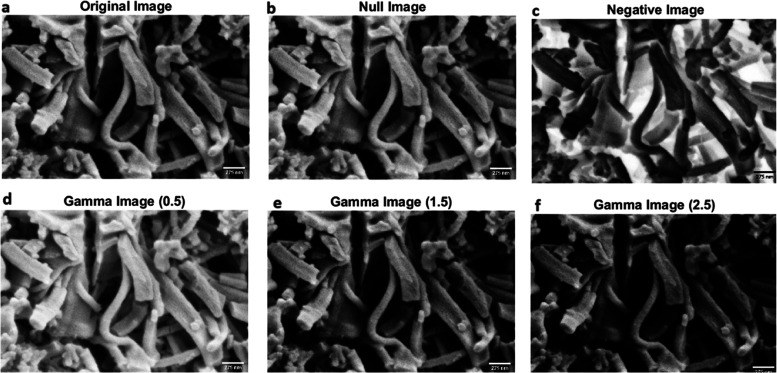
Image transformations applied to PANI fibers. (a) Original image of PANI fibers. (b) Null transformation, resulting in an unchanged image. (c) Negative transformation, inverting the pixel intensities of the original image. (d–f) γ correction transformations with different γ values: (d) γ = 0.5, resulting in a brighter image; (e) γ = 1.5, resulting in a darker image; and (f) γ = 2.5, resulting in an even darker image with increased contrast. The γ correction transformations adjust the pixel intensities non–linearly, allowing for the enhancement or suppression of different intensity ranges in the image. These transformations highlight the structural details and morphological characteristics of the PANI fibers, providing insights into their visual properties and potential for image analysis and feature extraction.

Various image modifications were used to explore the visual quality and morphological aspects of PANI fibers. Figure 4 displays the initial image of PANI fibers (a) along with the implemented transformations, which include a null transformation (b), a negative transformation (c), and γ correction transformations with varying γ values (d–f). The null transformation, denoted as *T*_null_(*I*) = *I*, where *I* represents the original picture, produces an unaltered image (Figure 4b). This serves as a reference point for comparing the impacts of other adjustments. The negative transformation, denoted as *T*_negative_(*I*) = 255 – *I*, reverses the pixel intensities of the original picture (Figure 4c). This conversion emphasizes the reciprocal arrangement of the PANI fibers, emphasizing the areas with less light and uncovering concealed complexities. γ correction transformations, denoted as *T*_γ_(*I*) = *I*^γ^, where γ represents the γ value, modify the pixel intensities in a non–linear manner. Three distinct γ values were used: γ = 0.5 (Figure 4d), γ = 1.5 (Figure 4e), and γ = 2.5 (Figure 4f). Decreasing the γ values, such as to 0.5, will make the image appear brighter. Conversely, increasing the γ values, such as to 1.5 or 2.5, would result in darker images with enhanced contrast. These modifications enable the amplification or reduction of various intensity ranges in the image, thereby exposing fine features and textural characteristics of the PANI fibers.

The converted photos offer useful insights into the structural arrangement and morphological characteristics of the PANI fibers. The negative transformation (Figure 4c) enhances the visibility of the interconnected network of fibers, emphasizing their branching and clustering patterns. The γ correction transformations (Figure 4d–f) expose the difference in fiber thickness, orientation, and density among various areas of the picture. Through the use of these image modifications, we may extract significant visual data from the PANI fibers and gain a deeper understanding of their structural characteristics. The converted images provide as the basis for additional image analysis methods, including segmentation, feature extraction, and quantitative evaluation of the fiber shape. The utilization of these transformations enables the visualization and analysis of PANI fibers, thereby providing opportunities to enhance their synthesis conditions, comprehend their growth mechanisms, and investigate their potential applications in diverse fields, including tissue engineering, drug delivery, and biosensing.

**Figure 5:**
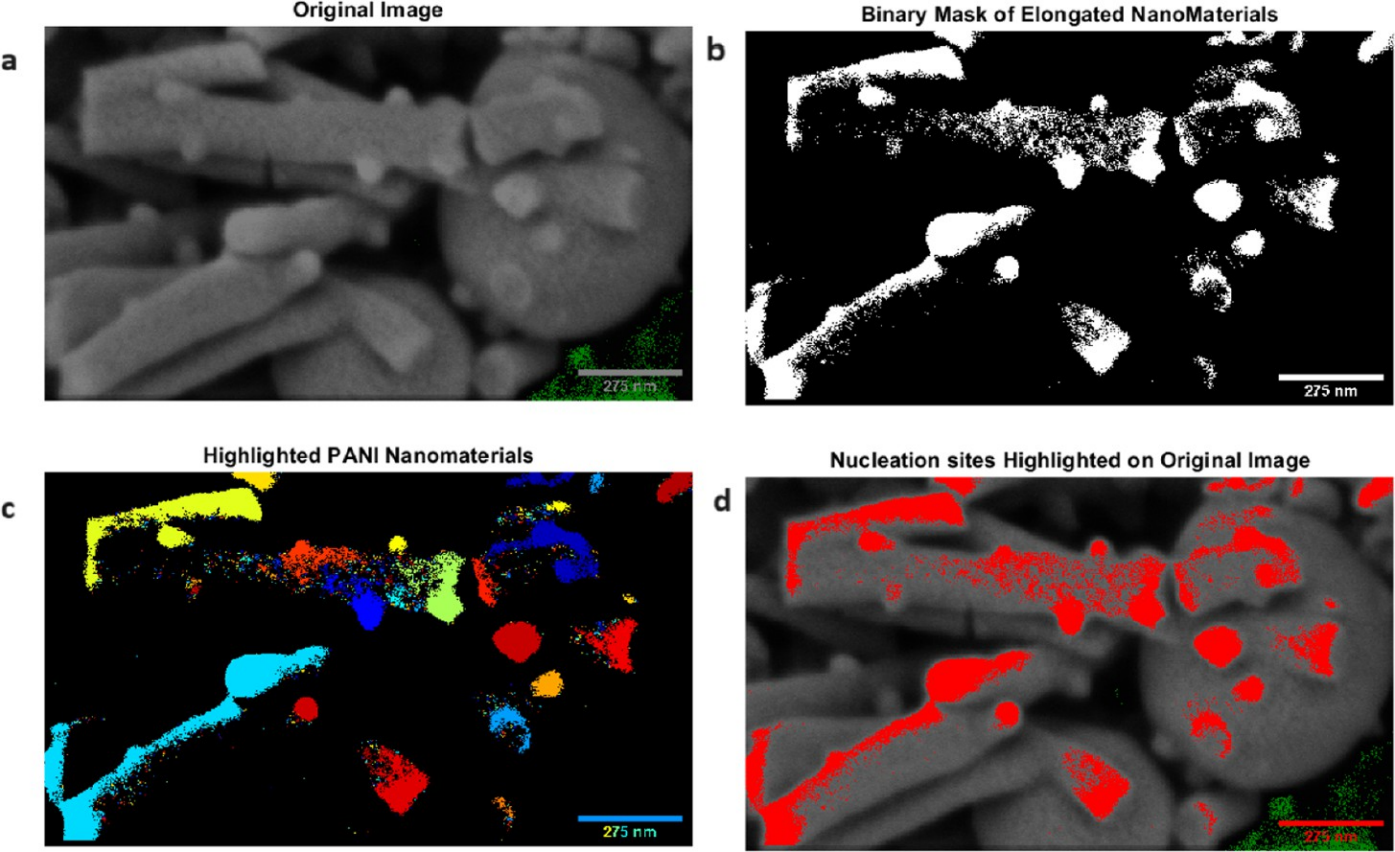
(a) This is a scanning electron microscopy (SEM) image showing the nanostructures of polyaniline (PANI). The image emphasizes the interconnected network of nanospheres and nanofibers. The image has been adjusted to improve the visual brightness and clarity of the PANI morphology. The nanospheres, which have sizes between 200 and 400 nm, are linked together by a network of nanofibers, creating a structure that is both highly porous and tightly connected. The nanofibers possess a significant aspect ratio, characterized by lengths that can reach several micrometers and diameters ranging from 50 to 100 nm. The complex relationship between the nanospheres and nanofibers forms a hierarchical structure that optimizes surface area and enables effective charge transfer. The green backdrop color has been implemented to enhance the visual contrast and highlight the structural details of the PANI nanostructures. The scale bar measures a length of 275 nm. (b) Binary Mask of Elongated NanoMaterials: This subplot shows the thresholded binary mask of the elongated PANI nanomaterials. The binary mask is created by applying a threshold value to the grayscale version of the original image, effectively separating the PANI nanostructures from the background. (c) Highlighted PANI Nanomaterials: This subplot presents the colored and labeled image of the PANI nanomaterials. Each connected component (individual PANI nanostructure) is assigned a unique color using the “jet” colormap, allowing for easy visual distinction between different nanostructures. (d) Nucleation sites Highlighted on Original Image: This subplot overlays the binary mask of the PANI nanostructures on the original SEM image using a red color. This overlay highlights the nucleation sites of the PANI nanostructures, providing a visual representation of their distribution and locations within the original image.

Figure 5 displays an enhanced scanning electron microscope (SEM) image that clearly shows the complex and interconnected structure of PANI nanospheres and nanofibers. An optimization method was performed on the SEM picture of the PANI nanostructures to improve their visual clarity and contrast. The image’s luminosity was enhanced using the subsequent equation:
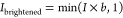
4where *I* represents the original image, *b* is the brightness factor (set to 1.5 in this case), and *I*_brightened_ is the resulting brightened image. The min function ensures that the pixel values are capped at 1 to avoid saturation. To further improve the visual contrast, a green background color was applied to the image. The green channel of the background (bg) was set to a desired intensity value (*g*) using the following equation:
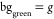
5where *g* was set to 0.8 in this case. The green background was then blended with the brightened image using the imfuse function in MATLAB, as shown in eq 6.
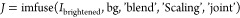
6The resulting optimized image (*J*) effectively highlights the structural details and morphological features of the PANI nanostructures. The interconnected network of polyaniline (PANI) nanospheres and nanofibers, as seen in Figure 5, has significant effects for numerous applications. The nanospheres’ large surface area and the nanofibers’ effective charge transport make this morphology well–suited for energy storage devices, such as supercapacitors and batteries. The porous structure of PANI–based electrodes allows for efficient electrolyte infiltration and ion diffusion, hence improving their electrochemical performance.

Furthermore, the interlinked network of PANI nanostructures has distinct possibilities for catalysis and sensing applications. The nanospheres and nanofibers have a significant surface area and exposed active sites, which can greatly improve the catalytic activity and sensitivity of PANI–based materials. The hierarchical construction of PANI nanostructures enables the immobilization of biomolecules or functional groups, making them highly promising for use in biosensors and chemical sensors.

The growth of hollow proteinoid microspheres as potential carriers for polyaniline (PANI) nanospheres and their interconnection with PANI nanofibers was investigated using scanning electron microscopy (SEM). Figure 29 in the Supporting Information displays a scanning electron microscope (SEM) image that showcases the distinct shape and layered composition of the proteinoid microspheres. The proteinoid microspheres, measuring between 1 and 2 μm in diameter, display a clearly visible hollow structure, as can be seen in the figure where collapsed and partially open microspheres are detected. The hollow microspheres are well–suited for carrying PANI nanospheres, offering a secure environment for encapsulation and precise release. The PANI nanospheres can be introduced into the empty interior of the proteinoid microspheres using different methods, such as in-situ polymerization or physical adsorption. This enables the formation of a composite material with improved functionality.

The proteinoid microspheres are covered with a network of PANI nanofibers that smoothly blend with the structure of the microspheres. The nanofibers have diameters of around 50 nm and lengths that can reach several micrometers. They create a conductive and interconnected network, which improves charge transfer and improves the electrical characteristics of the composite material. The incorporation of PANI nanofibers on the proteinoid microspheres’ surface enhances both conductivity and offers a substantial surface area for many applications.

The integration of hollow proteinoid microspheres with PANI nanofibers results in a distinctive hierarchical architecture that has numerous benefits. First, the empty space inside the microspheres allows for a significant amount of PANI nanospheres to be enclosed, resulting in a high capacity for loading and the ability to control the release of the nanospheres. Furthermore, the interlinked network of PANI nanofibers facilitates effective charge transfer across the composite material, hence improving its electrical conductivity and electrochemical properties. The proteinoid microspheres and PANI nanofibers possess a hierarchical structure that provides a large surface area. This characteristic is advantageous for several applications including catalysis, sensing, and energy storage.

### Statistical Analysis of Proteinoid–PANI Spiking Behavior

This subsection contains an analysis of the trends underlying the Proteinoid–PANI samples’ spiking behavior. The Appendix in the Supporting Information contains comparative graphs illustrating the activity of stimulated input neurons and the resulting spike output of proteinoids–PANI. By implementing a moving average methodology, our objective is to detect trends and variations in the spiking activity as it evolves. Figure 30 in the Supporting Information displays subplots of moving averages that emphasize the temporal trends and patterns of PANI–proteinoid activity for various spiking patterns. The blue lines in each subplot depict the moving average of spike amplitudes over a window of 10 time steps, offering a smoothed picture of the proto–brain responses. The addition of these subplots enables a direct visual comparison of the spiking dynamics under various experimental settings, which reveals differences in both amplitude and temporal properties.

Figure 31 in the Supporting Information presents a thorough comparison of spike amplitudes between input neurons and PANI–proteinoid samples using box plots. When considering all types of spiking behaviors, the input neurons show wider distributions and a greater number of data points that deviate from the norm, suggesting larger ranges between the first and third quartiles (IQR) and more significant voltage or spike amplitudes. On the other hand, the PANI–proteinoid samples consistently exhibit narrower distributions and fewer exceptional values, indicating more focused and regular ranges of voltage or spike amplitudes. The box plot metrics illustrate the improved stability and consistency of spiking behavior in PANI–proteinoid samples when compared to input neurons.

Thalamocortical stimulation results in input neurons having a broader range of voltage distribution (IQR: −67.83 to −52.97 mV, median: −60.92 mV) compared to PANI–proteinoid samples (IQR: 2.47 to 2.99 mV, median: 2.73 mV). Likewise, the rise in accommodation indicates a higher possibility for variation in the input neurons compared to the proteinoid–PANI samples. PANI–proteinoid samples exhibit the capacity to maintain a shorter and more consistent range of spike amplitudes compared to input neurons, as demonstrated by phasic, mixed mode, tonic, and induced spiking. The comparison of spike integrators demonstrates the enhanced stability of PANI–proteinoid samples. These samples exhibit a higher average value (−79.90 vs 0.34 mV), a narrower range (−89.15 to 73.13 mV vs −1.39 to 5.50 mV), and a lower standard deviation (11.29 vs 0.45 mV) compared to the input spike integrator.

Table 16 in the Supporting Information presents a concise overview of the statistical characteristics of spike behaviors, such as skewness and kurtosis, for both input neurons and PANI–proteinoid samples. The data from the table demonstrates that PANI–proteinoid samples consistently display decreased skewness and kurtosis values compared to input neurons in all spiking behaviors. These findings suggest that the spike amplitude distributions of PANI–proteinoid samples exhibit greater symmetry and have less pronounced tails compared to the spike amplitude distributions of input neurons. Additionally, the PANI–proteinoid samples exhibit consistently smaller standard deviations of spike amplitudes compared to the input neurons. This finding further supports the idea that the spiking behavior of PANI–proteinoid samples is characterized by reduced variability and improved consistency.

The results show that the PANI–proteinoid system can effectively process and regulate spiking activity in different behaviors, turning highly unpredictable inputs into more predictable and consistent output responses. The increased stability and decreased variability in the size of electrical signals in PANI–proteinoid samples demonstrate their potential as a reliable and strong foundation for neuromorphic computing applications. The moving average subplots, box plot comparisons, and statistical analysis offer different perspectives on the spiking kinetics and properties of PANI–proteinoid samples in respect to input neurons. The findings enhance our knowledge of the PANI–proteinoid system’s capacity to produce enduring and regular spiking patterns, positioning it as an intriguing option for brain–inspired information processing and computation. Detailed analysis of each spiking analysis for each neuron can be found in Figures 17−22 in the Supporting Information.

### K-means Clustering of Pani–Proteinoid Spiking Activity

**Figure 6:**
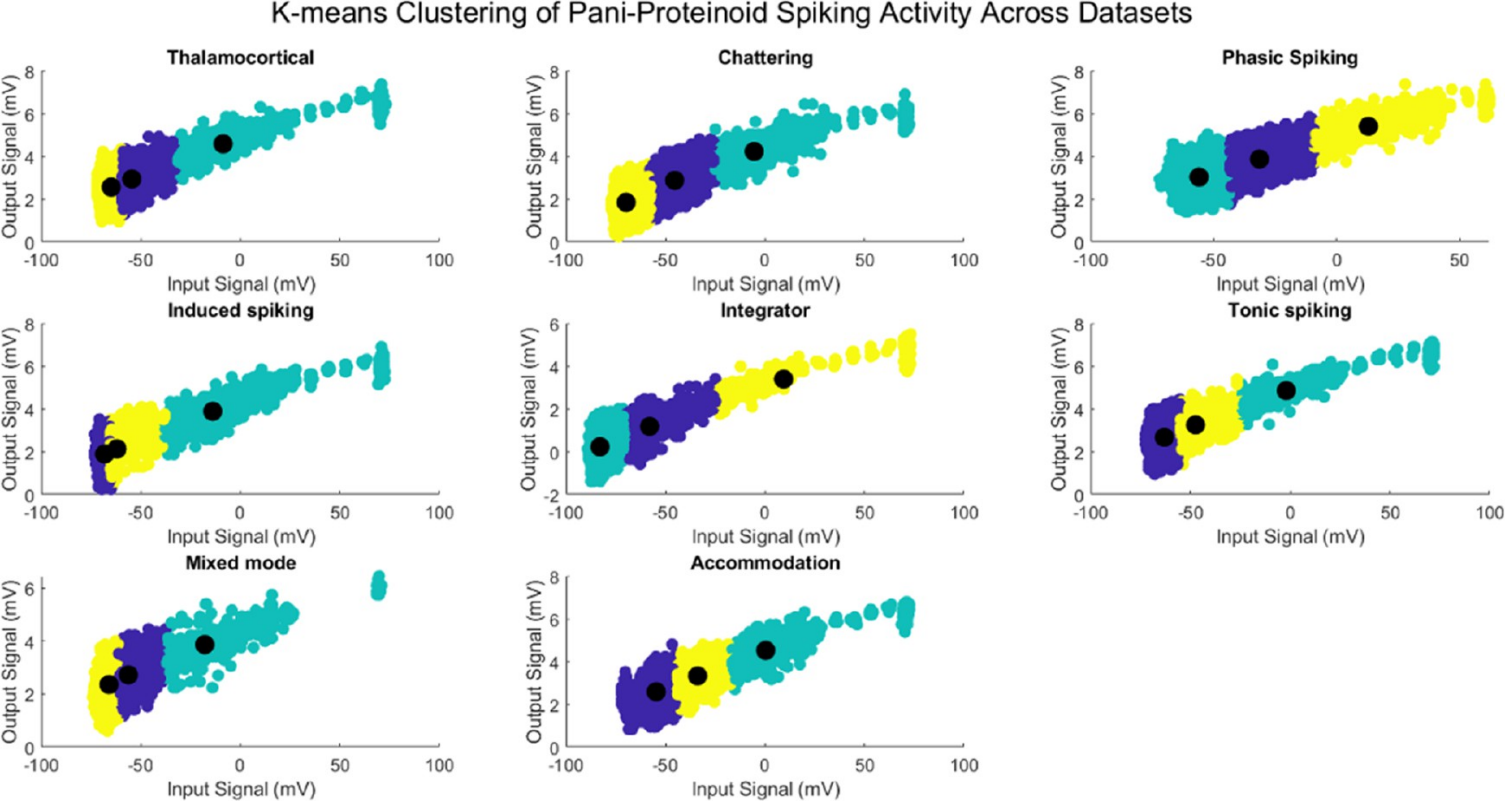
K-means clustering of PANI–proteinoid spiking activity across data sets. Each subplot depicts a spike behavior: Thalamocortical, Chattering, Phasic, Induced, Integrator, Tonic, Mixed mode, and Accommodation. The scatter plots depict data point distribution in potential–time feature space, with colors denoting clusters. Black filled circles overlaid on cluster centroids show each cluster’s mean potential and time. Spiking activity clusters within each data set show PANI–proteinoid reactions’ various patterns and features. The centroids summarize each cluster’s average potential and temporal features, allowing quantitative comparison of spiking behavior across data sets. The K-means clustering method captures the structure and variability of PANI–proteinoid spiking data, allowing the identification of typical patterns and their potential and temporal properties.

K-means clustering analysis was conducted on the PANI–proteinoid spiking activity in multiple data sets to uncover unique patterns and characteristics of the system’s response to varied spiking behaviors. Figure 6 displays the clustering results for each data set, illustrating the setup of data points in the potential–time feature space and the associated cluster centroids.

Each subplot in Figure 6 depicts a distinct spiking behavior, such as Thalamocortical, Chattering, Phasic spiking, Induced spiking, Integrator, Tonic spiking, Mixed mode, and Accommodation. The scatter plots visually display the arrangement of data points, with colors representing the assigned clusters. The cluster centroids, depicted as black filled circles, offer an overview of the mean potential and temporal characteristics of each cluster.

The clustering analysis demonstrates the presence of distinct clusters of spiking activity in each sample, emphasizing the varied patterns and features of PANI–proteinoid response. In the Thalamocortical data set, three separate clusters are detected in the potential–time feature space. The centroids of these clusters are found at (−54.5520, 2.9359), (−8.6907, 4.5850), and (−64.9216, 2.5557), as shown in Figure 6a. The Chattering data set (Figure 6b) also shows three clusters with centroids located at (−45.4117, 2.8735), (−5.5061, 4.2265), and (−69.7813, 1.8415).

The cluster centroids offer a quantitative evaluation of the spiking behavior across various data sets. By analyzing the centroid values, we may examine the fluctuations in the mean potential and temporal properties of the PANI–proteinoid reactions. The Phasic spiking data set (Figure 6c) exhibits centroids at (−31.4006, 3.8746), (−55.9273, 3.0289), and (12.8904, 5.4028), indicating a wider range of potential values compared to the Induced spiking data set (Figure 6d) with centroids at (−68.2052, 1.8892), (−13.8883, 3.8894), and (−62.0676, 2.1319).

The K-means clustering method accurately captures the intrinsic structure and variability in the PANI–proteinoid spiking data, making it easier to identify representative spiking patterns and their accompanying potential and temporal properties. Through the identification of separate clusters within each data set, we can enhance our comprehension of the varied reactions of the PANI–proteinoid system to various spiking behaviors and acquire knowledge about the fundamental mechanisms that drive its electrical activity. The results illustrate the ability of the PANI–proteinoid system to produce unique and reliable patterns of electrical activity in response to different stimuli, highlighting its potential as a customizable substrate for neuromorphic computing. Clustering analysis offers a structure for describing and contrasting the spiking behavior under various experimental settings, facilitating deeper exploration of the dynamics and functioning of the PANI–proteinoid system. Aside from conducting clustering analysis, we also examined the temporal dynamics and consistency of the spiking activity in the PANI–proteinoid system. This was done by computing the Pearson correlation coefficients between the membrane potential and time for each data set. The correlation coefficients for the various spiking behaviors are displayed in Table 18 in the Supporting Information. Correlation coefficients offer a numerical evaluation of the intensity and direction of the linear relationship between the membrane potential and time variables. Throughout all the data sets, we saw significant correlation coefficients ranging from 0.8514 to 0.9630, suggesting a robust positive connection. It suggests that over time, the membrane potential of the PANI–proteinoid system shows a tendency to consistently and predictably develop. The Chattering data set demonstrates a correlation coefficient of 0.9630, indicating an extremely strong linear link between membrane potential and time. Conversely, the Mixed Mode data set exhibits a correlation coefficient of 0.8514, indicating a strong positive relationship. The results demonstrate the strong temporal relationship between the membrane potential in the PANI–proteinoid system, regardless of the specific spiking behavior. The strong correlation coefficients observed in various spiking behaviors indicate that the PANI–proteinoid system has the capability to produce consistent and replicable patterns of electrical activity. The consistent and predictable alterations in the membrane potential over time highlight the promise of the PANI–proteinoid system as a dependable and customizable substrate for neuromorphic computing.

In order to provide a more detailed description of the spiking activity in each data set, Table 17 in the Supporting Information displays the cluster centroids derived from the K-means clustering analysis. The cluster centroids are denoted by tuples consisting of the potential (in millivolts) and time (in seconds), which serve as a concise representation of the average potential and temporal attributes of each cluster. Each data set includes three cluster centroids, which depict the typical spiking patterns together with their related potential and time values. The centroids provide a quantitative comparison of the spiking behavior under various experimental settings. The variations in the centroid values among data sets emphasize the diversity and distinctiveness of the PANI–proteinoid reactions to various stimuli and spiking patterns. The cluster centroids act as a point of reference for understanding the characteristic potential and temporal features of the spiking activity in each data set. Through the analysis of these centroids, we can obtain valuable information about the dynamics and properties of the PANI–proteinoid system’s responses to different spiking behaviors. This information enhances the understanding and evaluation of the system’s performance in various experimental conditions.

### Estimation of Firing Rates in Proteinoid–PANI Samples

**Figure 7:**
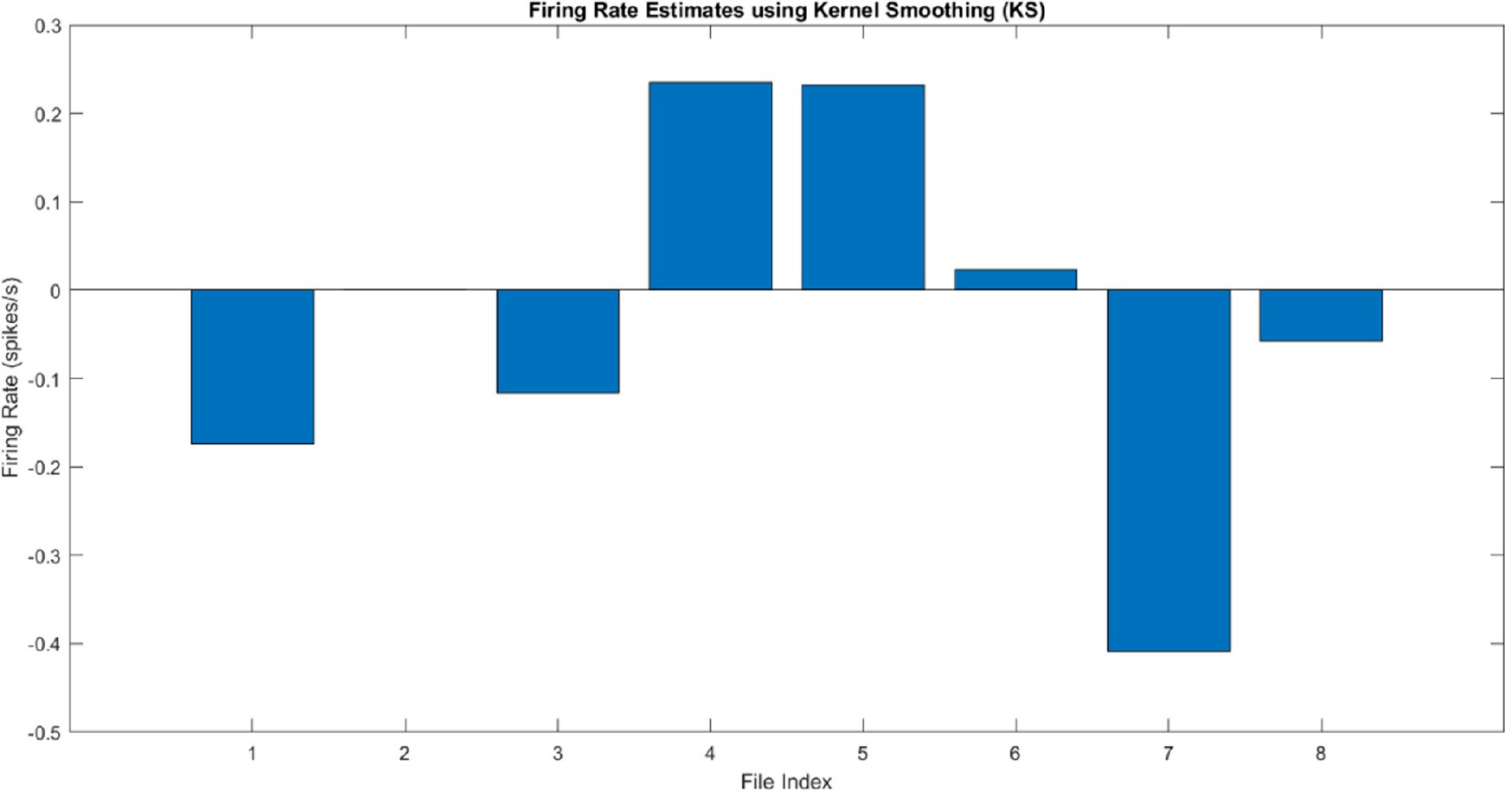
Mean firing rates of proteinoid-PANI samples for different spiking behavior data sets. The bar graph shows the mean firing rates (in spikes/s) for each data set, including thalamocortical, chattering, phasic spiking (FS), induced spiking, integrator, tonic spiking, mixed mode, and accommodation. The overall mean firing rate across all neurons is also displayed. Negative firing rates indicate the presence of inhibitory or suppressive spiking activity in some data sets.

The firing rates of the proteinoid–PANI samples were estimated for each spiking behavior data set to characterize the system’s spiking activity. Figure 7 presents the mean firing rates calculated for each data set, along with the overall mean firing rate across all neurons.

The results reveal varying mean firing rates across the different spiking behavior data sets. The thalamocortical data set exhibits a mean firing rate of −0.17 spikes/s, indicating the presence of inhibitory or suppressive spiking activity. Similarly, the mixed mode data set shows a mean firing rate of −0.41 spikes/s, suggesting a predominance of inhibitory spiking behavior. However, the induced spiking and integrator records show average firing rates of 0.24 and 0.23 spikes/s, respectively, suggesting the presence of excitatory spiking activity. The mean firing rates of the chattering and tonic spiking data sets are nearly zero (−0.00 and 0.02 spikes/s, respectively), indicating a state of equilibrium between excitatory and inhibitory spiking activity. The phasic spiking (FS) and accommodation data sets exhibit average firing rates of −0.12 and −0.06 spikes/s, respectively, suggesting the occurrence of inhibitory spiking behavior. The average firing rate of all neurons is determined to be −0.03 spikes/s, indicating a small prevalence of inhibitory spiking activity in the proteinoid–PANI samples. The results offer valuable insights into the varied spiking behavior of the proteinoid–PANI samples and emphasize the system’s capacity to produce both excitatory and inhibitory spiking activity. Estimating firing rates helps to describe the spiking dynamics of the proteinoid–PANI system and its ability to imitate different neural behaviors.

The existence of negative firing rates in some data sets indicates the occurrence of inhibitory or suppressive spiking activity, which is a crucial element of neural processing in biological systems. The proteinoid–PANI samples possess the capability to display both excitatory and inhibitory spiking activity, which showcases their versatility and potential for executing intricate neuromorphic computations.^33^

### Raster Plots of Neuronal Spike Times in Proteinoid–PANI Samples

**Figure 8:**
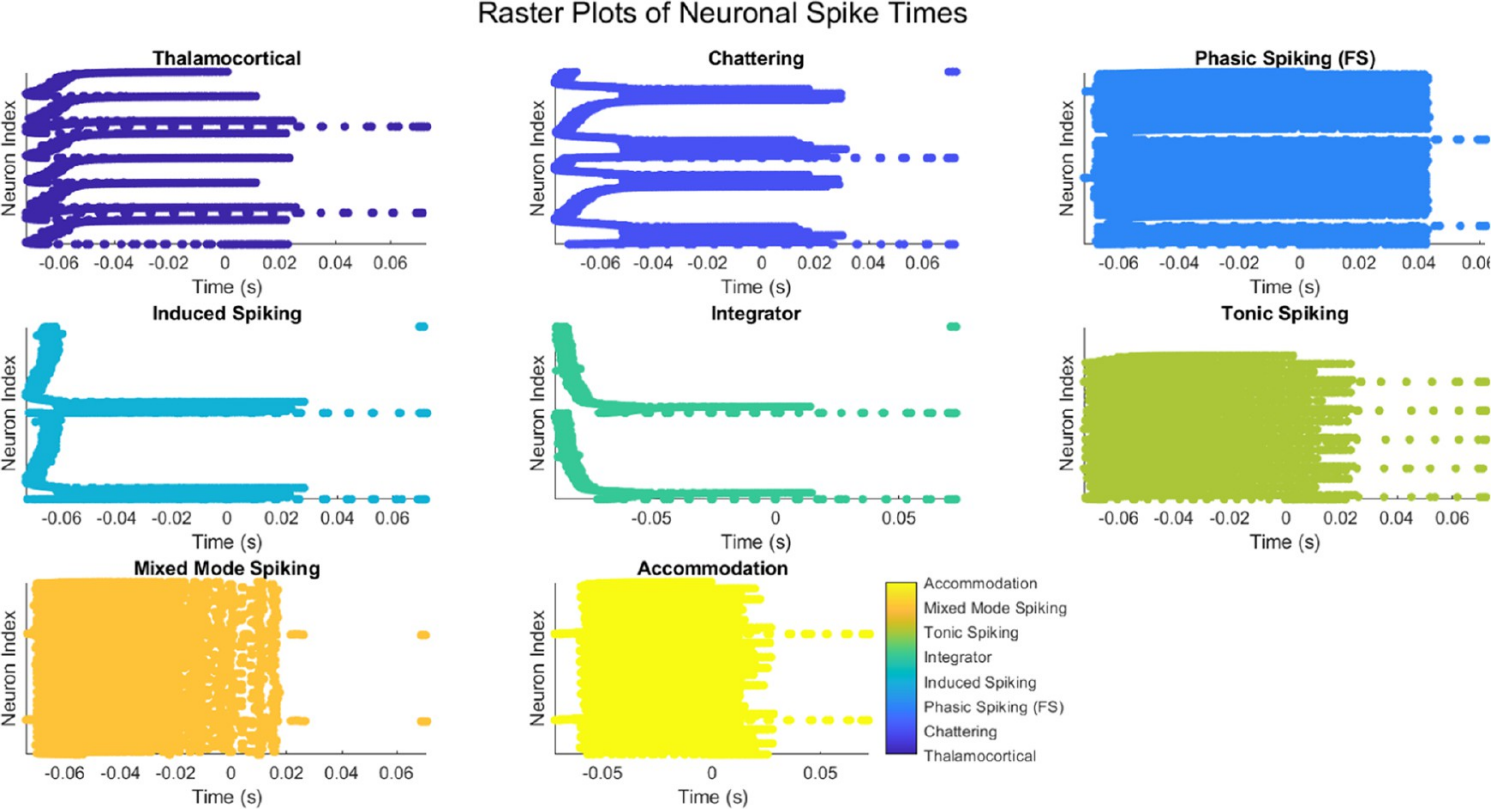
Raster plots of neuronal spike times for different spiking behavior data sets in proteinoid–PANI samples. Each subplot represents a specific data set, with the *x*–axis indicating time (in seconds) and the *y*–axis representing the neuron index. Each dot in the raster plot corresponds to a spike event of a particular neuron at a specific time point. The subplots are arranged in a 3 × 3 grid for visual comparison of the spiking patterns across different data sets.

In order to visually represent the time–based patterns of spiking activity in the proteinoid–PANI samples, raster plots were created for each data set that captured the spiking behavior. Figure 8 displays the raster plots of neural spike timings for several data sets, such as thalamocortical, chattering, phasic spiking (FS), induced spiking, integrator, tonic spiking, mixed mode, and accommodation.

Each dot in the raster plots corresponds to a spike event of a distinct neuron at a particular time point. The *x*–axis denotes the length of time in seconds, while the *y*–axis indicates the index of the neuron. The subplots are organized in a 3 × 3 grid, enabling a visual analysis of the spiking patterns across several data sets. The raster plots offer excellent insights into the temporal arrangement and synchronization of spiking activity in the proteinoid–PANI samples. By analyzing the density and arrangement of dots on the time axis, we can detect times of heightened or reduced spiking activity, as well as any observable patterns or rhythms in the spiking behavior. As an illustration, the thalamocortical data set (subplot 1) displays a sparsely distributed pattern of spikes, where certain neurons exhibit occasional spiking activity. On the other hand, the chattering data set (subplot 2) has a denser and more consistent firing pattern among neurons, suggesting a greater degree of synchronization. The data set labeled as phasic spiking (FS) in subplot 3 has clear clusters of spikes that are separated by periods of decreased activity, indicating the presence of phasic firing patterns. The spiking data set generated by induction (subplot 4) has a more dispersed distribution of spikes, suggesting a less coordinated spiking pattern.

The integrator data set (subplot 5) shows a gradual rise in spiking activity over time, with neurons displaying a continuous increase in spiking events. The tonic spiking data set (subplot 6) displays a consistent and prolonged spiking activity among neurons, indicating a tonic firing pattern. The data set in subplot 7 exhibits a mixture of tonic and phasic spiking patterns, where certain neurons display continuous activity while others demonstrate irregular bursts of spikes. The accommodation data set (subplot 8) demonstrates a progressive decline in spiking activity over time, suggesting a degree of spike frequency adaptation. These raster plots offer a complete summary of the varied spiking patterns and temporal dynamics found in the proteinoid–PANI samples across distinct data sets of spiking behavior. Visualizing spike timings allows for the detection of distinct firing patterns, levels of synchronization, and temporal fluctuations in neural activity. Additional examination and measurement of the raster plots can provide useful understanding of the fundamental mechanisms and factors that affect the spiking behavior of the proteinoid–PANI samples. This data can enhance understanding of the system’s neuromorphic capacities and direct the advancement of proteinoid–PANI–based neuromorphic computing applications.

### Capacitance, Resistance, and Impedance Characterization of the Proteinoid–PANI System

**Figure 9:**
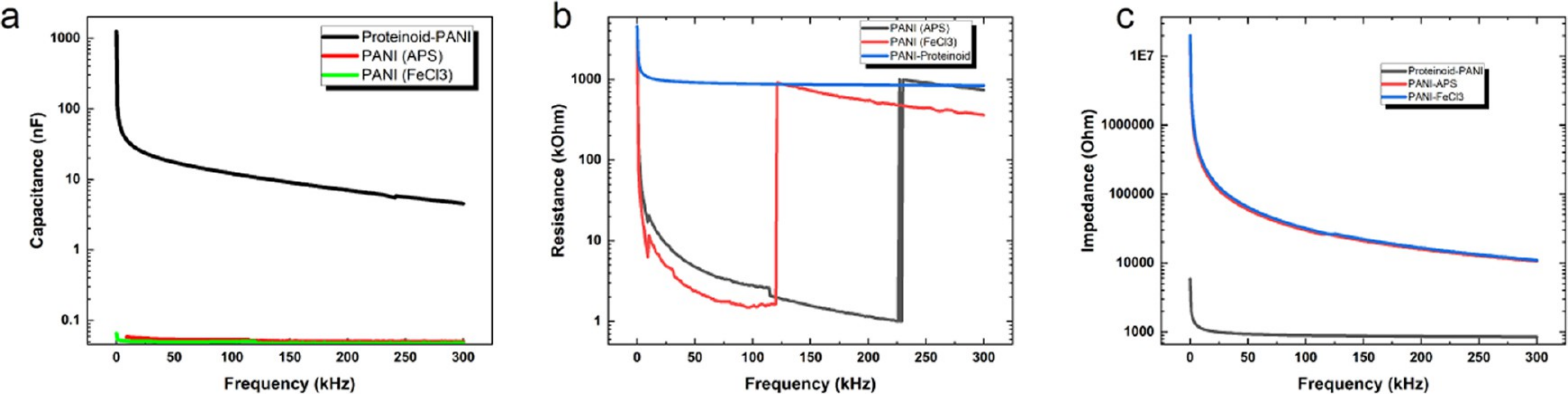
Frequency-dependent electrical properties of Proteinoid-PANI, Proteinoid-FeCl_3_, and Proteinoid-APS samples. (a) Capacitance (nF) as a function of frequency (kHz). The capacitance decreases with increasing frequency for all three samples, exhibiting a typical capacitive behavior. The Proteinoid-PANI sample shows the highest capacitance values, followed by Proteinoid-FeCl_3_ and Proteinoid-APS samples. (b) Resistance (kΩ) as a function of frequency (kHz). The Proteinoid-FeCl_3_ sample undergoes an abrupt change in resistance from 1.959 kΩ at 122.1 kHz to 900.5 kΩ at 123.1 kHz, indicating a significant alteration in its electrical properties within this narrow frequency range. Similarly, the Proteinoid-APS sample experiences a notable change in resistance from 1.005 kΩ at 229 kHz to 979.1 kΩ. The sudden variations in resistance indicate the presence of resonance or relaxation phenomena in the Proteinoid-FeCl_3_ and Proteinoid-APS samples. The Proteinoid-PANI sample, on the other hand, exhibits a relatively smooth variation in resistance with frequency, indicating a more stable and consistent electrical behavior. (c) Impedance (Ω) as a function of frequency (kHz). The results demonstrate the numerous electrical properties of the proteinoid-PANI samples and their potential for use in electronic devices and sensors that can selectively operate at specific frequencies.

Impedance spectroscopy measurements were used to study the frequency–dependent electrical properties of Proteinoid–PANI, Proteinoid–FeCl_3_, and Proteinoid–APS samples. Figure 9 displays the relationship between the capacitance, resistance and impedance of the samples in relation to the frequency. As shown in Figure 9a, the capacitance of all three samples decreases as the frequency increases, demonstrating a characteristic capacitive behavior.^34^ The Proteinoid–PANI sample has the highest capacitance values throughout the entire frequency range, showing its better capacity to store charge compared to the Proteinoid–FeCl_3_ and Proteinoid–APS samples. The increased capacitance can be attributed to the combined effect of the proteinoid and PANI components which promote effective charge accumulation and distribution in the composite material.^35^ The samples’ resistance behavior, as shown in Figure 9b, exhibits clear changes depending on the frequency. The Proteinoid–FeCl_3_ sample exhibits a sudden shift in resistance, changing from 1.959 kΩ at 122.1 kHz to 900.5 kΩ at 123.1 kHz. This indicates a notable variation in its electrical characteristics within this specific frequency range. In a similar manner, the Proteinoid–APS sample undergoes a significant change in resistance, increasing from 1.005 kΩ at 229 kHz to 979.1 kΩ. The sudden fluctuations in resistance observed in the Proteinoid–FeCl_3_ and Proteinoid–APS samples suggest the occurrence of resonance or relaxation phenomena.^36^ In contrast, the Proteinoid–PANI sample exhibits a relatively smooth variation in resistance with frequency, indicating a more stable and consistent electrical behavior. To further analyze the capacitive properties of the samples, box plot comparisons were performed, as shown in Figure 32 in the Supporting Information. In the Proteinoid–FeCl_3_ sample, there was a significant resistance shift from 1.959 kΩ at 122.1 kHz to 900.5 kΩ at 123.1 kHz. This change can be attributed to the presence of polarons and bipolarons in the PANI structure, which are formed as a result of acid doping with HCl. When PANI is doped with an acid, it undergoes a reaction where protons are added to the polymer backbone. This leads to the production of polarons (radical cations) and bipolarons (dications).^37−39^ These charge carriers contribute to the increased electrical conductivity of PANI. The proteinoid–PANI sample exhibits a peak capacitance of 1242.00 nF at a frequency of 0.02 kHz and a minimum capacitance of 4.52 nF at a frequency of 300 kHz. The average capacitance of the proteinoid-PANI sample is 16.23 nF, with a standard deviation of 71.66 nF, suggesting a significant variation in capacitance values. The improved capacitive behavior of the proteinoid–PANI sample can be linked to the combination of PANI nanostructures’ large surface area and conductivity with the self–assembly and charge–trapping characteristics of the proteinoid.^40^

In contrast, the PANI–APS and PANI–FeCl_3_ samples demonstrate significantly lower capacitance values, averaging at 0.05 nF with a standard deviation of 0.01 nF. The PANI–APS sample exhibits a peak capacitance of 0.06 nF at a frequency of 9.098 kHz and a minimum capacitance at a frequency of 0.1006 kHz. On the other hand, the PANI–FeCl_3_ sample demonstrates a maximum capacitance of 0.07 nF at a frequency of 0.1225 kHz and a minimum capacitance of 0.05 nF at a frequency of 173.1 kHz. The results emphasize the unique capacitive characteristics of the proteinoid–PANI sample and its potential for use in energy storage and capacitive sensing applications.^41^

**Figure 10:**
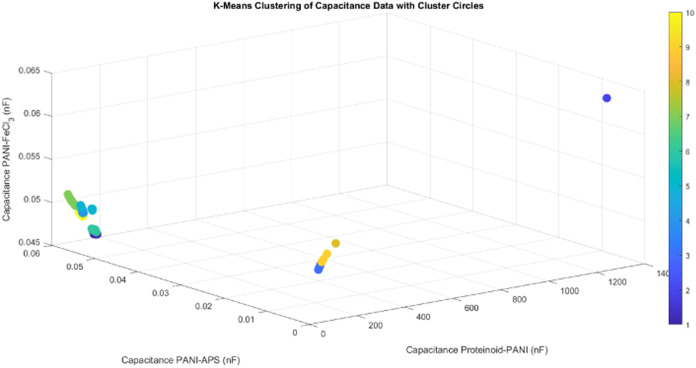
K-means clustering of capacitance data for Proteinoid–PANI, PANI–APS, and PANI–FeCl_3_ samples. The three-dimensional (3D) scatter plot shows the clustering results, where each data point represents a sample, and the color indicates the assigned cluster. A total of 10 clusters were identified based on the capacitance values of the three samples. The Proteinoid–PANI–FeCl_3_ sample exhibits the highest mean capacitance of 16.23 nF with a standard deviation of 71.66 nF, indicating a wide range of capacitance values. In contrast, both PANI–APS and PANI-FeCl_3_ samples have significantly lower mean capacitance values of 0.05 nF, with standard deviations of 0.01 and 0.00 nF, respectively. The clustering analysis reveals distinct groups of samples based on their capacitance characteristics, providing insights into the similarities and differences among the Proteinoid–PANI, PANI–APS, and PANI–FeCl_3_samples. The results suggest that the Proteinoid–PANI sample possesses unique capacitive properties compared to the other two samples, which can be attributed to the synergistic effect of the proteinoid and PANI components in the composite material.

The k-means clustering approach was utilized to analyze the capacitance data of Proteinoid–PANI, PANI–APS, and PANI–FeCl_3_ samples in order to find unique groups based on their capacitive properties (Figure 10). The algorithm’s goal is to divide the data into K clusters, with each data point assigned to the cluster that has the closest centroid. The objective function minimized by the k-means algorithm is given by
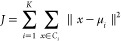
7Where *C*_*i*_ represents the *i*-th cluster μ_*i*_ is the centroid of cluster *C*_*i*_ and *x* is a data point belonging to *C*_*i*_. Figure 10 presents the results of the clustering analysis, where each data point represents a sample, and the color indicates the assigned cluster. The clustering analysis reveals 10 distinct clusters, highlighting the similarities and differences among the Proteinoid–PANI, PANI–APS, and PANI–FeCl_3_.

The clustering analysis serves as a foundation for evaluating the capacitive characteristics of various composites based on PANI and understanding the impact of different dopants and additives on their performance. This information can provide direction for selecting materials and designing composite systems for specific applications that require high capacitance and distinctive capacitive responses.

### Modeling Spiking Neurons of PANI–Proteinoid on PEN Substrate, ITO Glass, and Kombucha substrate

This section outlines the findings of our study on the modeling and characterization of spiking neurons using PANI–proteinoid composites. These composites were deposited on different substrates such as polyethylene naphthalate (PEN), indium tin oxide (ITO) glass, and kombucha–derived bacterial cellulose. The main aim of this research is to investigate how the features of the substrate affect the electrical and spiking behavior of PANI–proteinoid neurons. Additionally, the study aims to determine the most appropriate substrate for the development of neuromorphic devices. Figure 12 displays boxplots that provide a summary of the potential values for each spiking behavior on the three substrates. The Table 2 displays the statistical data of potential (mV) for various spiking behaviors on PEN, Glass ITO, and kombucha substrates. The table presents an in-depth analysis of the highest, lowest, average, variability, first quartile, and third quartile values of the potential for each spiking behavior on the three substrates. The boxplots display clear patterns of potential distribution among the substrates. When examining the PEN and Glass ITO substrates, a broader spectrum of potential values is noticed, with a significant predominance of negative potentials. This suggests that there is a higher occurrence of inhibitory or hyperpolarizing reactions in the PANI–proteinoid networks on these surfaces. The median potential values for the majority of spiking behaviors on PEN and Glass ITO substrates exhibit a shift toward negative values, providing additional evidence for supporting this result.

**Table 2:** Statistics of Potential (mV) for Different Spiking Behaviors on Substrates

spiking behavior	max	min	mean	std	first quartile	third quartile
PEN Substrate						
accommodation	1.53	–16.03	–11.44	1.49	–12.24	–11.27
chattering	0.89	–16.14	–12.19	1.90	–13.62	–11.32
mixed mode	10.13	–12.99	–0.03	6.81	–4.79	7.49
phasic	0.38	–15.45	–11.24	1.97	–12.53	–10.75
thalamocortical	0.67	–15.97	–12.49	1.13	–13.10	–12.24
tonic	0.32	–15.74	–10.48	1.98	–11.61	–9.95
induced	1.64	–15.97	–12.68	1.39	–13.27	–12.76
integrator	0.44	–10.81	–9.32	0.71	–9.66	–9.26
ITO–glass Substrate						
accommodation	0.44	–10.58	–7.63	1.01	–8.17	–7.54
chattering	3.36	–16.54	–12.20	1.90	–13.62	–11.32
mixed mode	0.21	–7.08	–5.16	0.41	–5.36	–5.01
phasic	–0.20	–10.81	–7.62	1.35	–8.51	–7.25
thalamocortical	0.49	–10.81	–8.37	0.76	–8.80	–8.23
tonic	0.21	–7.08	–4.25	0.81	–4.73	–4.04
induced	0.38	–11.27	–8.62	0.95	–9.03	–8.69
integrator	0.67	–11.73	–9.52	0.73	–9.89	–9.43
Kombucha Substrate						
accommodation	3.68	0.26	1.45	0.32	1.26	1.52
chattering	3.98	0.22	1.61	0.42	1.29	1.87
mixed mode	3.64	–1.19	0.78	0.25	0.65	0.89
phasic	4.40	1.17	2.35	0.18	2.26	2.41
thalamocortical	3.50	–1.09	0.99	0.26	0.85	1.06
tonic	4.72	1.51	2.81	0.36	2.61	2.93
induced	4.63	1.30	2.45	0.29	2.30	2.47
integrator	4.37	0.65	1.76	0.24	1.63	1.81

By comparing the potential statistics of different substrates, we may observe how the substrate affects the spiking properties of PANI–proteinoid. The PEN substrate exhibits a diverse range of potential values for the majority of spiking behaviors, encompassing both negative and positive potentials throughout a wide spectrum. The average potential values for the PEN substrate are typically negative, suggesting a prevalence of inhibitory or hyperpolarizing reactions. The standard deviation values indicate a significant level of variability in the potential responses on the PEN substrate.

The potential statistics on the Glass ITO substrate show a similar pattern to the PEN substrate, with a broad range of potential values and mainly negative mean potentials for most spiking behaviors. Nevertheless, the standard deviation values for the Glass ITO substrate are slightly lower in comparison to the PEN substrate, suggesting a relatively more uniform potential response.

Remarkably, the kombucha substrate exhibits distinct statistical potential when compared to the PEN and Glass ITO substrates. For most spiking behaviors, the range of potential values on the kombucha substrate is shorter, with the lowest and maximum values being closer to zero. The average potential values on the kombucha substrate are also closer to zero, indicating a state of equilibrium between excitatory and inhibitory responses. The standard deviation values for the kombucha substrate tend to be smaller, suggesting a more consistent and less chaotic potential response.

The variations in the potential statistics among the substrates can be attributed to multiple reasons. The adhesion, growth, and electrical coupling of the PANI–proteinoid network may be affected by the surface characteristics, roughness, and conductivity of the substrates. The PEN and Glass ITO substrates, being synthetic materials, may possess distinct surface properties in comparison to the kombucha substrate, which is produced from a biological source. The unique composition and microstructure of the kombucha substrate may create a more advantageous setting for the PANI–proteinoid network, resulting in more stable and uniform potential responses.

Additionally, variations in the electrical characteristics of the substrates, such as their dielectric constant and conductivity, can impact the capacitive connection and transfer of charge between the PANI–proteinoid network and the substrate. The kombucha substrate, as a natural biopolymer, may possess unique electrical characteristics that enhance the efficiency and stability of its interaction with the PANI–proteinoid network.

The findings emphasize the significance of choosing the right substrate to control the spiking properties of PANI-proteinoid networks. The kombucha substrate shows promise as a potential candidate for obtaining more balanced and constant potential responses. This could be beneficial for the development of reliable and resilient neuromorphic devices.

**Figure 11:**
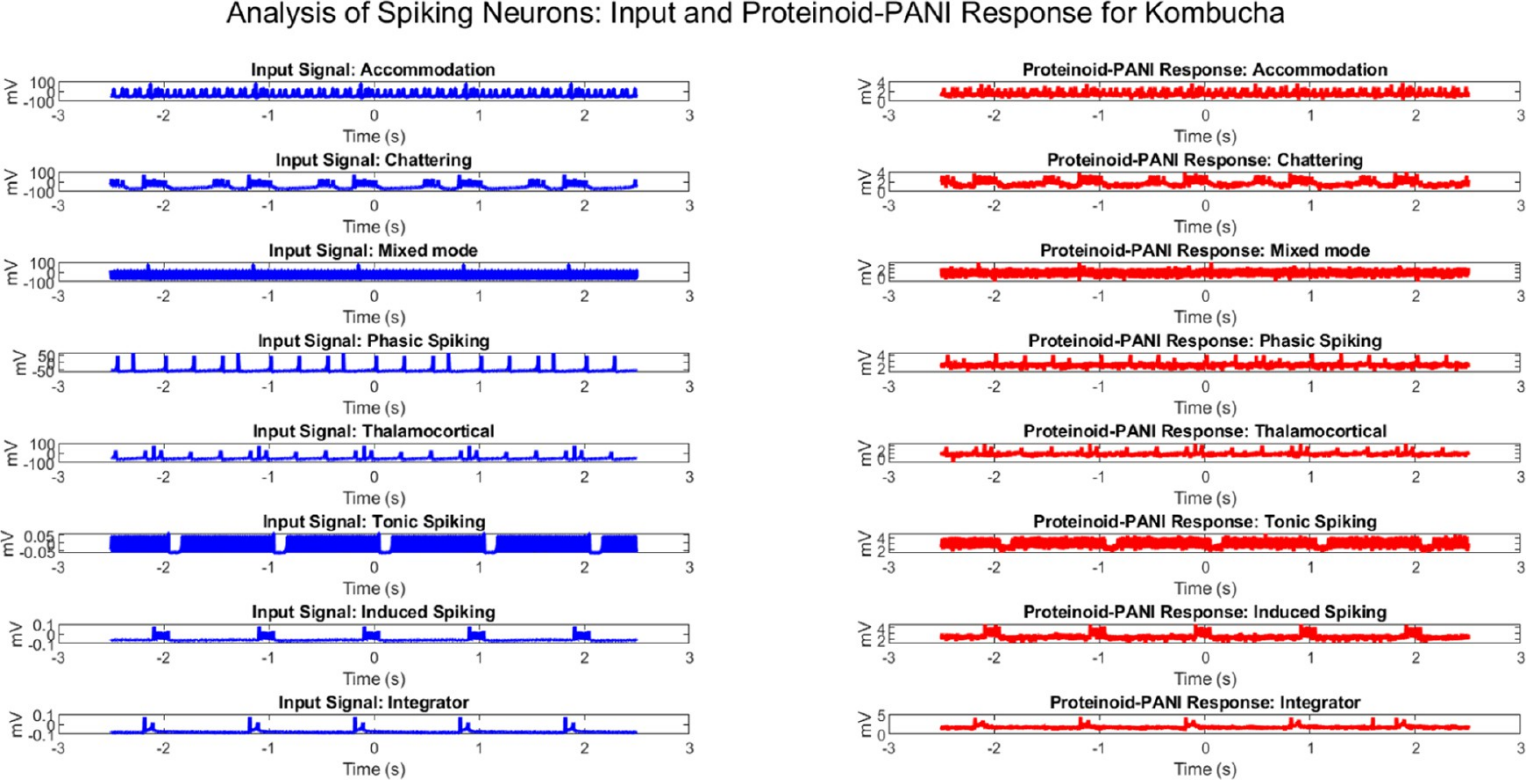
Spiking behavior of PANI–Proteinoid on the Kombucha substrate. The graphic shows the potential (mV) with time for various spiking behaviors, such as accommodation, chattering, mixed mode, phasic, thalamocortical, tonic, induced, and integrator spiking. The potential values range from −1.19 to 4.72 mV, with tonic spiking exhibiting the highest potential values (max: 4.72 mV) and mixed mode spiking showing the lowest potential values (min: −1.19 mV). The spiking patterns on the kombucha substrate appear to be more consistent and less variable compared to the other substrates, as evident from the similar potential ranges and waveforms observed across the various spiking behaviors.

**Figure 12:**
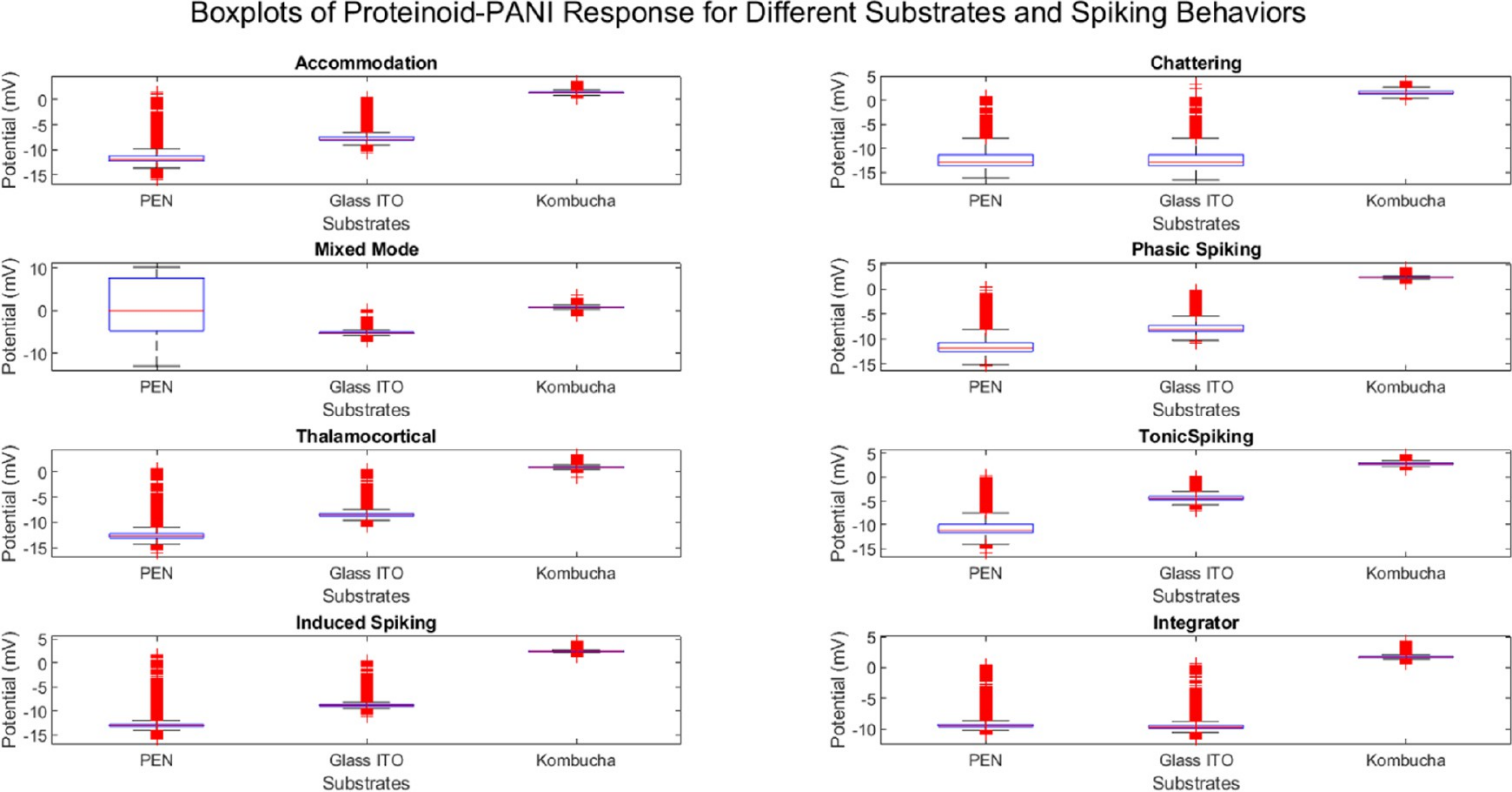
Boxplots are used to compare the potential (measured in millivolts) of PANI-proteinoid for different spiking behaviors on PEN, Glass ITO, and Kombucha substrates. Each boxplot displays the distribution of potential values for a certain pattern of spikes on a particular surface. The median potential is represented by the central line in each box, while the first and third quartiles are indicated by the box limits. The whiskers span from the lowest to the highest values, excluding any outliers. The boxplots display clear patterns of potential distribution among the substrates. The PEN and Glass ITO substrates display a broader spectrum of potential values, with more prominent negative potentials, suggesting a prevalence of inhibitory responses. Conversely, the Kombucha substrate has a more limited spectrum of potential values, with the median being closer to zero. This indicates a state of equilibrium between stimulating and inhibiting reactions. The variations in the potential distributions across the substrates emphasize the impact of substrate properties on the spiking characteristics of PANI-proteinoid networks. The Kombucha substrate exhibits more consistent and balanced potential responses compared to the PEN and Glass ITO substrates.

Further research into the fundamental mechanisms of substrate–dependent spiking behavior, such as analyzing the surface properties, conducting electrical impedance spectroscopy, and employing computational modeling, can offer more profound understanding of the observed variations and direct improvements of substrate–PANI–proteinoid interfaces for specific uses in neuromorphic computing and biosensing. Figure 11 shows the spiking behavior of PANI–Proteinoid on the kombucha substrate. The potential values range from −1.19 to 4.72 mV, with tonic spiking exhibiting the highest potential values (max: 4.72 mV) and mixed mode spiking showing the lowest potential values (min: −1.19 mV). In contrast, Figure 33 in the Supporting Information displays the spiking behavior of PANI–Proteinoid on the ITO–glass substrate. The potential levels vary from −16.54 to 3.36 mV, with chattering spiking exhibiting the widest range (min: −16.54 mV, max: 3.36 mV). The integrator spiking behavior has the lowest potential values among the spiking patterns, with a minimum of −11.73 mV. Similarly, Figure 34 in theSupporting Information illustrates the spiking behavior of PANI–Proteinoid on the PEN substrate. The potential values range from −16.14 to 10.13 mV, with mixed mode spiking displaying the highest potential values (max: 10.13 mV) and chattering spiking showing the lowest potential values (min: −16.14 mV). The integrator spiking behavior has a minimum potential value of −10.81 mV on the PEN substrate (Figure 12).

#### Firing Rate Curves for PANI–Proteinoid on Kombucha, PEN, and ITO–Glass Substrates

An analysis was conducted on the firing rate curves of PANI–Proteinoid with distinct spiking behaviors on Kombucha, PEN, and ITO–glass substrates to examine the effect of the substrate on the spiking activity. Figure 26 in the Supporting Information displays the firing rate curves for the Kombucha substrate, showing mostly negative firing rates throughout the spiking behaviors. The mixed mode spiking demonstrates a significant reduction in spiking activity, with a firing rate of −0.19 spikes/s, indicating substantial suppression. On the other hand, induced spiking exhibits a slight rise in firing rate, with a value of 0.02 spikes per second, indicating a slight increase in spiking activity. The firing rate profile of PANI–Proteinoid on the Kombucha substrate indicates that it has a suppressive effect on the spiking behavior.

The firing rate curves on the PEN substrate (Figure 27 in the Supporting Information) show a combination of positive and negative values, indicating a wide range of spiking activity. The mixed mode spiking is characterized by a markedly negative firing rate of −1.95 spikes/s, indicating a substantial reduction in spiking activity. Phasic spiking and induced spiking both show positive firing rates of 0.40 and 0.32 spikes/s, respectively. This indicates an increase in spiking activity for these particular behaviors. The spiking behavior of PANI–Proteinoid is influenced in a more diverse manner by the PEN substrate as compared to the Kombucha substrate.

Figure 28 in the Supporting Information displays the fire rate curves for the ITO–glass substrate, showing mostly positive firing rates throughout the spiking behaviors. Tonic spiking demonstrates a peak firing rate of 0.43 spikes per second, suggesting a significant increase in spiking activity. In contrast, chattering exhibits a negative firing rate of −0.32 spikes/s, indicating a decrease in spiking activity associated with this specific activity. Typically, the ITO–glass substrate enhances the spiking activity of PANI–Proteinoid, although there may be certain cases where this is not true.

The firing rate analysis demonstrates how the spiking activity in PANI–Proteinoid is influenced by the substrate. The Kombucha substrate has a general ability to reduce spiking behavior, but the PEN substrate shows a wider range of firing rates, with both suppression and increase of spiking activity depending on the specific behavior. On the other hand, the ITO–glass substrate typically encourages the occurrence of spiking activity, with tonic spiking demonstrating the most significant improvement.

**Figure 13:**
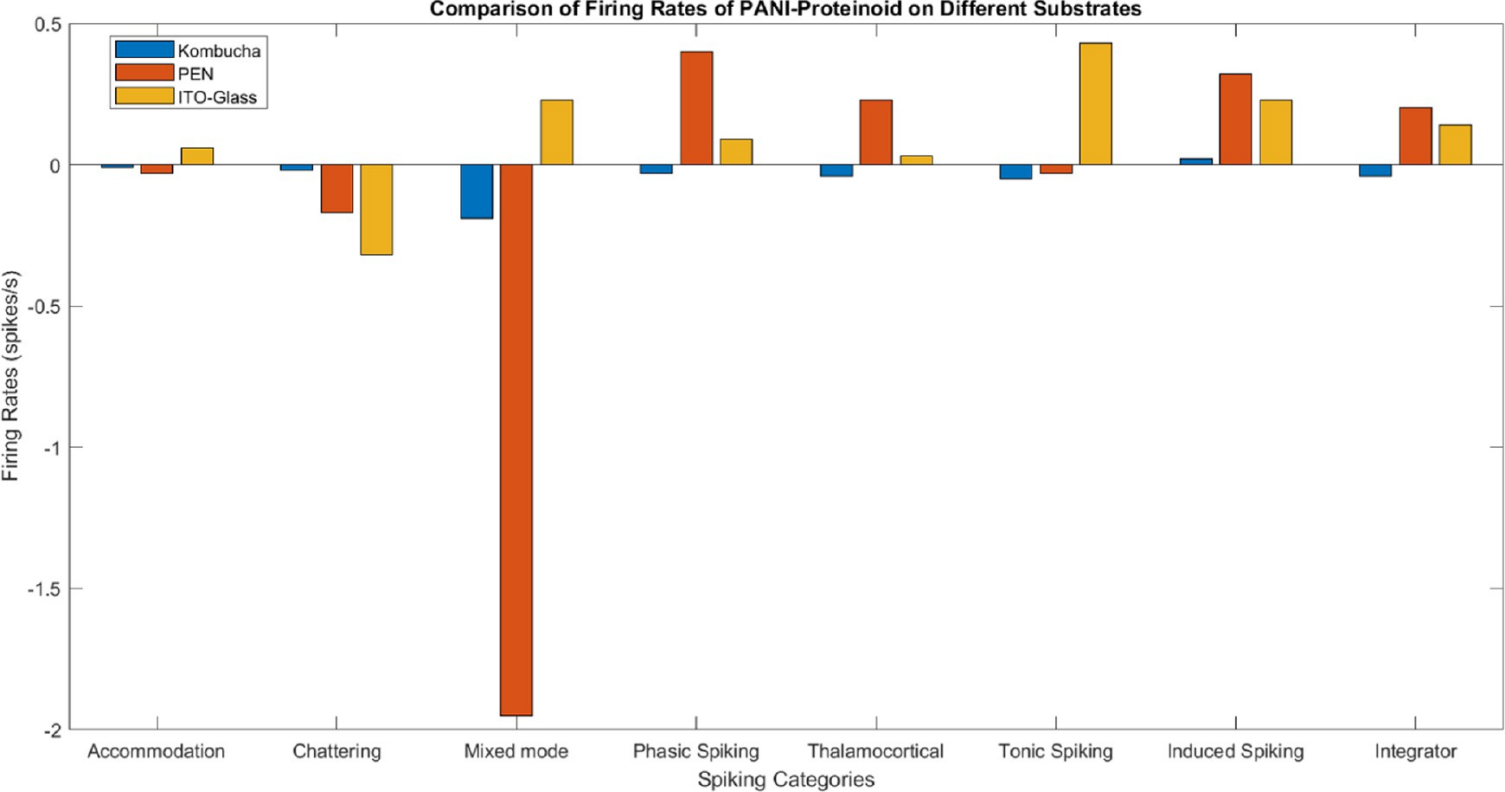
Comparison of firing rates of PANI–Proteinoid across different substrates (kombucha, PEN, ITO–Glass) for various spiking categories.

Figure 13 illustrates the comparison of fire rates of PANI–Proteinoid on different substrates (Kombucha, PEN, ITO-Glass) for distinct spiking categories. There is a noticeable variation in the firing rates among the substrates, particularly in the Mixed mode and Tonic Spiking classes. The variations emphasize how the behavior of PANI–Proteinoid in generating neuronal activity is influenced by the substrate.

These findings indicate that the substrate has a vital impact on the spiking kinetics of PANI–Proteinoid. The variations in fire rates among the substrates can be attributed to the unique surface qualities, roughness, and electrical characteristics of each substrate material. The Kombucha substrate, being a natural biopolymer, may have distinct characteristics that can prevent spiking activity, while the ITO–glass substrate’s electrical characteristics can promote increased spiking behavior.

## Discussion

This study thoroughly examined the interaction of proteinoids–polyaniline (PANI) composites and simulated neurons, with a specific focus on different spiking behaviors and their statistical features. The proteinoid–PANI samples consistently displayed lower skewness and kurtosis values compared to the input neurons across various spiking behaviors, such as thalamocortical stimulation, accommodation spikes, chattering spike, phasic spiking, induced spiking, spike integration, tonic spiking, and mixed mode spiking. These findings suggest that the proteinoid–PANI samples had spike amplitude distributions that were more balanced and less skewed than those of the input neurons. The proteinoid–PANI samples had reduced skewness values, indicating a more balanced distribution of spike amplitudes around the mean, with fewer outliers on both ends of the distribution. The stabilizing impact of the proteinoid–PANI composite on the spiking behavior can be related to its distinctive electrical and chemical features.^42^ The decreased skewness seen in the proteinoid–PANI samples indicates a more equal and uniform spiking response in comparison to the input neurons. Similarly, the proteinoid–PANI samples had reduced kurtosis values, indicating that their spike amplitude distributions had lighter tails and were less susceptible to outliers compared to the input neurons. This indicates that the proteinoid–PANI composite might play a role in controlling the occurrence of intense spike amplitudes, possibly by adjusting the excitability of the simulated neurons.^43^ The decreased kurtosis observed in the proteinoid–PANI samples indicates a more regulated and predictable pattern of spikes. In addition, the proteinoid–PANI samples consistently exhibited smaller standard deviations of spike amplitudes compared to the input neurons for all spiking behaviors. This discovery strengthens the idea that the proteinoid–PANI composite improves the stability and decreases the variability of the spiking activity. The proteinoid–PANI samples exhibit reduced standard deviations, indicating a narrower range of spike amplitudes and a more consistent spiking response. This characteristic can be advantageous for ensuring accurate information processing and signal transmission in neural interfaces.^44^ The distinct statistical aspects of the spiking behavior in the input neurons and the proteinoid–PANI samples can be linked to the unique characteristics of the proteinoid–PANI composite. The synergistic effect of the electrical conductivity of polyaniline and the compatibility with living organisms of proteinoids could potentially enhance the stabilization and control of the spiking activity.^45^ The composite material enables the efficient transmission of electrical signals while creating an appropriate microenvironment for the simulated neurons, resulting in more consistent and predictable patterns of neural activity. The findings of this work emphasize the potential of proteinoid–PANI composites as highly promising materials for neural interfaces and bioelectronic applications. The composite’s capacity to regulate and maintain the spiking behavior of simulated neurons indicates its potential to improve the effectiveness and dependability of neural recording and stimulation devices.^6^ Moreover, the decreased fluctuation and enhanced uniformity in the firing reaction of the proteinoid–PANI samples suggest that they’re suitable for applications that require accurate regulation of neuronal activity, such as in neuroprosthetics and brain–machine interfaces.^46^

**Figure 14:**
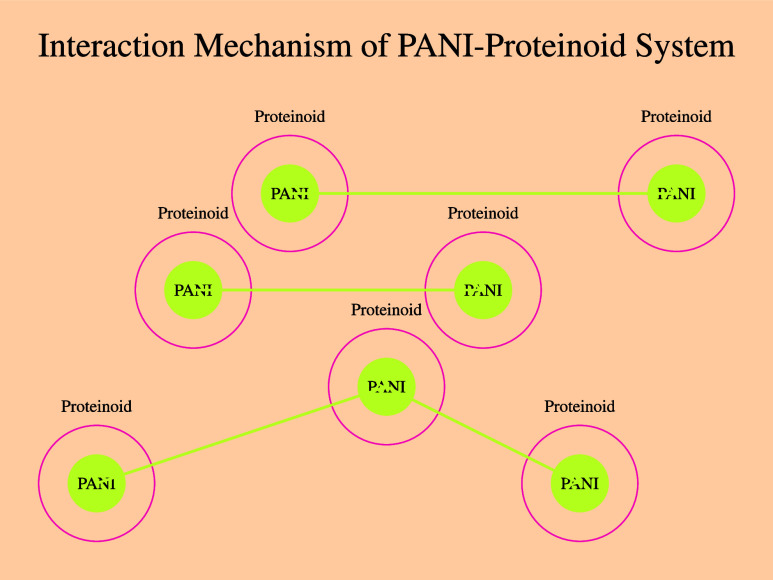
Illustration depicting the interaction mechanism of PANI nanoparticles and proteinoid microspheres. PANI nanoparticles are represented in lime color, while proteinoid microspheres are shown in magenta. The background in orange symbolizes the colloidal suspension. Arrow connections indicate the formation of a conductive network between the PANI nanoparticles.

The interaction mechanism between polyaniline (PANI) nanoparticles and proteinoid microspheres is crucial for the formation of a composite material that is both conductive and functional. The PANI nanoparticles, depicted in lime color, are enclosed within the proteinoid microspheres, displayed in magenta, resulting in a distinctive hierarchical arrangement, as depicted in Figure 14. The PANI nanoparticles are enclosed within the proteinoid microspheres by a range of methods, including electrostatic interactions, hydrogen bonding, and hydrophobic interactions. The presence of functional groups, such as amine and imine groups, on the surface of PANI nanoparticles allows them to interact with the amino acid residues of the proteinoid microspheres. These interactions promote successful integration of PANI nanoparticles into the proteinoid matrix. A significant advantage of this encapsulation technique is the improved capacity of PANI nanoparticles to disperse and remain stable in water or physiological conditions. The proteinoid microspheres serve as a barrier, inhibiting the formation of clumps and clustering of PANI nanoparticles, a frequent challenge in their use. Enhanced dispersibility facilitates a more uniform dispersion of PANI nanoparticles in the composite material, resulting in increased electrical conductivity and performance. Furthermore, the incorporation of PANI nanoparticles within the proteinoid microspheres allows for the formation of a conductive network, as seen by the arrow connections in Figure 14. The PANI nanoparticles, due to their high conductivity, can create interconnected routes inside the proteinoid matrix, which enables efficient transfer of charge and electrical communication. This conductive network replicates the neural communication observed in biological systems, where electrical signals circulate through interconnected neurons. The PANI nanoparticles within the proteinoid microspheres produce pathways that conduct electricity, similar to the axons and dendrites of neurons, enabling the transmission of electrical signals. The proteinoid microspheres function as a supporting matrix, akin to the extracellular matrix in the brain, providing a structured environment for the conductive network. The network’s electrical transmission can be used for a range of applications, including biosensors, brain interfaces, and neuromorphic computation. The PANI–proteinoid composite has the capability to identify and measure particular analytes or biomarkers in biosensing applications.^47−50^ The presence of the analyte can modify the electrical characteristics of the composite material due to the conductive network of PANI nanoparticles, allowing for sensitive and selective detection. Proteinoid microspheres can serve as a biocompatible surface for attaching biomolecules or receptors, hence improving the accuracy and selectivity of the biosensor. The PANI–proteinoid composite can facilitate direct connection between electrical devices and biological neurons in neural interfaces. The PANI nanoparticles’ conductive network enables the efficient flow of electrical signals in both directions, enabling the activation and monitoring of neural activity. Proteinoid microspheres offer enhanced biocompatibility and stability, leading to improved long–term performance and less inflammatory response in neural interface applications. Moreover, the PANI–proteinoid composite can be explored for neuromorphic computing applications, in which artificial neural networks are created to replicate the structure and functionality of biological neural networks. The PANI nanoparticles create a conductive network that can function as an artificial neural network, allowing for the processing and transmission of electrical impulses in a way that is similar to biological neurons. Proteinoid microspheres can serve as a supportive framework for artificial neural networks, enabling the design of bio–inspired computing systems.^51^ Ultimately, the process of PANI nanoparticles and proteinoid microspheres interacting entails the PANI nanoparticles being enclosed within the proteinoid matrix, resulting in the creation of a conductive network. This composite material replicates the neural connectivity observed in biological systems, facilitating effective electrical signal transmission and processing. The PANI–proteinoid composite has significant potential for diverse applications, such as biosensors, brain interfaces, and neuromorphic computing.^52^ The material’s conductive network and biocompatibility can be used for achieving advanced functionality.

The incorporation of PANI nanoparticles into proteinoid microspheres is essential for improving the characteristics of the composite in the development of advanced materials. At first, the encapsulation process occurs when PANI nanoparticles and proteinoid microspheres interact, leading to the creation of PANI nanoparticles enclosed within proteinoid microspheres. The resulting composite material displays a conductive network of PANI nanoparticles that are interconnected inside the proteinoid matrix, resulting in enhanced electrical conductivity. The improved electrical characteristic of this material allows for many uses, such as biosensing capabilities. In this context, changes in the conductivity of the composite material when it interacts with a specific substance enable the identification of biomolecules. Moreover, in the context of neural interface applications, the PANI–proteinoid composite exhibits the ability to stimulate brain activity by stimulating a network of PANI nanoparticles that are integrated into the composite. The wide range of applications highlights the promise of the PANI–proteinoid composite in many innovations in technology. In a nutshell, the key steps of the mechanism of PANI–proteinoids interaction involve:

1. Encapsulation of PANI nanoparticles within proteinoid microspheres:
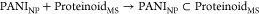
8where PANI_NP_ represents PANI nanoparticles, Proteinoid_MS_ represents proteinoid microspheres, and PANI_NP_ ⊂ Proteinoid_MS_ denotes the encapsulation of PANI nanoparticles within the proteinoid microspheres.
2. Formation of conductive network within the composite material:
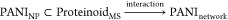
9where PANI_network_ represents the conductive network formed by the interconnected PANI nanoparticles within the proteinoid matrix.
3. Electrical conductivity of the PANI-proteinoid composite:
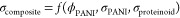
10where σ_composite_ is the electrical conductivity of the PANI–proteinoid composite, ϕ_PANI_ is the volume fraction of PANI nanoparticles, σ_PANI_ is the electrical conductivity of PANI nanoparticles, and σ_proteinoid_ is the electrical conductivity of proteinoid microspheres.
4. Charge transfer in the conductive network:
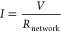
11where *I* is the electrical current, *V* is the applied voltage, and *R*_network_ is the resistance of the conductive network formed by PANI nanoparticles.
5. Biosensing application of the PANI–proteinoid composite:
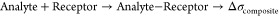
12where Analyte represents the target biomolecule, Receptor represents the immobilized receptor on the PANI–proteinoid composite, Analyte–Receptor represents the binding event, and Δ*σ*_composite_ represents the change in electrical conductivity of the composite material upon analyte binding.
6. Neural interface application of the PANI–proteinoid composite:
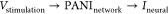
13where *V*_stimulation_ represents the applied electrical stimulation, PANI_network_ represents the conductive network of PANI nanoparticles, and *I*_neural_ represents the induced neural activity.

The experimental data, presented in Table 2, demonstrate the distinct spiking behavior of PANI–proteinoid composites on different substrates. The PEN substrate displayed a broad spectrum of potential values, ranging from −16.14 to 10.13 mV, with mixed mode spiking exhibiting the highest potential values and chattering spiking showing the lowest potential values. Similarly, the ITO substrate exhibited potential values ranging from −16.54 to 3.36 mV, with chattering spiking having the widest range and integrator spiking displaying the lowest potential values. In contrast, the kombucha substrate showed a narrower range of potential values, ranging from −1.19 to 4.72 mV, for most spiking behaviors. Tonic spiking exhibited the highest potential values, while mixed mode spiking showed the lowest potential values on the kombucha substrate. Additionally, the spiking patterns on the kombucha substrate appeared to be more consistent and less variable compared to those on the PEN and ITO substrates. The observations indicate that the selection of substrate is essential in replicating distinct spiking behaviors. The PEN and ITO substrates can be used to explore a wide variety of spiking patterns with significant variability in potential values and waveforms. On the other hand, the kombucha substrate may be better suited for investigating spiking dynamics that are more consistent and have less variability across different spiking behaviors (Table 2). Moreover, the prospective consequences outlined in Table 3 highlight the need for additional research and comparative analyses to fully understand the impact of substrate characteristics on the spiking behavior of PANI–proteinoid composites. These findings establish the basis for future study focused on maximizing substrate selection and investigating the distinct properties of each material.

**Table 3:** Comparison of Substrates (Kombucha, PEN, ITO–Glass) for Simulating Izhikevich Neurons Using PANI–Proteinoid Composites

substrate	observations	potential implications
PEN (polyethylene naphthalate)	•Wide range of potential values (−16.14 to 10.13 mV) for all spiking behaviors	•Suitable for simulating a diverse range of spiking behaviors
	•Mixed mode spiking exhibits the highest potential values (max: 10.13 mV)	•May require optimization for specific spiking patterns
	•Chattering spiking shows the lowest potential values (min: −16.14 mV)	•Potential for studying the effect of substrate properties on spiking dynamics
	•Significant variation in spiking patterns with distinct potential ranges and waveforms for each behavior	
ITO (indium tin oxide) glass	•Wide range of potential values (−16.54 to 3.36 mV) for all spiking behaviors	•Suitable for simulating a diverse range of spiking behaviors
	•Chattering spiking exhibits the widest range (min: –16.54 mV, max: 3.36 mV)	•May require optimization for specific spiking patterns
	•Integrator spiking behavior has the lowest potential values (min: –11.73 mV)	•Potential for studying the effect of substrate properties on spiking dynamics
	•More variable spiking patterns compared to kombucha substrate	•Comparative studies with other substrates to understand the influence of surface properties
	•Observable changes in potential ranges and waveforms among spiking behaviors	
Kombucha (Bacterial cellulose)	•Narrower range of potential values (−1.19 to 4.72 mV) for most spiking behaviors	•Suitable for simulating specific spiking behaviors with consistent patterns
	•Tonic spiking exhibits the highest potential values (max: 4.72 mV)	•Potential for studying the unique properties of bacterial cellulose on spiking dynamics
	•Mixed mode spiking shows the lowest potential values (min: –1.19 mV)	•May require further investigation to understand the variability in potential values for different spiking behaviors
	•More consistent and less variable spiking patterns compared to other substrates	•Comparative studies with other substrates to explore the influence of biocompatibility and porosity
	•Similar potential ranges and waveforms observed across spiking behaviors	

**Figure 15:**
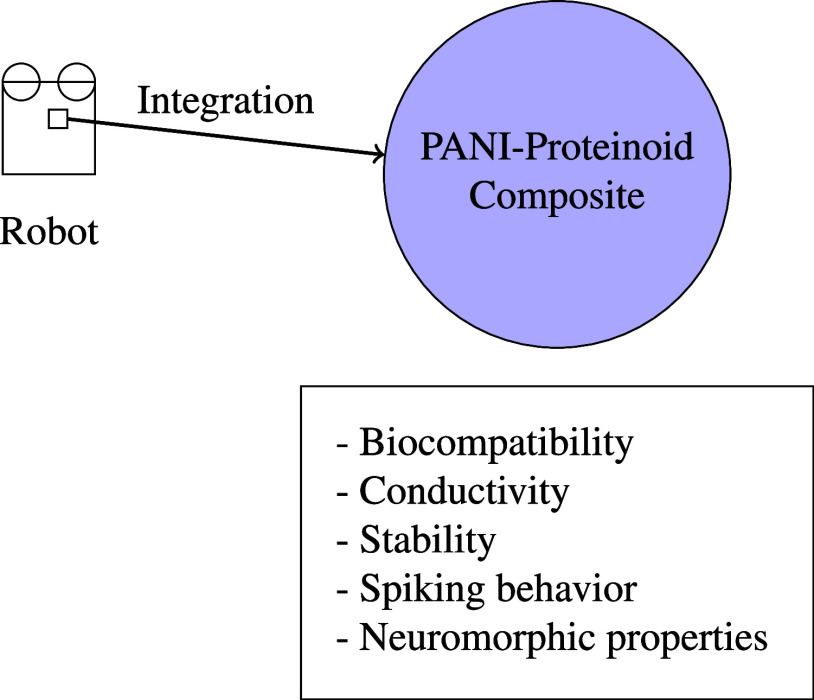
Schematic representation of the integration of PANI-proteinoid composite in robotic systems. The PANI–proteinoid composite exhibits properties such as biocompatibility, conductivity, stability, spiking behavior, and neuromorphic properties, making it suitable for enhancing the functionality and performance of robots.

The use of PANI–proteinoid composites into robotic systems has significant potential for improving their functionality and performance. The PANI–proteinoid composite, depicted in Figure 15, has several advantages such as biocompatibility, conductivity, stability, spiking behavior, and neuromorphic features. The distinctive attributes of PANI–proteinoid composites make them very suitable for use in neuromorphic robotics. Fast and accurate communication between components is crucial for neuromorphic robots that depend on spiking neural networks. The PANI–proteinoid composites possess high conductivity, which allows for the integration of intricate brain structures and permits the immediate processing of sensory information. By incorporating PANI–proteinoid composites into robotic systems, as shown in Figure 15, we can explore novel opportunities for developing intelligent and self–governing robots. Researchers can enhance the sensory perception, energy consumption, and performance of robots in dynamic environments by using the distinctive features of these composites. Nevertheless, it is crucial to recognize that the incorporation of PANI–proteinoid composites into robotic systems also poses difficulties. Additional study is required to enhance the fabrication methods, devise effective interface strategies, and tackle concerns regarding scalability and long–term stability.

Nevertheless, it is crucial to recognize the limitations of this study and the need for additional research. The present study concentrated on simulated neurons, which offer a simplified depiction of neural activity. Further research should investigate the interaction between proteinoid–PANI composites and biological neurons in order to confirm the results and evaluate the compatibility and durability of the composite material in a realistic biological setting. Furthermore, the particular mechanisms responsible for the reported implications of the proteinoid–PANI composite on the spiking behavior need to be further clarified using experimental and theoretical methods. Finally, the results emphasize the potential of proteinoid–PANI composites as very promising materials for brain interfaces and bioelectronic applications. These materials have the ability to enhance performance, dependability, and regulation of neural activity. Additional investigation is required to examine the fundamental processes, compatibility with living organisms, and durability over an extended period of time of these combinations in biological systems, thereby facilitating their effective incorporation into sophisticated neurological technologies.

**Figure 16:**
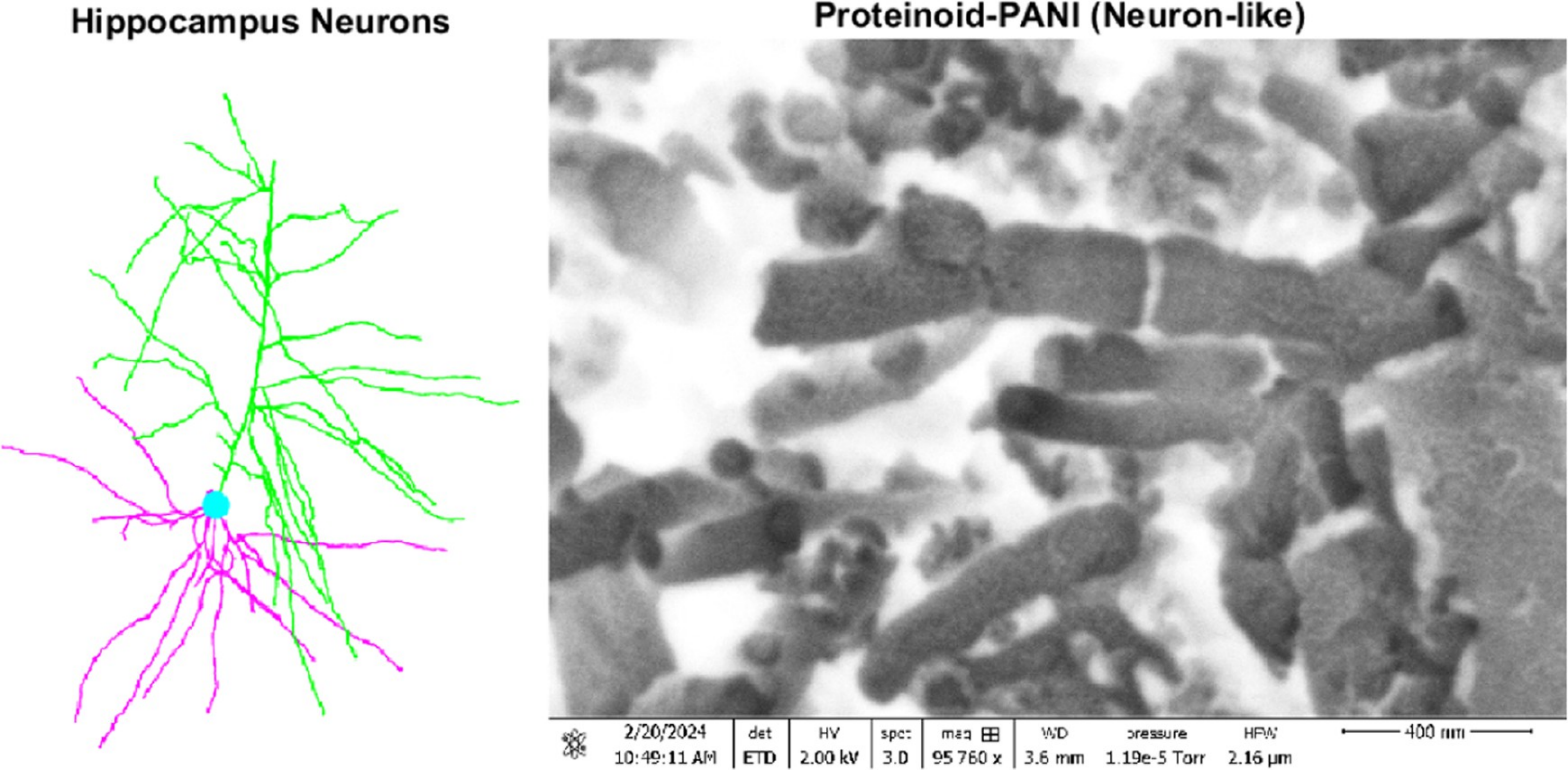
Comparison of hippocampal CA1 pyramidal neurons and proteinoid–PANI nanofibers. An image showing the complicated branching pattern and three–dimensional organization of the complex dendritic structure of CA1 pyramidal neurons in the human hippocampus. The neuron exhibits a total length of 6777.28 μm, a total surface area of 25688.7 μm^2^, and a total volume of 11497.1 μm^3^. (Right) Scanning electron microscopy (SEM) image of proteinoid–PANI nanofibers, shows an extensively interconnected network of nanofibers that resembles the intricate dendritic structure of hippocampal neurons. Proteinoid–PANI nanofibers exhibit a comparable degree of structural complexity to living neurons,characterized by a large number of branches and a three–dimensional configuration reminiscent of biological neurons. Scale bar: 400 nm.

Is it possible to replicate the behavior of human neurons using proteinoid–PANI nanofibers? The remarkable resemblances observed in the morphology and structure of proteinoid–PANI nanofibers suggest their potential as a biomimetic material. This similarity with different types of human neurons indicates promising applications in neuromorphic computing and brain–inspired electronics. Figure 16 demonstrates the striking analogy between the proteinoid–PANI nanofiber network and the complex dendritic structure of hippocampal CA1 pyramidal neurons in the human brain. The CA1 pyramidal neurons have a highly intricate and complex structure, with multiple stems, bifurcations, and branches.^53^ The proteinoid–PANI nanofibers exhibit an intricate network and minute dimensions that closely resemble the complex architecture of dendrites. The similarities extend beyond visual appearance, as the quantitative analysis of the CA1 pyramidal neurons shows a total length of 6777.28 μm, a total surface area of 25688.7 μm^2^, and a total volume of 11497.1 μm^3^. The measurements exhibit a level of structural complexity that is similar to the dimensions and network complexity observed in the proteinoid–PANI nanofiber system. In addition, the proteinoid–PANI nanofibers exhibit similar morphological characteristics to certain human neurons, like cerebellar Purkinje neurons.^54^ These neurons are known for their intricate dendritic tree structure and extensive branching. The complex web of PANI nanofibers closely mirrors the complex architecture of Purkinje neurons, indicating that the material has the potential to emulate their behavior and functionality. This discovery holds great potential for the development of neuromorphic computing and brain–inspired electronics, which is truly exciting. Through harnessing the analogies between these bio–inspired materials and biological neurons, scientists can explore cutting–edge computational models, explore into neuronal dynamics, and design innovative neural interfaces. It is important to note that the proteinoid–PANI nanofibers have striking similarities to human neurons, but it is crucial to remember that they are an artificial system. Additional research is required to gain an in-depth knowledge of the functional capabilities and limitations of this material in emulating neuronal behavior and processing. The interesting similarities between the morphology and structure of proteinoid–PANI nanofibers and different types of human neurons, as emphasized in Table 4, indicate that this biomimetic material has the capacity to function as a suitable analogue for neuromorphic computing and brain–inspired electronics. Table 4 presents a thorough comparison of several types of neurons, such as cerebellar Purkinje neurons, hippocampal neurons, pyramidal neurons, and dentate gyrus neurons, along with their distinct activities. The table also demonstrates the capacity of proteinoid–PANI nanofibers to imitate the structural and operational characteristics of these neurons. For example, the detailed nanofiber structure of PANI closely reflects the complex branching pattern of cerebellar Purkinje neurons, which are essential for motor coordination, balance, and precise regulation of movements. Likewise, the interlinked network of PANI nanofibers can mirror the dendritic branching patterns of hippocampal neurons, which play a role in cognitive processes such as learning, memory formation, and spatial navigation. As indicated in Table 4, this similarity in structure enables research to examine the connection between dendritic structure and memory formation, as well as explore the impact of synaptic plasticity on learning and the storage of information. Moreover, the configuration of PANI nanofibers can mimic the compact arrangement and structured dendritic morphology of dentate gyrus neurons, which play a role in pattern discrimination, memory development, and the generation of new neurons.

**Table 4:** Comparison of Neuron Types and the Potential of Proteinoid–PANI Nanofibers for Biological Mimicry

neuron type	function	proteinoid–PANI mimicry potential
Cerebellar Purkinje Neurons^55^	•Play a crucial role in motor coordination, balance, and fine motor control	•Intricate nanofiber network of PANI can mimic the complex dendritic branching of Purkinje neurons
	•Integrate and process information from multiple inputs	•Potential to model information processing and integration in the cerebellum
	•Exhibit complex dendritic arborization for extensive synaptic connectivity	•Explore the role of dendritic complexity in motor coordination and control
Hippocampal Neurons^56^	•Involved in learning, memory formation, and spatial navigation	•Interconnected network of PANI nanofibers can resemble the dendritic branching of hippocampal neurons
	•Exhibit distinct dendritic branching patterns for efficient synaptic transmission	•Study the relationship between dendritic structure and memory formation
	•Play a key role in the formation and retrieval of episodic memories	•Investigate the role of synaptic plasticity in learning and information storage
Pyramidal Neurons^57−59^	•Found in the cerebral cortex and play a role in cognitive functions, such as perception, attention, and decision–making	•Structural features of PANI nanofibers can be compared to the soma and dendritic organization of pyramidal neurons
	•Possess a characteristic soma shape and apical dendrites for receiving and integrating synaptic inputs	•Explore the relationship between neuronal morphology and cognitive functions
	•Involved in the formation of cortical circuits and information processing	•Model the integration of synaptic inputs and the generation of output signals in cortical circuits
Dentate Gyrus Neurons^60,61^	•Play a critical role in pattern separation and the formation of new memories	•Arrangement of PANI nanofibers can resemble the dense packing and organized dendritic structure of dentate gyrus neurons
	•Exhibit densely packed somas and highly organized dendritic arborizations	•Investigate the role of neuronal density and connectivity in pattern separation and memory formation
	•Involved in the process of neurogenesis and the integration of new neurons into existing circuits	•Explore the potential of PANI nanofibers to model neurogenesis and the integration of new neurons

## Conclusions

To summarize, this study has shown the remarkable properties of proteinoids–PANI composites as bio–inspired materials for neuromorphic computing. Due to their capacity to display various spiking behaviors, improved stability, and consistent electrical activity, they are very attractive candidates for simulating biological neural networks to build sophisticated computing systems. The selection of substrate is a critical factor in determining the spiking kinetics of proteinoids–PANI composites. The kombucha substrate exhibits a limited range of potential values for most spiking behaviors and extremely low potential values for tonic, induced, and integrator spiking. This makes it well–suited for replicating certain spiking behaviors with consistent patterns. The distinctive feature of the kombucha substrate can be attributed to its biocompatibility, large surface area, and porosity, which could affect the interaction between proteinoids–PANI composites and stimulated neurons. Conversely, the ITO–glass and PEN substrates display a broad spectrum of potential values for all spiking behaviors, suggesting their capacity to investigate a wide variety of spiking patterns. The ITO–glass substrate’s exceptional electrical conductivity, remarkable transparency, and excellent surface smoothness likely enable it to facilitate diverse spiking dynamics. Moreover, the flexibility, lightweight characteristics, and cost–effectiveness of the PEN substrate make it a highly appealing choice for conducting large–scale processing and studying the impact of substrate features on spiking behavior. The insight gained from this research adds to the expanding domain of neuromorphic engineering and facilitates the progress of brain–inspired technology in the future. By using the distinctive characteristics of proteinoids–PANI composites and further investigating their incorporation with simulated and biological neurons, we can unveil novel prospects for highly efficient, adaptable, and intelligent computing systems that have the potential to revolutionize diverse fields such as robotics, computer science, and neuroscience.

## Data Availability

This data is accessible via the online database Zenodo (https://zenodo.org/records/10949539).

## Supporting Information Available

Appendix, additional content (PDF)
