# Supplementary material for: Proteinoids–Polyaniline Interaction with Stimulated Neurons on Living and Plastic Surfaces
Source: ACS Omega. 2024 Nov 5;9(46):45789–810. doi: 10.1021/acsomega.4c03546 (PMC11579727; doi:10.1021/acsomega.4c03546)
Supplement: Supplementary file 1 — ao4c03546_si_001.pdf [file ao4c03546_si_001.pdf]

Appendix

Statistical Analysis of Spiking Activity in Proteinoid–PANI Samples without Surface Interaction

Thalamo Cortical Spiking

Statistically, the proteinoid–PANI response and the input neurons exhibit distinct characteristics in response to thalamocortical stimulation of the polyaniline–proteinoid sample. Key statistics comparing the input neurons and the proteinoid–PANI sample are detailed in Table 9. The input neurons demonstrate greater skewness (4.43 mV) and kurtosis (34.19 mV) values in comparison to the sample containing proteinoid–PANI, which possesses skewness of 3.01 mV and kurtosis of 18.83 mV. The observed distinctions indicate that the proteinoid–PANI sample exhibits a less elevated and more symmetrical distribution of activity in response to thalamocortical stimulation in comparison to the simulated neurons.

In addition, the statistical data presented in Table 9 illustrates the distinctions between the input neurons and the proteinoid–PANI sample in terms of the mean, maximum, minimum, and standard deviation. The mean negative potential of the input neurons is -59.92 mV, with a maximum value of 73.13 mV and a minimum value of -71.94 mV. On the other hand, the proteinoid–PANI sample demonstrates an average positive potential of 2.74 mV, ranging from a peak of 7.37 mV to a trough of 0.95 mV. The aforementioned values suggest that the proteinoid–PANI sample exhibits a restricted range and a higher average potential in response to thalamocortical stimulation in comparison to the input neurons.

Upon thalamocortical stimulation, the proteinoid–PANI sample exhibited spiking activity, as depicted in Figure 17. The graph illustrates clear spiking patterns characterised by fluctuating inter–spike intervals and amplitudes. This indicates that the proteinoid–PANI sample is sensitive to the input stimulation with dynamic and complex responses.

Figure 31 illustrates a box plot comparison of the potentials of the input neurons and the proteinoid–PANI sample in order to further examine the distinctions between the two. The box plot presents a graphical representation of the range and distribution of potentials for the proteinoid–PANI sample as well as the input neurons. When compared to the proteinoid–PANI sample, the input/simulated neurons demonstrate a greater diversity of potentials, including a greater interquartile range and more extreme outliers. This observation is consistent with the higher standard deviation of the input neurons (11.47 mV) in Table 9, which contrasts with the proteinoid–PANI sample (0.45 mV).

The findings of this study indicate that the proteinoid–PANI sample exhibits unique statistical characteristics and spiking patterns in response to thalamocortical stimulation, in contrast to the input/simulated neurons. The proteinoid–PANI sample potentially demonstrates emergent properties and complex dynamics, as proposed by the observed differences in skewness, kurtosis, mean, maximum, minimum, and standard deviation, which indicate that it processes and transforms the input stimulation in a distinctive fashion.

‘

Table 5. Comparison of statistical properties between input neurons and proteinoid–PANI samples under thalamocortical stimulation. The proteinoid–PANI samples display reduced skewness (3.01) and kurtosis (18.83) in comparison to the input neurons (4.43 and 34.19, respectively), suggesting a distribution of activity that is more symmetrical and less peaked. The sample data indicate that the proteinoid–PANI samples exhibit a greater average potential (2.74 mV) and a smaller range (maximum: 7.37 mV, minimum: 0.95 mV) in comparison to the input neurons (average: -59.92 mV, maximum: 73.13 mV, minimum: -71.94 mV). The proteinoid–PANI samples exhibit a significantly lower standard deviation (0.45) compared to the input neurons (11.47), indicating a more consistent and less variable response to thalamocortical stimulation. The distinctions emphasise the distinct signal processing and transformation properties of the proteinoid–PANI system.

Statistics	Input	Proteinoid-PANI
Skewness	4.43	3.01
Kurtosis	34.19	18.83

Sample Statistics	Mean	Max	Min	Std
Input	−59.92	73.13	−71.94	11.47
Proteinoid-PANI	2.74	7.37	0.95	0.45

Spike Accommodation

Table 9 presents a comparison of the statistical characteristics of the input accumulation spike neurons and the proteinoid–PANI samples, highlighting differences in their spiking patterns. Both the input neurons and the proteinoid–PANI samples display non–Gaussian distributions of spike amplitudes, as indicated by the skewness and kurtosis values. Nevertheless, the proteinoid–PANI samples exhibit reduced skewness (2.08 mV) and kurtosis (8.60 mV) in comparison to the input neurons (2.66 mV and

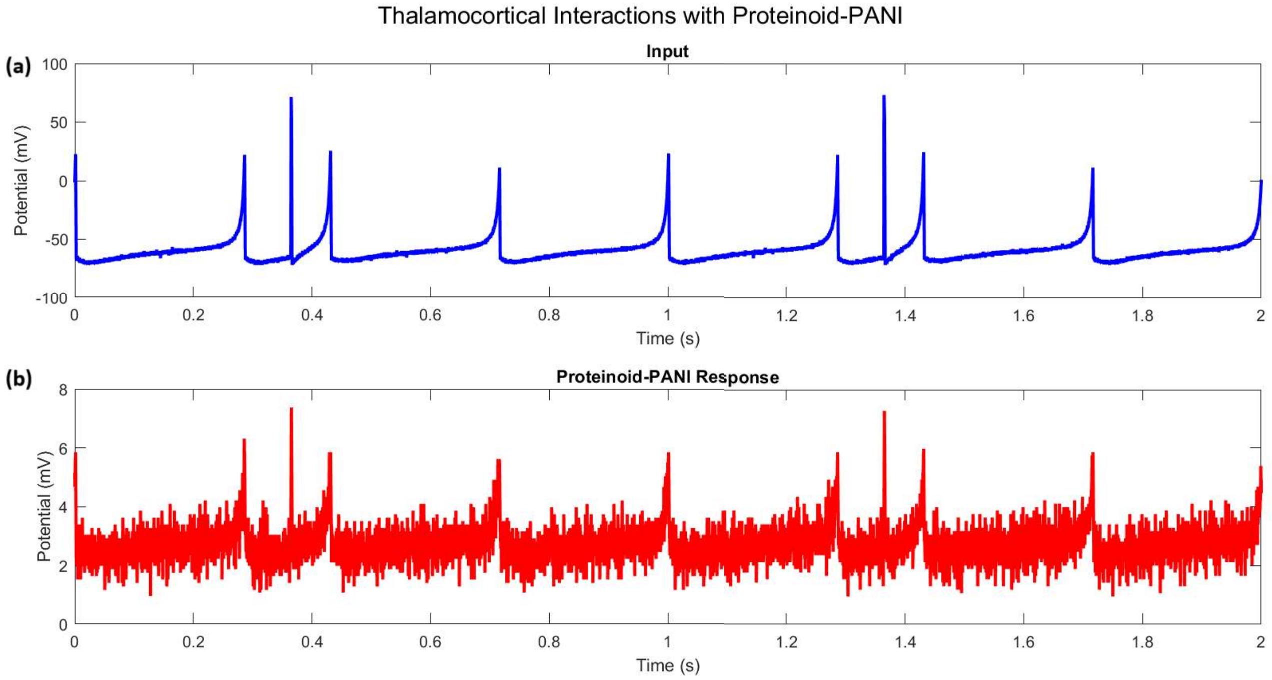

Figure 17. A comparison between simulated thalamocortical input and proteinoid–PANI output response. (a) A simulated thalamocortical input spike train that depicts the activity of input neurons. The input has a complex temporal pattern, with changing interspike intervals and amplitudes. (b) The response of proteinoid–PANI samples to thalamocortical input. The proteinoid–PANI system produces different spiking patterns, with more regular and consistent interspike intervals than the input. The output response also has a larger mean potential and a tighter range of amplitudes, showing signal transformation and stabilisation properties in the proteinoid–PANI system. The differences in temporal dynamics and statistical features between the input and output demonstrate the proteinoid–PANI samples' distinct information processing capabilities, implying their potential as bio–inspired computational elements.

11.94 mV, respectively). These findings indicate that the proteinoid–PANI samples exhibit a more balanced distribution of spike amplitudes and fewer cases of extreme values, in contrast to the simulated/input neurons of the accomodation.

The sample statistics provide more evidence of the differences between the input neurons and the proteinoid–PANI samples. The input neurons have a lower average potential of –48.46 mV and a broader spectrum of voltage values, ranging from a minimum of –71.94 mV to a maximum of 72.55 mV. On the other hand, the proteinoid–PANI samples demonstrate a greater average potential of 2.80 mV and a smaller range, with a minimum of 0.83 mV to a maximum of 6.79 mV. The proteinoid–PANI samples have a considerably lower standard deviation (0.57 mV) compared to the input neurons (15.23 mV), suggesting a more consistent and less unpredictable spiking behaviour.

Figure 18 presents a graphical depiction of the spiking behaviour of the simulated accommodation input neurons and the proteinoid–PANI samples. The input neurons display a complex and erratic spiking pattern, characterised by fluctuating amplitudes and intervals between spikes. On the other hand, the proteinoid–PANI samples exhibit a more regular and consistent pattern of spikes, characterised by homogeneous amplitudes and shorter intervals between spikes. These findings indicate that the proteinoid–PANI system exhibits a greater capacity for spike accommodation, adjusting its spiking behaviour to ensure a more consistent and predictable output.

The boxplots in Figure 31 provide a visual representation of the variations in the potential distributions between the simulated neurons and the proteinoid–PANI samples. The input neurons exhibit a broader distribution, as seen by a higher interquartile range and more variability in their potential levels. In contrast, the proteinoid–PANI samples have a narrower distribution and a reduced number of outliers, indicating a more concentrated and consistent range of potential values. This provides evidence that the proteinoid–PANI system has enhanced spike accommodation, hence controlling its capacity to sustain a more regular output.

The spike accommodation seen in the proteinoid–PANI samples can be attributed to the inherent characteristics of the material and its distinct structure. The integration of conductive polyaniline and self–assembled proteinoid nanostructures forms an intricate network capable of regulating and maintaining stable spiking behaviour. The proteinoid nanostructures can

serve as a storage system for electric charge, allowing for controlled spiking activity and preventing excessive oscillates in potential.

Furthermore, the increase in spike accommodation observed in the proteinoid–PANI system may be attributed to its capacity to adjust and react to external stimuli. The material's ability to respond to mechanical pressure and other environmental factors allows it to adapt its spiking behaviour in order to maintain a consistent output in different settings. The proteinoid–PANI system's versatility and resilience make it a highly intriguing choice for biosensing applications, where the ability to reliably and consistently produce spikes is of utmost importance.

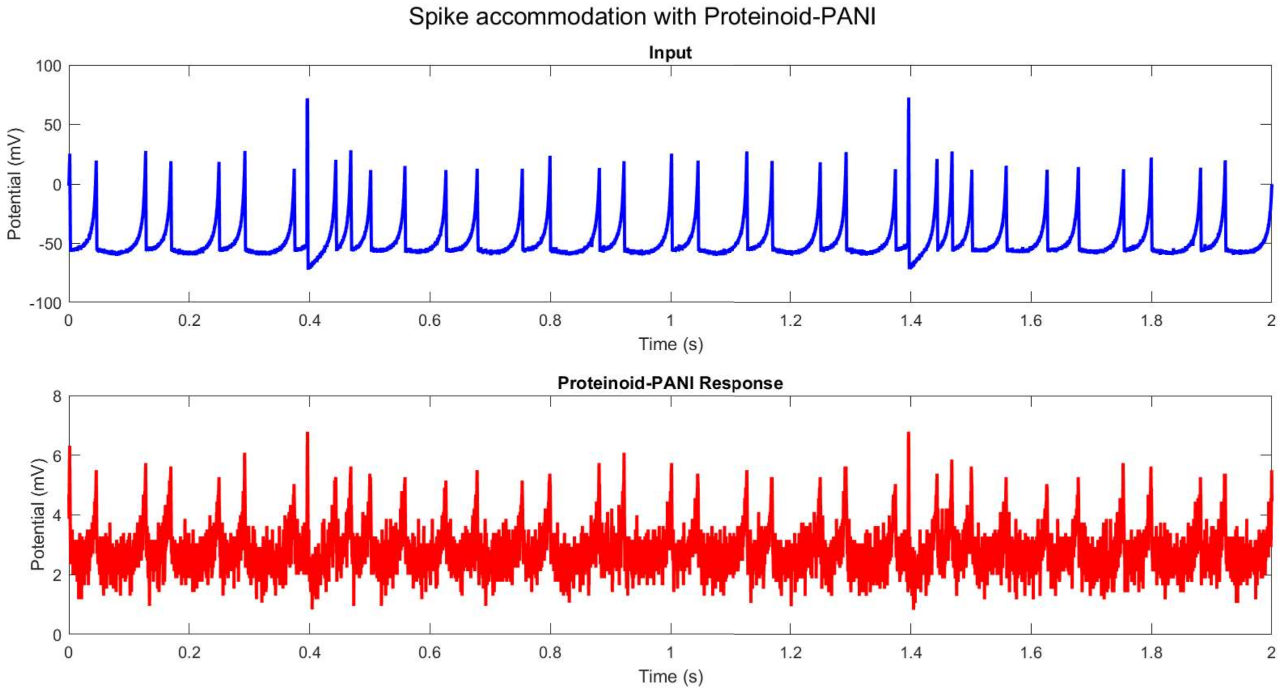

Figure 18. A comparison of proteinoid–PANI samples and simulated input neurons (accommodation spikes) in terms of spiking activity. Depending in amplitude and inter–spike interval, the input neurons display an irregular and complex spiking pattern. On the other hand, the proteinoid–PANI samples exhibit a spiking pattern that is noticeably more regular and consistent, characterised by amplitudes that are relatively consistent and inter–spike intervals that are fairly transient. This implies that the proteinoid–PANI system possesses an increased capacity for accommodating spikes, thereby modifying its spiking behaviour in order to preserve a more consistent and predicted output.

Spike Chattering

The spiking behavior of PANI–proteinoid samples was investigated and compared to the chattering spikes observed in the input neurons. Table 9 displays the statistical comparison between the input neurons and the PANI–proteinoid samples. The skewness and kurtosis values offer information about the distribution of the spike amplitudes. The input neurons demonstrate a greater skewness of 1.72 mV in contrast to the PANI–proteinoid samples, which possess a skewness of 1.24 mV. This suggests that the amplitudes of the spikes from the input neurons are distributed in a way that is not symmetrical, with a greater number of spikes having higher values. Comparatively, the kurtosis value of 6.90 mV for the input neurons exceeds the kurtosis of 4.60 for the PANI–proteinoid samples, indicating that the input neurons exhibit a distribution that is more sharply peaked with heavier tails.

Figure 19 illustrates the chattering spikes observed in the input neurons and the related response of the PANI-proteinoid samples. The input neurons exhibit rapid and irregular patterns of spiking, characterised by fluctuations in both amplitude and inter–spike intervals. In contrast, the PANI–proteinoid samples demonstrate a more uniform and predictable pattern of spikes, characterised by lower fluctuations and a narrower range of intensities.

The box plots depicted in Figure 31 provide a visual representation of the disparity in the distribution of spike amplitudes between the input neurons and the PANI–proteinoid samples. The input neurons have a broader distribution, which signifies a greater range between the first and third quartiles and a higher degree of variability in the spike amplitudes. The PANI–proteinoid samples have a narrower distribution and a reduced number of outliers, indicating a more concentrated and consistent range of spike amplitudes.

Table 6. A comparison of the statistical properties of proteinoid–PANI samples and input neurons (accommodation spikes). Indicating a more symmetrical distribution of spike amplitudes with fewer extreme values, the proteinoid–PANI samples display reduced values of skewness and kurtosis. In contrast to the input neurons, the proteinoid–PANI samples exhibit a narrower range of values and a higher mean potential, as well as a substantially reduced standard deviation. These results indicate that the proteinoid–PANI samples exhibited spiking behaviour that was more consistent and less variable.

Statistics	Input	Proteinoid-PANI
Skewness	2.66	2.08
Kurtosis	11.94	8.60

Sample Statistics	Mean	Max	Min	Std
Input	−48.46	72.55	−71.94	15.23
Proteinoid–PANI	2.80	6.79	0.83	0.57

Table 7. Statistical comparison of chattering spike behavior between input neurons and proteinoid–PANI samples. Input neurons had a more asymmetric and peaked spike amplitude distribution than proteinoid–PANI samples (skewness: 1.24 mV, kurtosis: 4.60 mV). The input neurons exhibit a lower mean spike amplitude (–56.73 mV) and a broader range (–77.10 mV to 72.55 mV) than proteinoid–PANI samples (mean: 2.37 mV, range: 0.24 mV to 6.90 mV). The spike amplitudes of input neurons are more variable than those of proteinoid–PANI samples (0.78 mV), since their standard deviation (19.85 mV) is much higher. These measurements show that proteinoid–PANI samples have a more stable and consistent chattering spike behaviour than input neurons, with a more symmetric distribution, shorter range, and lower spike amplitude variability.

Statistics	Input	Proteinoid–PANI
Skewness	1.72	1.24
Kurtosis	6.90	4.60

Sample Statistics	Mean	Max	Min	Std
Input	–56.73	72.55	–77.10	19.85
Proteinoid–PANI	2.37	6.90	0.24	0.78

The variations in spiking activity and statistical features between input neurons and PANI–proteinoid samples show that the system stabilises chattering spikes. The PANI–proteinoid samples spike more regularly and consistently than the input neurons, with less spike amplitude fluctuation. The PANI–proteinoid system's unique conductivity and charge transport paths may help regulate and smooth chattering spikes.

The capacity to control and stabilise spiking behaviour is vital for neuromorphic computing and signal processing applications, and PANI–proteinoid shows promise in these areas. The reduced chattering and better consistency of PANI–proteinoid spiking patterns suggest that this material could reduce irregular and noisy spiking activity in artificial neural networks and bio–inspired computing systems.

Phasic Spiking

The phasic spiking behavior of the PANI–proteinoid sample was analyzed and compared to the input neurons. Table 8 presents the statistical comparison between the input neurons and the PANI–proteinoid sample for phasic spiking.

Both kurtosis and skewness measurements offer valuable information about the distribution of the spike amplitudes. The input neurons demonstrate a larger skewness value of 2.05 mV, in contrast to the PANI–proteinoid sample, which has a skewness value of 1.84 mV. This suggests that the amplitudes of the spikes from the input neurons are distributed in a way that is not symmetrical, with a longer tail towards higher values. Comparatively, the kurtosis value of 7.55 mV for the input neurons exceeds the kurtosis of 6.67 mV for the PANI–proteinoid sample, indicating that the input neurons have a distribution that is more sharply peaked with heavier tails.

The sample statistics (Fig. 31) provide more evidence of the differences between the input/phasic spiking neurons and the PANI–proteinoid sample. The mean value of the input neurons is –45.83 mV, with a wide range of values from –71.94 mV to 62.23 mV. On the other hand, the PANI–proteinoid sample shows a greater average value of 3.38 and a smaller range from 1.42 mV to 7.37 mV. The input neurons exhibit a much higher standard deviation (20.38 mV) compared to the PANI–proteinoid sample (0.72 mV), suggesting a bigger range of spike amplitudes in the input neurons.

Figure 20 depicts the phasic spiking patterns exhibited by the input neurons and the associated response of the PANI–proteinoid sample. The input neurons exhibit discrete phases of spiking activity, characterised by clusters of spikes succeeded

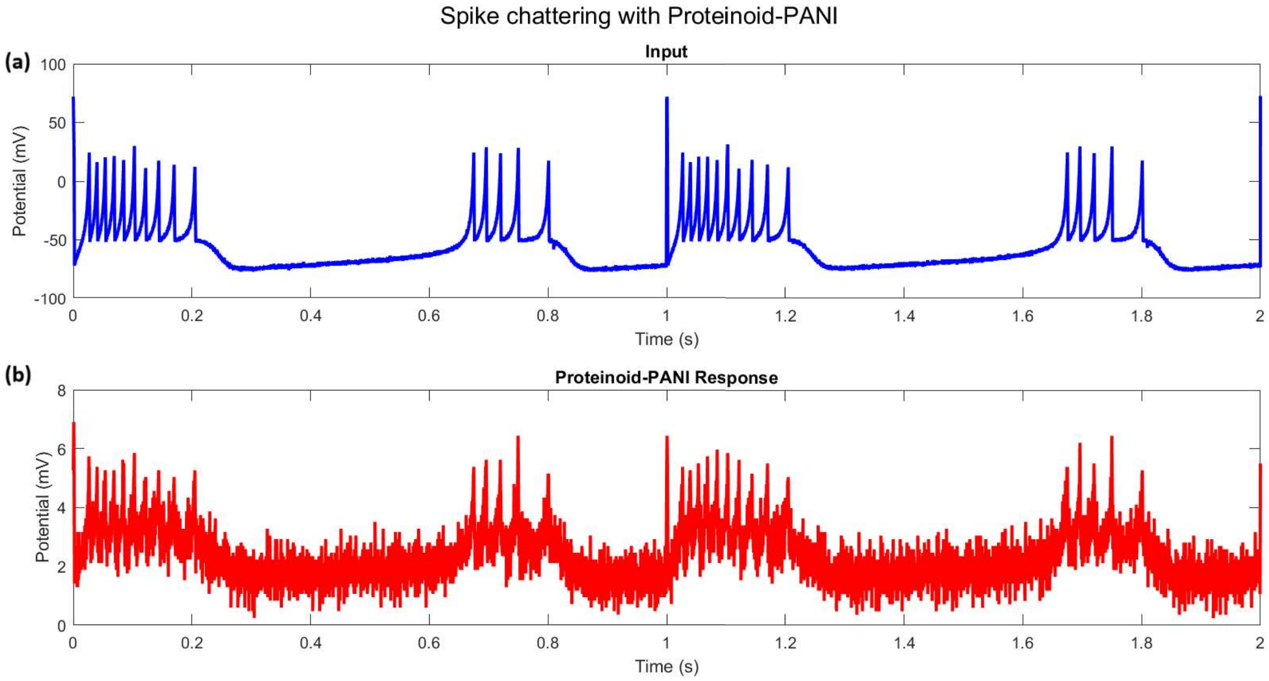

Figure 19. Comparative analysis of chattering spikes observed in input neurons and PANI–proteinoid samples. (a) The input neurons produce chattering spikes, which are characterised by fast and unpredictable patterns of spiking with different amplitudes. (b) The PANI–proteinoid samples exhibit a more regular and consistent spiking behaviour with lower chattering in response to the chattering spikes.

by intervals of inactivity. In contrast, the PANI–proteinoid sample demonstrates a more uniform and consistent spiking pattern, characterised by decreased phasic behaviour and a narrower range of spike amplitudes.

The differences in the phasic spiking activity and statistical characteristics between the input neurons and the PANI–proteinoid sample indicate that the PANI–proteinoid system exerts a regulatory influence on the phasic spiking dynamics. The PANI–proteinoid sample has a more uniform and consistent spiking pattern, showing lower phasic behaviour and less variation in spike amplitudes compared to the input neurons. The regulatory impact can be attributed to the unique features of the PANI–proteinoid system, including its conductivity and charge transport processes, which might contribute to the stabilisation and smoothing of the phasic spiking patterns.

Induced Spiking

The induced spiking behavior of the PANI–proteinoid samples was investigated and compared to the input neurons. The statistical comparison between the input neurons and the PANI–proteinoid samples for induced spiking is presented in Table 9. The kurtosis and skewness metrics offer information about the distribution of the spike amplitudes. The input neurons display a higher skewness value of 3.91 mV, but the PANI–proteinoid samples had a skewness value of 2.85 mV. This suggests that the amplitudes of the spikes from the input neurons are distributed in a way that is not symmetrical, with a greater number of spikes having higher values. Comparatively, the kurtosis value of 21.85 mV for the input neurons is considerably more than the kurtosis of 13.32 mV for the PANI–proteinoid samples. This indicates that the input neurons have a distribution that is more concentrated at its peak and has more extreme values in the tails. The sample statistics provide more evidence of the differences between the input neurons and the PANI–proteinoid sample. The mean value of the input neurons is -62.02 mV, with a large range from -73.10 to 72.55. On the other hand, the PANI–proteinoid samples demonstrate a greater average value of 2.12 mV and a smaller range spanning from 0.24 mV to 6.90 mV. The standard deviation of the input neurons (14.27 mV) exceeds that of the PANI–proteinoid samples (0.56 mV), suggesting a higher level of variability in the spike amplitudes of the input neurons.

The results indicate that the PANI–proteinoid system has a stabilising impact on induced spiking behaviour. The PANI–proteinoid samples display a more uniform and consistent pattern of spikes, with decreased variability in the amplitudes of the spikes when compared to the input neurons. The stabilising effect seen can be ascribed to the distinctive characteristics of the PANI–proteinoid system, including its conductivity and charge transport processes. These attributes likely contribute to the regulation and stabilisation of the induced spiking activity. The capacity of PANI–proteinoid to stabilise induced spiking

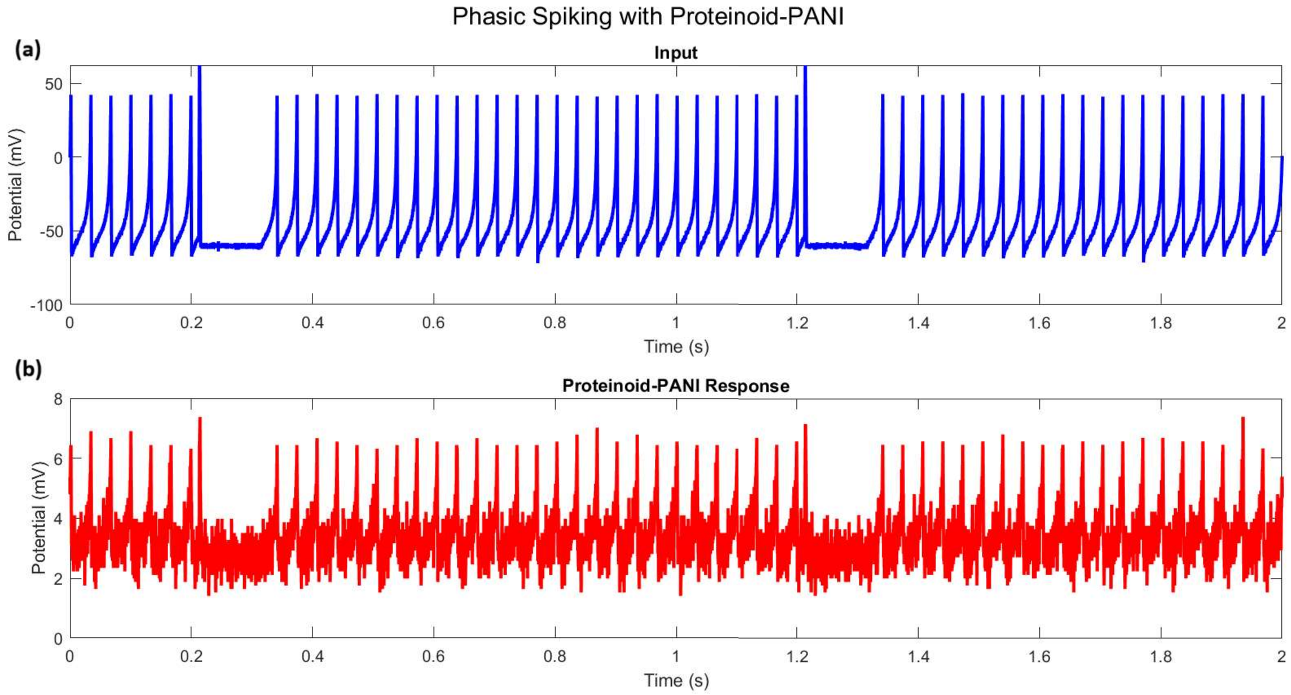

Figure 20. Comparison of phasic spiking patterns between input neurons and PANI–proteinoid sample. (a) Phasic spiking of input neurons, showing distinct phases of spiking activity followed by quiescent periods. (b) Response of PANI–proteinoid sample to the phasic spiking input, exhibiting a more regular and consistent spiking pattern with reduced phasic behavior.

behaviour underscores its potential for applications in neuromorphic computing and signal processing. PANI–proteinoid has the potential to enhance the dependability and resilience of artificial neural networks and bio–inspired computing systems by reducing the impact of irregular and burst–like spiking activity.

Spike Integrator

The spike integration characteristics of the proteinoid–PANI samples were investigated and compared to the input spike integrator. Table 9 presents the statistical comparison between the input spike integrator and the proteinoid–PANI samples. The kurtosis and skewness values offer valuable information regarding the dispersion of the integrated spike amplitudes. In contrast to the proteinoid–PANI samples, the input spike integrator demonstrates a maximal skewness of 5.51 mV (versus 3.20 mV for the latter). This suggests that the input spike integrator produces amplitudes of integrated spikes that are more asymmetrically distributed, exhibiting an extended tail as one approaches higher values. In the same way, the input spike integrator exhibits a considerably higher kurtosis value of 53.20 mV in comparison to the proteinoid–PANI samples' kurtosis of 22.50 mV. This difference implies that the input spike integrator possesses a distribution characterised by heavier tails and greater peak height.

In addition, the sample statistics underscore the distinctions between the proteinoid–PANI samples and the input spike integrator. The input spike integrator exhibits a broad range of values, from –89.15 to 73.13 mV, with a mean value of −79.90 mV. On the other hand, the proteinoid–PANI samples demonstrate a greater average value of 0.34 mV and a more limited range spanning from –1.39 to 5.50 mV. The larger standard deviation (11.29 mV) of the input spike integrator compared to the proteinoid–PANI samples (0.45 mV), suggests that the integrated spike amplitudes of the input spike integrator are more variable.

Tonic Spiking

The tonic spiking behaviour of the proteinoid–PANI samples was investigated and compared to the input neurons. Table **??** presents the statistical comparison between the input neurons and the proteinoid–PANI samples for tonic spiking.

The skewness and kurtosis values provide insights into the distribution of the spike amplitudes. The input neurons exhibit a higher skewness of 4.40 mV compared to the proteinoid–PANI samples, which have a skewness of 3.02 mV. This indicates that the spike amplitudes of the input neurons are more asymmetrically distributed, with a longer tail towards higher values. Similarly, the kurtosis value of 33.84 mV for the input neurons is significantly higher than the kurtosis of 19.01 mV for the proteinoid–PANI samples, suggesting that the input neurons have a more peaked distribution with heavier tails.

Table 8. Statistical comparison of phasic spiking behavior between input neurons and proteinoid–PANI samples. The input neurons had a more asymmetric and peaked spike amplitude distribution than the proteinoid-PANI samples (skewness: 1.84 mV, kurtosis: 6.67 mV). The input neurons exhibit a lower mean spike amplitude (–45.83 mV) and a larger range (–71.94 mV to 62.23 mV) than proteinoid–PANI samples (mean: 3.38 mV, range: 1.42 mV to 7.37 mV). The spike amplitudes of input neurons are more variable than those of proteinoid–PANI samples (0.72 mV), since their standard deviation (20.38 mV) is much higher. These metrics indicate that proteinoid–PANI samples have a more stable and consistent phasic spiking behaviour than input neurons, with a more symmetric distribution, shorter range, and reduced spike amplitude variability.

Statistics	Input	Proteinoid–PANI
Skewness	2.05	1.84
Kurtosis	7.55	6.67

Sample Statistics	Mean	Max	Min	Std
Input	−45.83	62.23	−71.94	20.38
Proteinoid-PANI	3.38	7.37	1.42	0.72

Table 9. Statistical comparison of induced spiking behaviour between input neurons and proteinoid–PANI samples. The input neurons had a more asymmetric and heavy–tailed spike amplitude distribution than the proteinoid–PANI samples (skewness: 2.85 mV, kurtosis: 13.32 mV). Sample statistics show that input neurons had a lower mean spike amplitude (–62.02) and a broader range (–73.10 to 72.55) than proteinoid–PANI samples (mean: 2.12 mV, range: 0.24 mV to 6.90 mV). Due to their larger spike amplitude variability, input neurons have a higher standard deviation (14.27 mV) than proteinoid–PANI samples (0.56 mV). Compared to input neurons, proteinoid–PANI samples have a more stable and consistent induced spiking behaviour, with a more symmetric distribution, shorter range, and reduced spike amplitude variability.

Statistics	Input	Proteinoid-PANI
Skewness	3.91	2.85
Kurtosis	21.85	13.32

Sample Statistics	Mean	Max	Min	Std
Input	−62.02	72.55	−73.10	14.27
Proteinoid–PANI	2.12	6.90	0.24	0.56

The sample statistics further highlight the differences between the input neurons and the proteinoid–PANI samples. The input neurons have a lower mean value of –59.74 mV, with a wide range spanning from –72.52 mV to 72.00 mV. In contrast, the proteinoid–PANI samples exhibit a higher mean value of 2.79 mV and a narrower range from 1.06 mV to 7.02 mV. The standard deviation of the input neurons (11.48 mV) is also larger than that of the proteinoid–PANI samples (0.45 mV), indicating greater variability in the spike amplitudes of the input neurons.

Mixed Mode Spiking

The mixed mode spiking behavior of the proteinoid–PANI samples was compared to that of the input neurons. Figure 24 depicts the differences in the spiking patterns and distributions of spike amplitudes between the two systems. The input neurons (Figure 24a) display a mixed mode spiking pattern, which is characterised by a combination of tonic spiking and bursting. The spike amplitudes of the input neurons exhibit a highly skewed distribution, with a skewness score of 4.06, indicating a longer tail towards higher values. The diverse spectrum of spike amplitudes (average: –61.52 mV, range: –74.23 mV to 70.84 mV) and significant level of variability (standard deviation: 9.59 mV) indicate a more intricate and less uniform mixed mode spiking pattern in the input neurons.

On the other hand, the proteinoid–PANI samples (Figure 24b) exhibit a distribution of spike amplitudes that is more balanced and evenly distributed (skewness: 2.10). The range of spike amplitudes is narrower, with an average of 2.51 mV and a range of 0.60 mV to 6.44 mV. Additionally, there is less variation in spike amplitudes, with a standard deviation of 0.36 mV, when compared to the input neurons. These findings imply that the proteinoid–PANI samples have a more stable and consistent mixed mode spiking behaviour, indicating their potential to represent complex information patterns in neuromorphic systems.

Table 11 provides a comprehensive statistical analysis of the spiking characteristics in mixed mode between the input neurons and the proteinoid–PANI samples. The skewness and kurtosis measurements indicate that the spike amplitudes of the input neurons have a distribution that is more asymmetric (skewness: 4.06) and has a higher peak with heavier tails (kurtosis: 28.07) compared to the proteinoid–PANI samples (skewness: 2.10, kurtosis: 12.77).

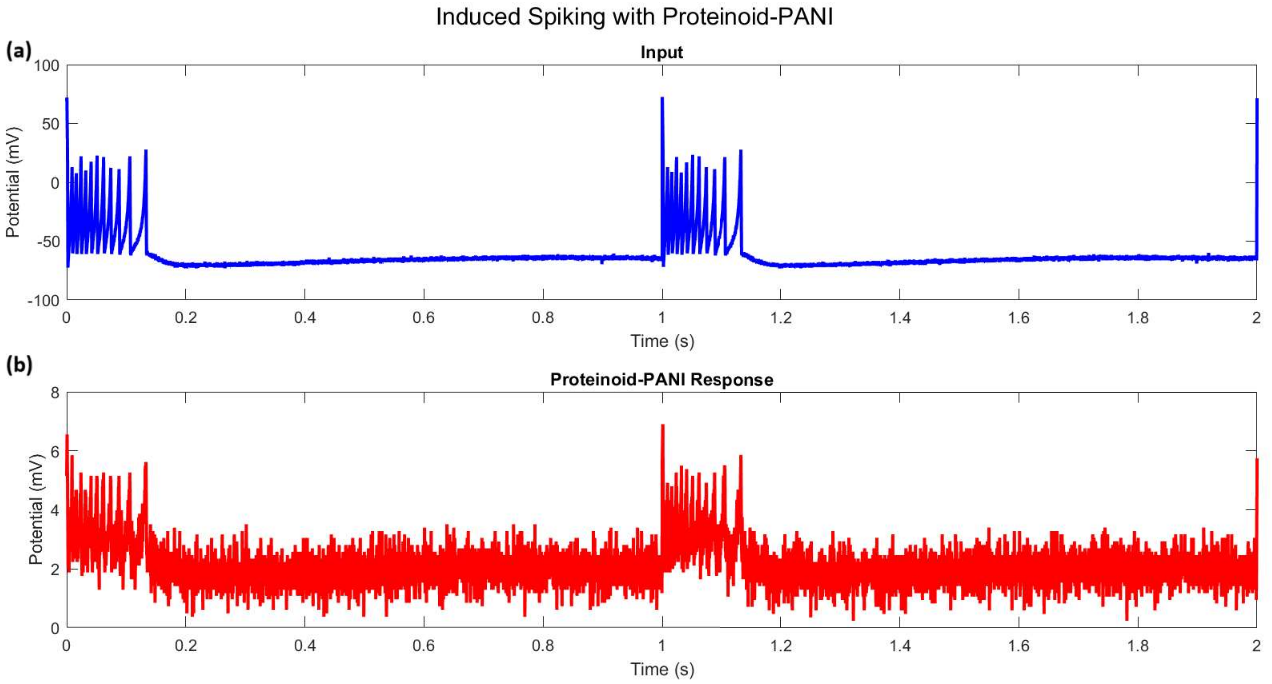

Figure 21. Comparison of induced spiking patterns between input neurons and PANI–proteinoid samples. (a) Induced spiking of input neurons, showing highly irregular and burst–like spiking activity with large variations in spike amplitudes (skewness: 3.91 mV, kurtosis: 21.85 mV). (b) Response of PANI–proteinoid samples to the induced spiking input, exhibiting a more regular and consistent spiking pattern with reduced variability in spike amplitudes (skewness: 2.85 mV, kurtosis: 13.32 mV). The PANI–proteinoid samples demonstrate a stabilizing effect on the induced spiking behavior.

The sample data provide additional emphasis on the differences between the two systems. The mean spike amplitude of the input neurons is –61.52 mV, which is lower than that of the proteinoid–PANI samples (mean: 2.51 mV). The range of spike amplitudes for the input neurons is greater, ranging from –74.23 mV to 70.84 mV, compared to the proteinoid–PANI samples which range from 0.60 mV to 6.44 mV. In addition, the standard deviation of the input neurons (9.59 mV) is significantly larger than that of the proteinoid–PANI samples (0.36 mV), suggesting a higher level of variability in the spike amplitudes of the input neurons.

The statistical metrics indicate that the proteinoid–PANI samples display a more steady and consistent mixed mode spiking behaviour in comparison to the input neurons. The proteinoid–PANI samples exhibit a more balanced distribution of spike amplitudes, a shorter range, and less variability, indicating their improved capacity to produce consistent and predictable mixed mode spiking patterns.

The results depicted in Figure 24 and Table 11 offer substantial information regarding the variations in the mixed mode spiking behaviour between the input neurons and the proteinoid–PANI samples.

FT–IR Analysis of Polyaniline Synthesized with Different Oxidants: Ferrous Chloride, Ferrous Nitrate, and Ammonium Persulfate

The FT–IR spectra of PANI–$FeCl_3$ (Table 13) display a broad variety of peaks spanning from 410.49 cm^{-1} to 3854.19 cm^{-1}, with intensities ranging from 0.263 to 0.306. The occurrence of peaks in the lower wavenumber range (400–900 cm^{-1}) can be attributed to the out–of–plane bending vibrations of C–H in the substituted benzenoid rings[62]. The peaks observed at wavelengths between 1200–1600 cm^{-1} are indicative of the stretching vibrations of C–N and C=N bonds inside the quinoid and benzenoid rings[63]. The region of higher wavenumbers (3000–3900 cm^{-1}) exhibits peaks associated with the stretching vibrations of N–H and O–H groups[64].

Conversely, the FT–IR spectra of PANI–$FeNO_3$ (Table 14) display a comparatively lower quantity of peaks in comparison to PANI—$FeCl_3$. The peaks are detected within the spectral range of 402.60 cm^{-1} to 2970.00 cm^{-1}, exhibiting intensities ranging from 0.0968 to 0.178. The peaks in the lower wavenumber band (400–600 cm^{-1}) have similarities to those seen in PANI–$FeCl_3$, suggesting the existence of C–H out–of–plane bending vibrations. Nevertheless, the magnitudes of these peaks are much diminished in PANI–$FeNO_3$. The peaks in the range of 1200–1600 cm^{-1}, which correspond to the stretching vibrations of C–N

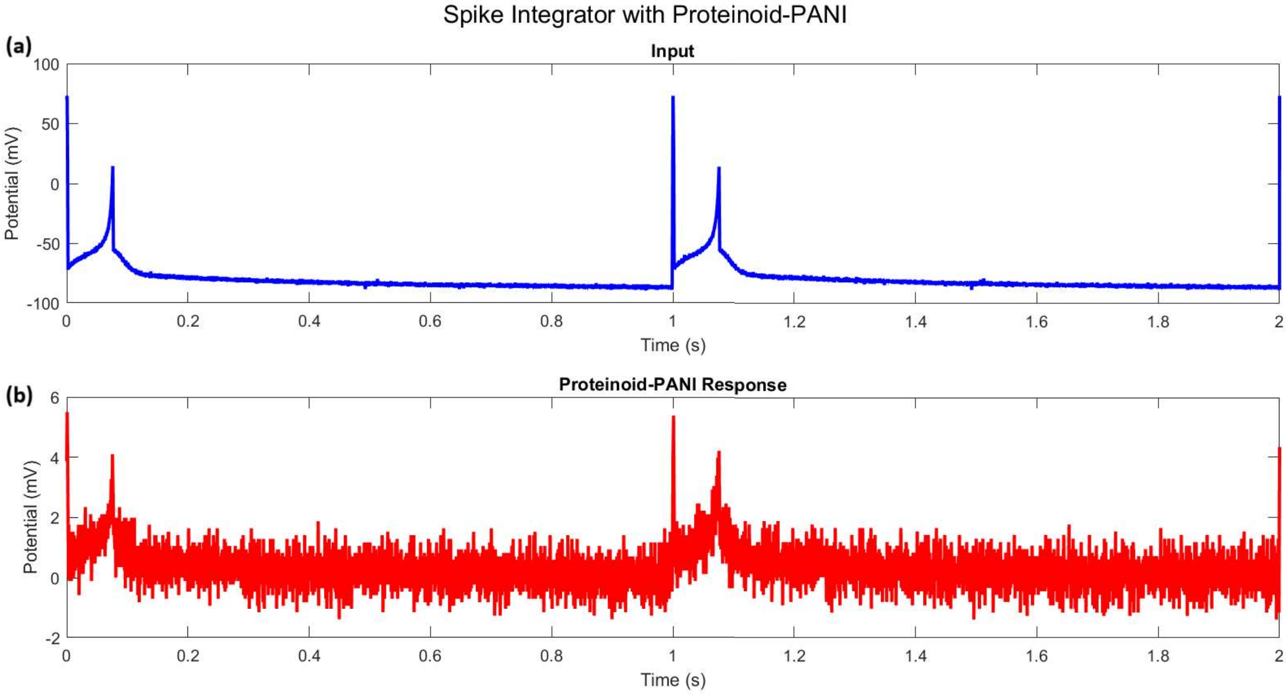

Figure 22. An analysis of spike integration between proteinoid–PANI samples and the input spike integrator. (a) Input spike integrator: The plot depicts the integrated spike amplitudes with time, demonstrating a strongly skewed distribution (skewness: 5.51 mV) with a long tail towards higher values. The input spike integrator has a wide range of integrated amplitudes (mean: −79.90 mV, range: −89.15 to 73.13 mV) and a significant level of variability (standard deviation: 11.29 mV), indicating a more intense and inconsistent integration process. (b) Proteinoid–PANI samples: The plot shows a more symmetric distribution of integrated spike amplitudes (skewness: 3.20 mV), a shorter range (mean: 0.34 mV, range: –1.39 mV to 5.50 mV), and lower variability (std: 0.45 mV) than the input spike integrator. The proteinoid–PANI samples show more steady and consistent spike integration behaviour, indicating their potential for robust information processing in neuromorphic systems.

and C=N bonds, are also observed in the sample. However, their intensities are lower compared to PANI–$FeCl_3$.

The Fourier Transform Infrared (FT–IR) spectra of PANI–APS (Table 15) exhibit peaks ranging from 409.32 cm^{-1} to 2970.03 cm^{-1}, with intensities ranging from 0.252 to 0.361. The positions and intensities of the peaks in the lower wavenumber range (400–900 cm-1) resemble those observed in PANI—$FeCl_3$, indicating the presence of C–H out–of–plane bending vibrations. The peaks in the range of 1200–1600 cm^{-1}, which correspond to the stretching vibrations of C–N and C=N bonds, are also evident in PANI–APS. The intensities of these peaks are similar to or greater than those observed in PANI–$FeCl_3$[65].

Upon comparing the three samples, it is evident that PANI–$FeCl_3$ demonstrates the greatest number of peaks and substantially higher intensities in comparison to PANI–$FeNO_3$ and PANI–APS. Using $FeCl_3$ as an oxidising agent leads to a polyaniline structure that is more organised and has a higher degree of conjugation[66]. The decreased number of peaks and diminished intensities in PANI–$FeNO_3$ suggest a less organised structure and lower level of conjugation in comparison to PANI–$FeCl_3$[65]. However, PANI–APS has similar peak locations and intensities to PANI—$FeCl_3$, indicating a comparable level of conjugation and structural order[65].

The variations in the FT–IR spectra of the three polyaniline samples can be attributed to the characteristics of the oxidising agents employed in the synthesis procedure. The oxidising agent is essential in the process of polymerizing aniline and determining the final structure and characteristics of polyaniline[67].$FeCl_3$ is recognised as a potent oxidising agent that facilitates the development of a highly conjugated and organised structure of polyaniline[68]. On the contrary, $FeNO_3$ can result in a less organised arrangement and reduced level of conjugation because of its different oxidising power and reaction mechanism[69]. Ammonium persulfate (APS) is a frequently employed oxidising agent that produces polyaniline with a structure and conjugation similar to what can be produced with PANI–$FeCl_3$[70].

Firing Rate Curves for PANI–Proteinoid on Kombucha, PEN and ITO–glass Substrates

Table 10. Statistical comparison of spike integration behaviour between input spike integrator and proteinoid-PANI samples. The input spike integrator exhibits a significantly higher skewness (5.51) and kurtosis (53.20) compared to the proteinoid-PANI samples (skewness: 3.20, kurtosis: 22.50), indicating a more asymmetric and heavily-tailed distribution of integrated spike amplitudes. The sample statistics reveal stark differences, with the input spike integrator having a lower mean (-79.90) and a much wider range (-89.15 to 73.13) compared to the proteinoid-PANI samples (mean: 0.34, range: -1.39 to 5.50). Furthermore, the standard deviation of the input spike integrator (11.29) is substantially higher than that of the proteinoid-PANI samples (0.45), highlighting the greater variability in the integrated spike amplitudes. These metrics demonstrate that the proteinoid-PANI samples exhibit a more stable and consistent spike integration behavior, with a more symmetric distribution, narrower range, and significantly lower variability in integrated spike amplitudes compared to the input spike integrator.

Statistics	Input	Proteinoid–PANI
Skewness	5.51	3.20
Kurtosis	53.20	22.50

Sample Statistics	Mean	Max	Min	Std
Input	−79.90	73.13	−89.15	11.29
Proteinoid–PANI	0.34	5.50	−1.39	0.45

Table 11. Statistical analysis of input neuron and proteinoid-PANI tonic spiking. Compared to proteinoid–PANI samples (skewness: 3.02 mV, kurtosis: 19.01 mV), input neurons had a more asymmetric and peaked spike amplitude distribution (4.40 mV, 33.84 mV). The input neurons exhibit a lower mean spike amplitude (–59.74 mV) and a broader range (–72.52 mV to 72.00 mV) than proteinoid–PANI samples (mean: 2.79 mV, range: 1.06 mV to 7.02 mV). The input neurons' standard deviation (11.48 mV) is much higher than that of the proteinoid–PANI samples (0.45 mV), suggesting increased spike amplitude variability. These measures show that proteinoid–PANI samples had a more stable and consistent tonic spiking behaviour than input neurons, with a more symmetric distribution, shorter range, and reduced spike amplitude variability.

Statistics	Input	Proteinoid-PANI
Skewness	4.40	3.02
Kurtosis	33.84	19.01

Sample Statistics	Mean	Max	Min	Std
Input	-59.74	72.00	-72.52	11.48
Proteinoid-PANI	2.79	7.02	1.06	0.45

Table 12. Statistical analysis of proteinoid–PANI and input neuron mixed mode spiking. The input neurons' spike amplitudes are more asymmetric (skewness: 4.06 mV) and peaked with heavier tails (kurtosis: 28.07 mV) than proteinoid–PANI samples (skewness: 2.10 mV, 12.77 mV). The input neurons exhibit a lower mean spike amplitude (–61.52 mV) and a broader range (–74.23 to 70.84 mV) than proteinoid–PANI samples (mean: 2.51 mV, range: 0.60 to 6.44 mV). The input neurons' standard deviation (9.59 mV) is higher than the proteinoid–PANI samples' (0.36 mV), indicating more spike amplitude variability. Proteinoid–PANI samples have a more symmetric distribution, shorter range, and lower spike amplitude variability than input neurons, suggesting more stable and consistent mixed mode spiking.

Statistics	Input	Proteinoid–PANI
Skewness	4.06	2.10
Kurtosis	28.07	12.77

Sample Statistics	Mean	Max	Min	Std
Input	–61.52	70.84	–74.23	9.59
Proteinoid-PANI	2.51	6.44	0.60	0.36

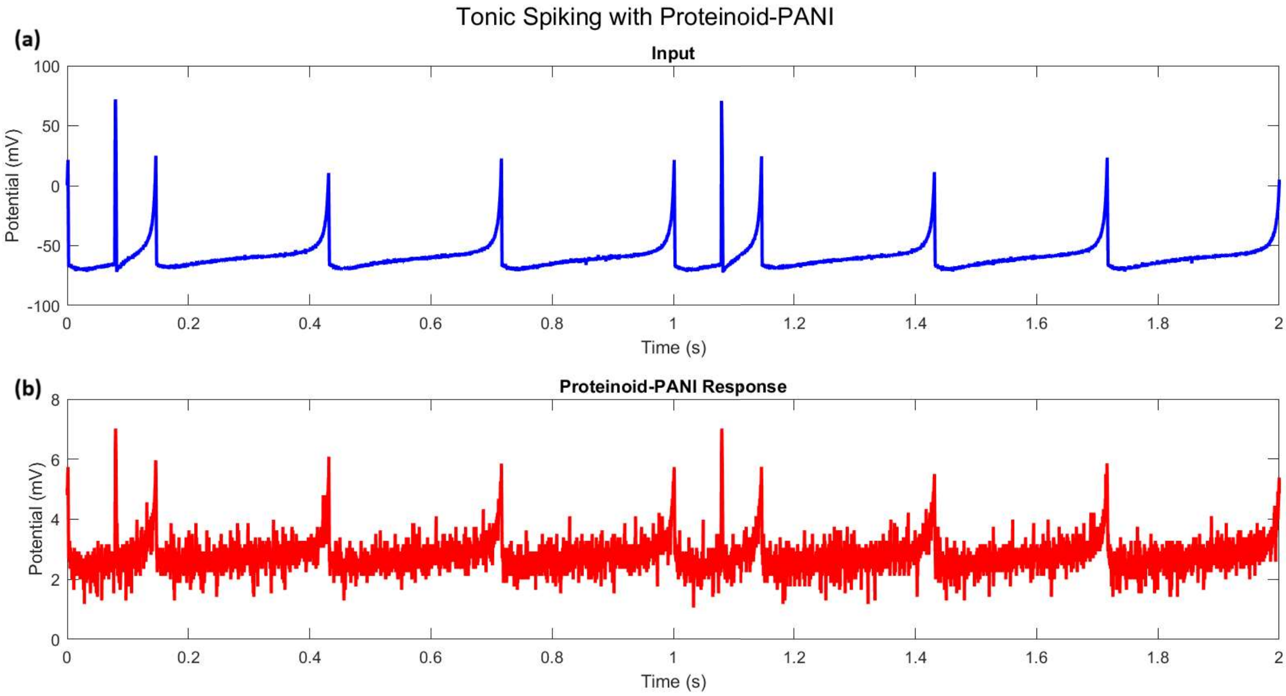

Figure 23. Comparison of tonic spiking behavior between input neurons and proteinoid–PANI samples. (a) Input neurons: The plot displays the tonic spiking activity, characterized by a highly skewed distribution of spike amplitudes (skewness: 4.40 mV) with a long tail towards higher values. The input neurons exhibit a wide range of spike amplitudes (mean: –59.74 mV, range: -72.52 mV to 72.00 mV) and a high level of variability (std: 11.48 mV), indicating a more irregular and less consistent tonic spiking pattern. (b) Proteinoid–PANI samples: The plot shows a more symmetric distribution of spike amplitudes (skewness: 3.02 mV) with a narrower range (mean: 2.79 mV, range: 1.06 mV to 7.02 mV) and lower variability (std: 0.45) compared to the input neurons. The proteinoid–PANI samples demonstrate a more stable and consistent tonic spiking behaviour, suggesting their potential for reliable information encoding in neuromorphic systems.

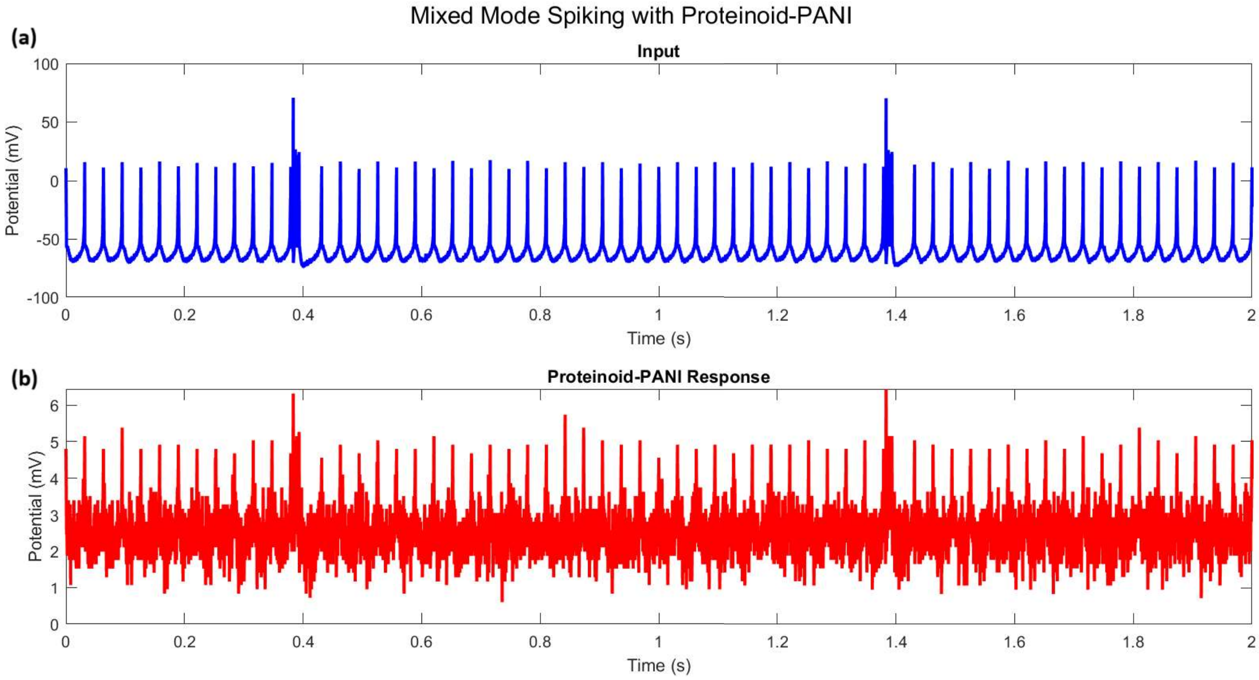

Figure 24. An analysis of the spiking behaviour in input neurons and proteinoid–PANI samples is conducted to compare their mixed mode characteristics. (a) Input neurons: The plot exhibits a mixed mode spiking activity, which is characterised by a combination of tonic spiking and bursts. The input neurons display a significantly skewed distribution of spike amplitudes, with a skewness score of 4.06 mV, indicating a lengthy tail towards higher values. The diverse spectrum of spike amplitudes (mean: –61.52 mV, range: –74.23 to 70.84 mV) and significant amount of variability (std: 9.59 mV) suggest a more intricate and less uniform mixed mode spiking pattern. (b) Proteinoid–PANI samples: The graph illustrates a distribution of spike amplitudes that is more balanced (skewness: 2.10 mV), with a shorter range (mean: 2.51 mV, range: 0.60 mV to 6.44 mV), and less variability (std: 0.36 mV) compared to the input neurons. The proteinoid–PANI samples exhibit a more enduring and uniform mixed mode spiking behaviour, indicating their potential to represent intricate information patterns in neuromorphic systems.

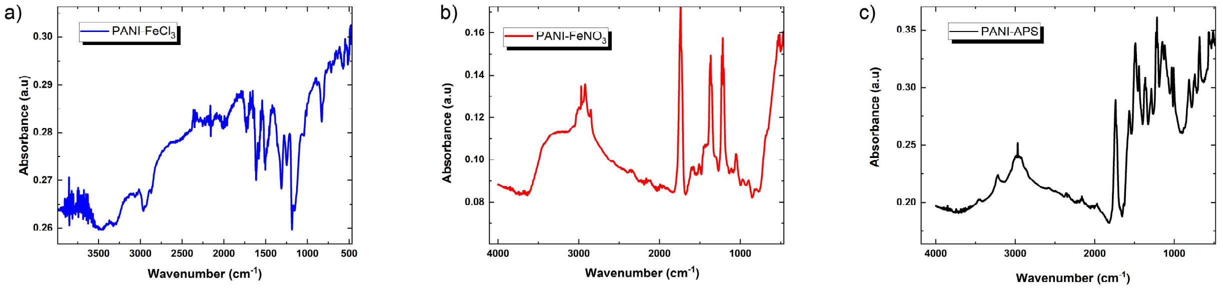

Figure 25. The FT–IR spectra of polyaniline synthesised employing different oxidising agents are as follows: (a) PANI–$FeCl_3$, (b) PANI–$FeNO_3$, and (c) PANI–APS (ammonium persulfate). The spectra display clear peaks that correspond to different functional groups and vibrational modes of polyaniline. The PANI–$FeCl_3$ compound exhibits a broad spectrum of peaks spanning from 410.49 cm^{-1} to 3854.19 cm^{-1}, which suggests the presence of a structurally well–organized and highly conjugated system. PANI–$FeNO_3$ has a reduced number of peaks with decreased intensities, indicating a less organised structure and lower level of conjugation in comparison to PANI–$FeCl_3$. PANI–APS has peak locations and intensities that closely resemble those of PANI–$FeCl_3$, suggesting a similar level of conjugation and structural organisation. The variations in the FT–IR spectra can be attributed to the characteristics of the oxidising agents employed in the synthesis procedure, which exhibit a substantial impact on the polymerization of aniline and subsequently affect the properties of polyaniline.

Table 13. FTIR Peaks of PANI–$FeCl_3$ Spectrum

Position	Intensity
410.49	0.303
425.78	0.293
446.95	0.306
455.23	0.302
465.30	0.301
478.61	0.302
510.43	0.297
548.22	0.300
615.12	0.298
729.65	0.295
895.83	0.291
1212.92	0.280
1279.08	0.279
1420.17	0.286
1457.93	0.281
1541.39	0.287
1559.93	0.284
1593.23	0.278
1637.02	0.285
1647.61	0.286
1654.21	0.289
1670.65	0.287
1685.18	0.288
1701.05	0.286
1718.53	0.283
1793.26	0.289
1993.07	0.284
2085.43	0.284
2161.65	0.286
2185.37	0.284
2323.27	0.284
2345.54	0.285
2362.54	0.285
3016.95	0.268
3568.18	0.263
3588.13	0.264
3619.86	0.265
3629.54	0.267
3650.24	0.267
3656.93	0.265
3670.24	0.266
3676.20	0.268
3690.15	0.267
3712.28	0.267
3736.35	0.268
3744.75	0.268
3751.97	0.268
3770.44	0.265
3801.91	0.267
3807.48	0.267
3821.70	0.268
3838.69	0.267
3854.19	0.271

Table 14. FTIR Peaks of PANI–Nitrate Spectrum

Position	Intensity
402.60	0.171
409.04	0.178
424.37	0.167
442.44	0.172
469.64	0.161
511.96	0.159
527.30	0.159
1052.28	0.103
1216.93	0.158
1228.87	0.149
1365.58	0.149
1510.42	0.0978
1581.13	0.0968
1738.53	0.173
2921.68	0.136
2970.00	0.135

Table 15. FTIR Peaks of Pani–APS Spectrum

Position	Intensity
409.32	0.339
420.63	0.331
510.74	0.349
565.95	0.348
681.41	0.344
740.87	0.312
813.71	0.307
1007.94	0.317
1031.97	0.317
1119.87	0.337
1150.09	0.340
1216.94	0.361
1228.42	0.348
1288.03	0.299
1365.38	0.309
1443.96	0.318
1488.90	0.339
1566.02	0.279
1738.29	0.289
2970.03	0.252

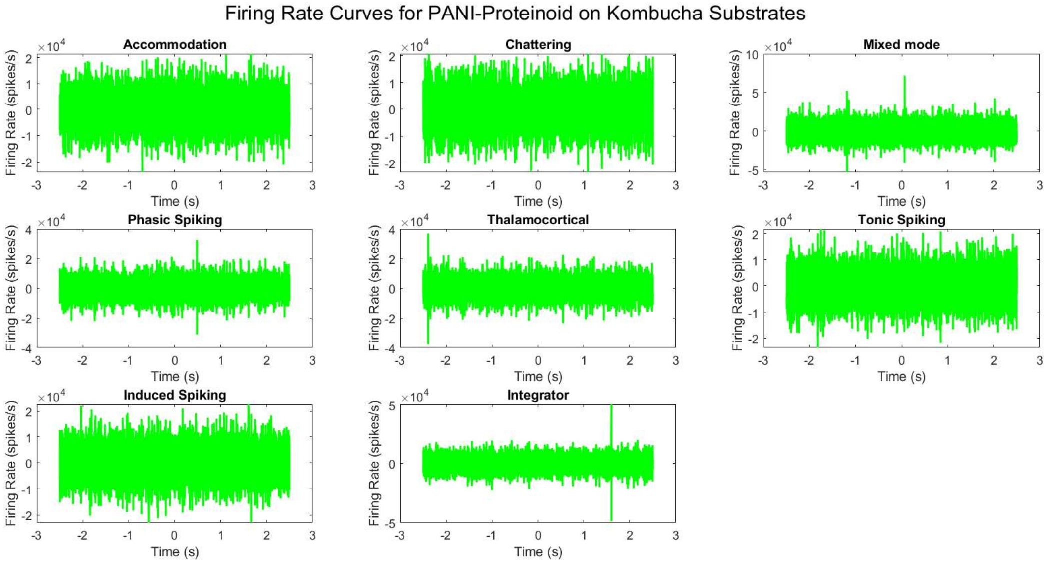

Figure 26. Graphs depicting the rate at which PANI-Proteinoid generates different patterns of spiking activity on the Kombucha substrate. Most of firing rates are negative, which suggests a suppression of spiking activity. The firing rate of mixed mode spiking is the lowest at –0.19 spikes/s, whereas induced spiking has a slightly positive firing rate of 0.02 spikes/s. The firing rate profile indicates that the Kombucha substrate has a suppressive effect on the spiking activity of PANI–Proteinoid.

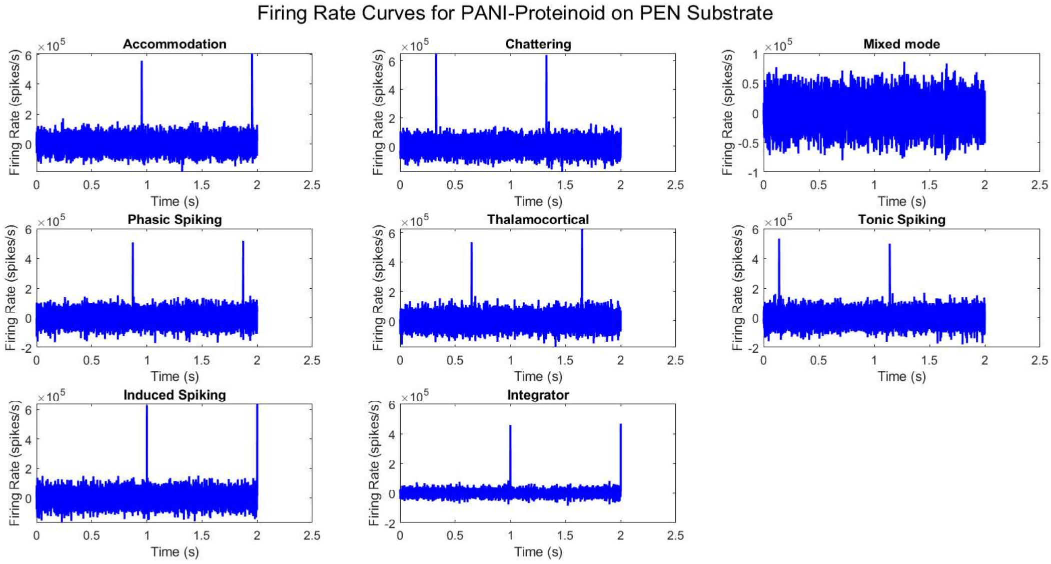

Figure 27. The firing rate graphs illustrate different spiking behaviours of PANI-Proteinoid on the PEN substrate. The firing rates display an equilibrium of positive and negative values, indicating a wide range of spiking activity. The firing rate of mixed mode spiking is substantially negative at –1.95 spikes/s, whereas phasic spiking and induced spiking have positive firing rates of 0.40 spikes/s and 0.32 spikes/s, respectively. The spiking behaviour of PANI–Proteinoid is influenced in a broader sense by the PEN substrate as compared to the Kombucha substrate.

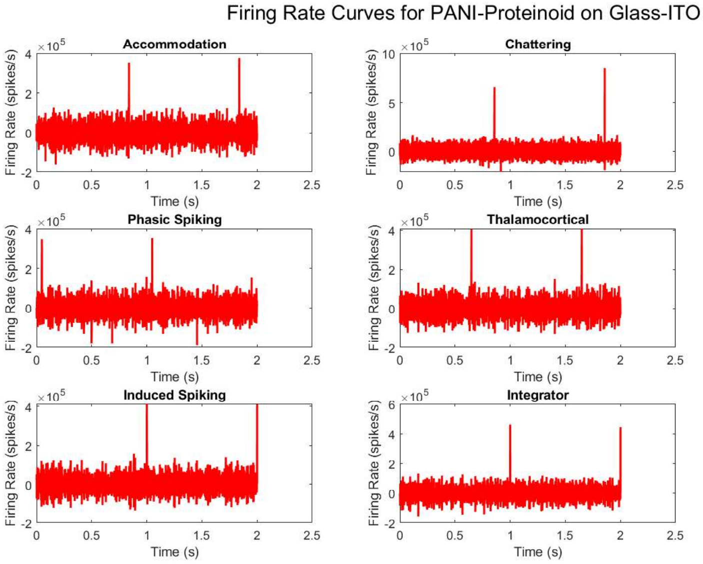

Figure 28. The firing rate graphs show all the different spiking behaviours of PANI-Proteinoid on the Glass-ITO substrate. The firing rates tend to be positive, indicating an increase in spiking activity. Tonic spiking has the maximum firing rate of 0.43 spikes per second, whereas chattering has a firing rate of –0.32 spikes per second. The ITO–glass substrate often increases the spiking activity of PANI–Proteinoid, although there may be some cases where this is not observed.

Table 16. Summary of Statistical Properties of Spike Behaviors

Behaviour	Statistics	Input (mean)	Input (std)	Proteinoid–PANI (mean)	Proteinoid–PANI (std)
Thalamocortical Stimulation	Skewness	4.43	11.47	3.01	0.45
	Kurtosis	34.19	11.47	18.83	0.45
Accommodation Spikes	Skewness	2.66	15.23	2.08	0.57
	Kurtosis	11.94	15.23	8.60	0.57
Chattering Spike Behavior	Skewness	1.72	19.85	1.24	0.78
	Kurtosis	6.90	19.85	4.60	0.78
Phasic Spiking Behavior	Skewness	2.05	20.38	1.84	0.72
	Kurtosis	7.55	20.38	6.67	0.72
Induced Spiking Behavior	Skewness	3.91	14.27	2.85	0.56
	Kurtosis	21.85	14.27	13.32	0.56
Spike Integration Behavior	Skewness	5.51	11.29	3.20	0.45
	Kurtosis	53.20	11.29	22.50	0.45
Tonic Spiking	Skewness	4.40	11.48	3.02	0.45
	Kurtosis	33.84	11.48	19.01	0.45
Mixed Mode Spiking	Skewness	4.06	9.59	2.10	0.36
	Kurtosis	28.07	9.59	12.77	0.36

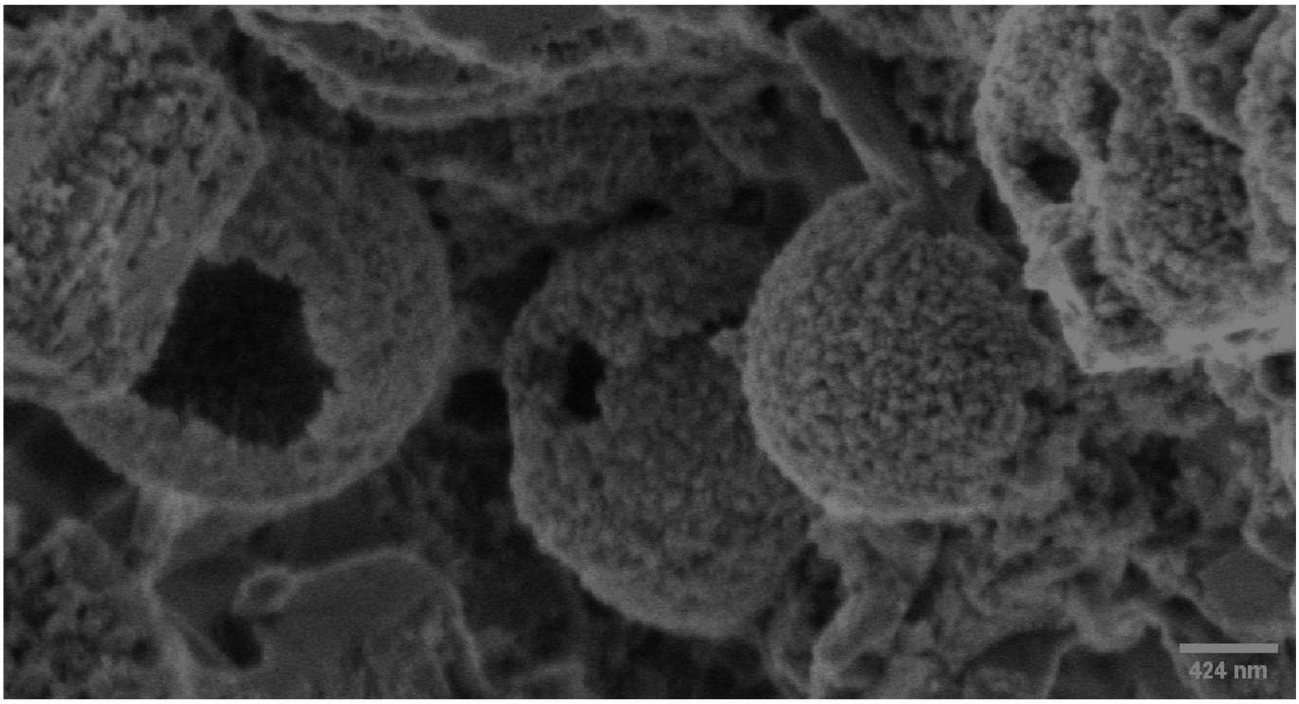

Figure 29. This picture is a Scanning Electron Microscopy (SEM) illustration of hollow proteinoid microspheres. These microspheres have the ability to act as carriers for polyaniline (PANI) nanospheres and are interconnected with PANI nanofibers. The proteinoid microspheres have dimensions ranging from 1 to 2 μm and have a distinct hollow structure, which is apparent from the collapsed and partially open microspheres. The empty space inside these microspheres is ideal for enclosing and protecting PANI nanospheres. The PANI nanospheres can be placed into the microspheres using several methods such in–situ polymerization or physical adsorption. The proteinoid microspheres are encircled with a network of PANI nanofibers, which smoothly blend with the structure of the microspheres. The nanofibers have diameters of around 50 nm and lengths that can reach several micrometres. They create a conductive and linked network, which improves charge transfer and increases the electrical properties of the composite material. The integration of hollow proteinoid microspheres and PANI nanofibers results in a unique hierarchical architecture that provides numerous advantages, including a large surface area, effective charge transfer, and regulated release of encapsulated PANI nanospheres. This hybrid material has significant promise for use in delivery of drugs, energy storage, and sensing. The integration of proteinoid microspheres with PANI nanostructures results in synergistic effects that can improve performance and functionality. The scale bar measures 424 nanometers.

Table 17. Cluster Centroids Per Dataset. The table shows K–means cluster centroids for each spiking behaviour dataset. Cluster centroids are (potential (mV), time (s)) tuples, representing each cluster's mean potential and temporal features. For each dataset, three cluster centroids show representative spiking patterns and their potential and time values. The cluster centroids summarise each dataset's spiking activity groups, allowing quantitative comparison across experimental settings. Different centroid values across datasets show the variety and selectivity of PANI–proteinoid responses to stimuli and spiking regimes. These centroids help characterise and evaluate the PANI–proteinoid system's dynamics by describing each dataset's spiking activity's typical potential and temporal characteristics.

Dataset	Cluster Centroid 1	Cluster Centroid 2	Cluster Centroid 3
Thalamocortical	(–54.5520, 2.9359)	(–8.6907, 4.5850)	(–64.9216, 2.5557)
Chattering	(–45.4117, 2.8735)	(–5.5061, 4.2265)	(–69.7813, 1.8415)
Phasic Spiking	(–31.4006, 3.8746)	(–55.9273, 3.0289)	(12.8904, 5.4028)
Induced Spiking	(–68.2052, 1.8892)	(–13.8883, 3.8894)	(–62.0676, 2.1319)
Integrator	(–57.9966, 1.1836)	(–82.9280, 0.2292)	(9.3325, 3.3933)
Tonic Spiking	(–63.0666, 2.6713)	(–2.0223, 4.8574)	(-47.4171, 3.2576)
Mixed Mode	(–56.3024, 2.7170)	(–17.8953, 3.8567)	(-66.0098, 2.3529)
Accommodation	(–54.6858, 2.5826)	(0.3099, 4.5224)	(–33.8702, 3.3281)

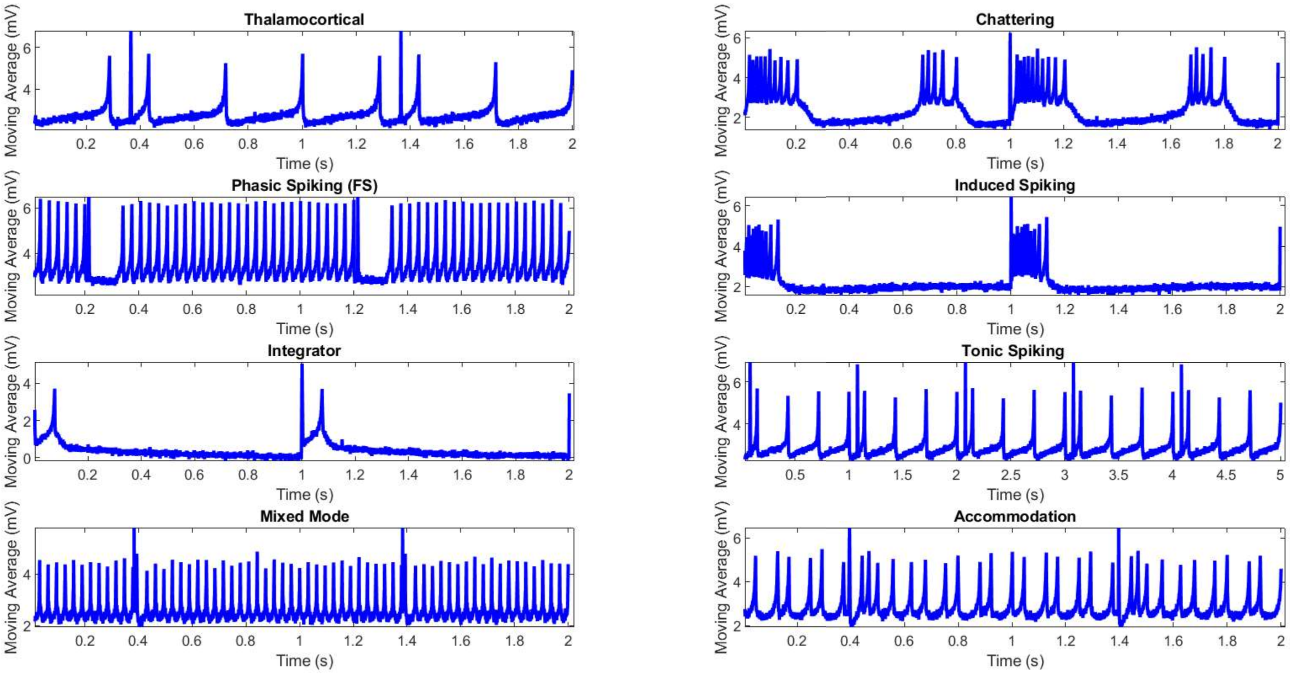

Figure 30. The figure illustrates the use of moving average subplots to analyse the trend of neural activity under different experimental conditions for diverse spiking patterns. Each subplot depicts the temporal moving average of spike amplitudes for a particular spiking pattern. A moving average window of 10 time steps was established to generate a smoothed depiction of the spike amplitudes, highlighting patterns and fluctuations in neural responses. The blue lines in each subplot depict the moving average of spike amplitudes. The x–axis indicates time in seconds, while the y–axis represents the corresponding moving average values.

Table 18. Proteinoid–PANI Spike Correlations. The table shows the Proteinoid–PANI system's membrane potential–time Pearson correlation coefficients for each spiking behaviour dataset. The correlation coefficients measure the membrane potential–time linear relationship's intensity and direction. A coefficient near to 1 shows a high positive correlation, implying membrane potential increases with time. All datasets had good correlation coefficients (0.8514–0.9630), suggesting that the Proteinoid–PANI membrane potential has a consistent and robust temporal dependence. As membrane potential and time are almost linear, the Chattering dataset has the greatest correlation (0.9630). The Mixed Mode dataset has the lowest correlation (0.8514) but an extensive positive association. These correlation coefficients reveal the Proteinoid–PANI system's temporal dynamics and membrane potential changes' predictability. High correlations across varied spiking behaviours reveal the system's ability to generate consistent and predictable electrical activity patterns, making it a promising neuromorphic computing substrate.

Dataset	Correlation Coefficient
Thalamocortical	0.9067
Chattering	0.9630
Phasic Spiking	0.9548
Induced Spiking	0.9335
Integrator	0.9065
Tonic Spiking	0.9074
Mixed Mode	0.8514
Accommodation	0.9378

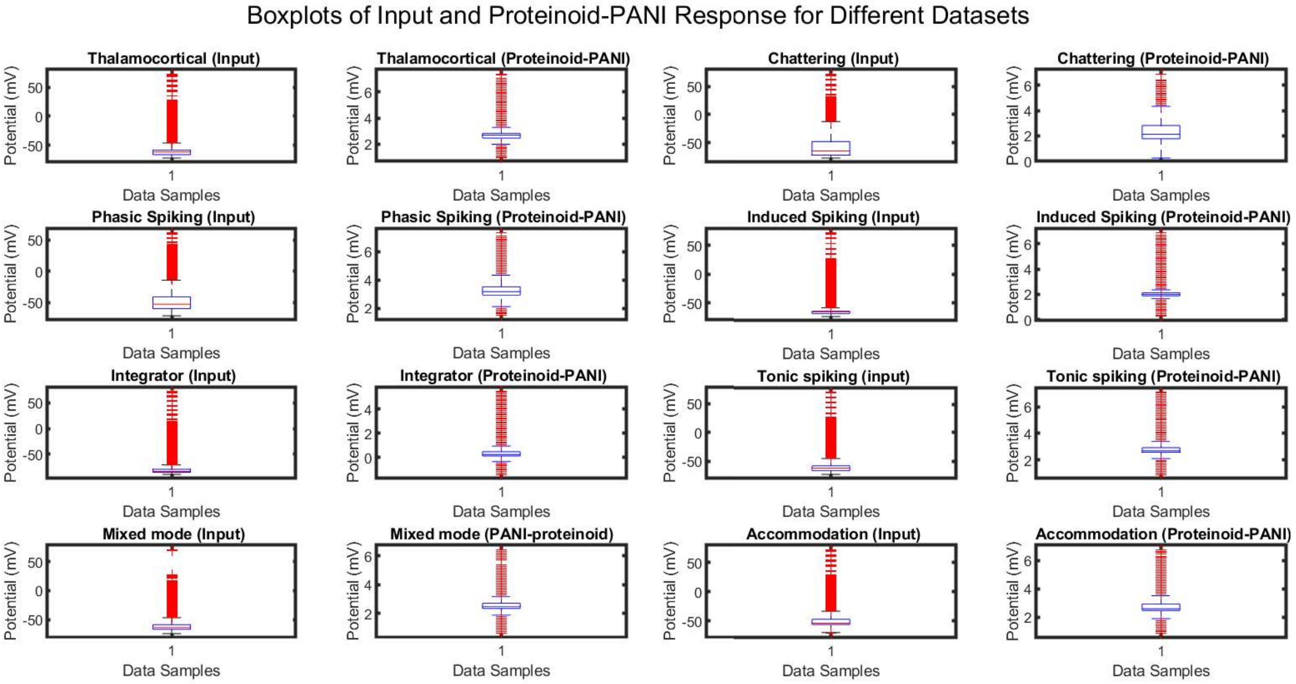

Figure 31. Box plot comparison of input neuron and PANI–proteinoid spike amplitudes for varied spiking behaviours. In all spiking types, input neurons have broader boxes and more outliers, indicating larger interquartile ranges (IQR) and more extreme voltage or spike amplitudes. PANI–proteinoid samples have narrower boxes and fewer outliers, indicating more concentrated and consistent voltage or spike amplitude ranges. The box plot measurements show that PANI–proteinoid samples had more stable and consistent spiking than input neurons. For thalamocortical stimulation, input neurons have a broader voltage dispersion (IQR: –67.83 mV to –52.97 mV, median: –60.92 mV) than PANI–proteinoid samples (IQR: 2.47 to 2.99, median: 2.73). Accommodation spikes show that input neurons have more potential variability than proteinoid–PANI samples. Phasic, mixed mode, tonic, and triggered spiking show that PANI–proteinoid samples can maintain a shorter range and more consistent spike amplitudes than input neurons. With a higher mean (–79.90 mV vs 0.34 mV), narrower range (–89.15 mV to 73.13 mV vs –1.39 mV to 5.50 mV), and lower standard deviation (11.29 mV vs 0.45 mV) than the input spike integrator, PANI–proteinoid samples are more stable. These results demonstrate the PANI–proteinoid system's ability to filter and regularise signals across spiking behaviours, changing highly varied inputs into more predictable output responses.

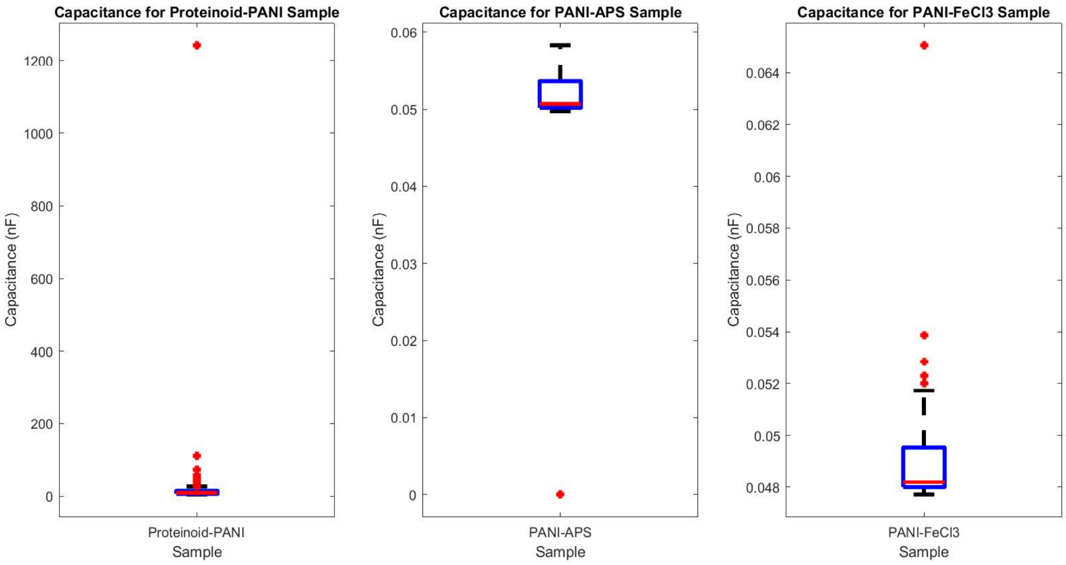

Figure 32. Box plot comparison of capacitance values for proteinoid–PANI, PANI–APS, and PANI–$FeCl_3$ samples.The proteinoid–PANI sample demonstrates a significantly higher capacitance in comparison to the other two samples, reaching a maximum value of 1242.00 nF at a frequency of 0.02 kHz and a lowest value of 4.52 nF at a frequency of 300 kHz. The average capacitance of the proteinoid–PANI sample is 16.23 nF, with a standard deviation of 71.66 nF, suggesting a significant variation in capacitance values. On the other hand, the PANI–APS and PANI–$FeCl_3$ samples exhibit significantly reduced capacitance values, averaging at 0.05 nF with standard deviation of 0.01 nF. The PANI–APS sample exhibits a peak capacitance of 0.06 nF at a frequency of 9.098 kHz and a minimum capacitance of 0.00 nF at a frequency of 0.1006 kHz. On the other hand, the PANI–$FeCl_3$ sample has a maximum capacitance of 0.07 nF at a frequency of 0.1225 kHz and a low capacitance of 0.05 nF at a frequency of 173.1 kHz. The results suggest that the proteinoid–PANI sample exhibits improved capacitive characteristics, indicating its potential for applications in energy storage and capacitive sensing.

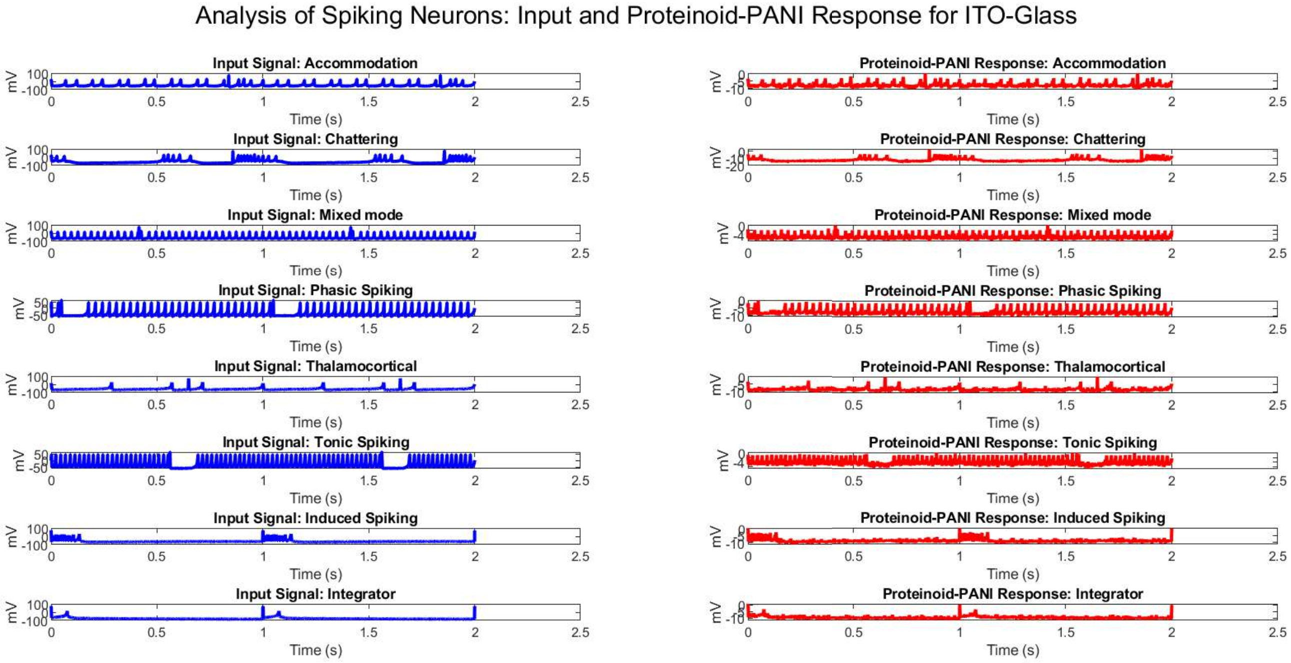

Figure 33. Spiking behaviour of PANI–Proteinoid on the ITO–glass substrate. The graphic displays the potential (mV) with time for various spiking behaviours, such as accommodation, chattering, mixed mode, phasic, thalamocortical, tonic, induced, and integrator spiking. The potential levels vary from –16.54 mV to 3.36 mV, with chattering spiking exhibiting the widest range (min: –16.54 mV, max: 3.36 mV). The integrator spiking behaviour has the lowest potential values among the spiking patterns, with a minimum of –11.73 mV. Spiking patterns on the ITO–glass substrate are more variable compared to those on the Kombucha substrate, with noticeable changes in potential ranges and waveforms among the different spiking behaviours.

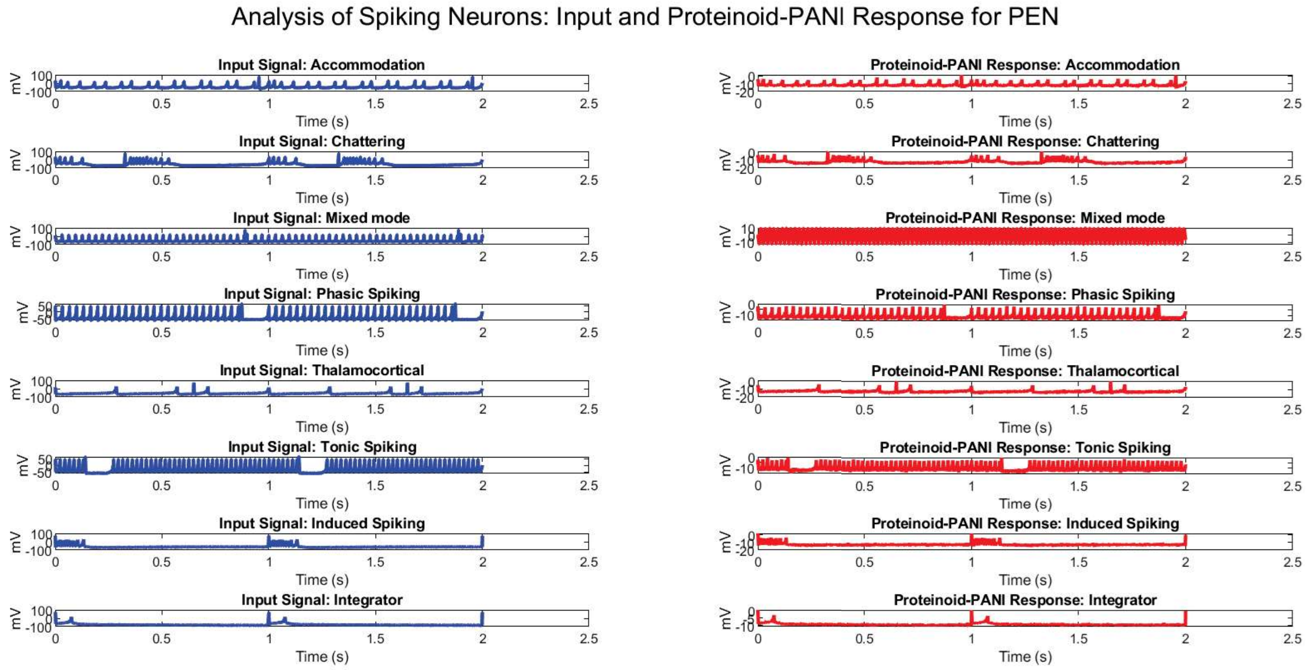

Figure 34. PANI-Proteinoid exhibits spiking behaviour on the PEN substrate. The graphic depicts the potential (mV) with time for various spiking behaviours, such as accommodation, chattering, mixed mode, phasic, thalamocortical, tonic, induced, and integrator spiking. The potential values range from –16.14 mV to 10.13 mV, with mixed mode spiking displaying the highest potential values (max: 10.13 mV) and chattering spiking showing the lowest potential values (min: –16.14 mV). Spiking patterns on the PEN substrate exhibit significant variety, with distinct potential ranges and waveforms for each spiking behaviour. The variation in spiking behaviour on the PEN substrate is similar to that observed on the ITO–glass substrate.

(62) Cochet, M.; et al. Theoretical and experimental vibrational study of polyaniline in base forms: non-planar analysis. part I. *J. Raman Spectrosc.* **2000**, 31, 1029–1039.
(63) Trchová, M.; Konyushenko, E.N.; Stejskal, J.; Kovářová, J.; Ćiric-Marjanovic, G. The conversion of polyaniline nanotubes to nitrogen-containing carbon nanotubes and their comparison with multi-walled carbon nanotubes. *Polym. Degrad. Stab.* **2009**, *94*, 929–938.
(64) Mu, S. Polyaniline with two types of functional groups: Preparation and characteristics. *Macromol. Chem. Phys.* **2005**, 206, 689–695.
(65) Molapo, K. M.; Ndangili, P. M.; Ajayi, R. F.; et al. Electronics of conjugated polymers (i): polyaniline. *Int. J. Electrochem. Sci.* **2012**, *7*, 11859–11875.
(66)Ho, K.-S.; Hsieh, T.; Kuo, C.; et al. Effect of aniline formaldehyde resin on the conjugation length and structure of doped polyaniline: spectral studies. *J. Polym. Sci.*, *Part A: Polym. Chem.* **2005**, *43*, 3116–3125.
(67) Zhang, L.; Zujovic, Z. D.; Peng, H.; et al. Structural characteristics of polyaniline nanotubes synthesized from different buffer solutions. *Macromolecules* **2008**, *41*, 8877–8884.
(68) Koosheh, H. B.; Modarresi-Alam, A. R. Solid-state synthesis of a new core–shell nanocomposite of polyaniline and silica via oxidation of aniline hydrochloride by fecl3. 6h2o. *Polym. for Adv. Technol.* **2016**, 27, 1038–1049.
(69) Zhang, Y.; Dou, C.; Li, L.; Wang, Y. A practical way to prepare thin polyaniline nanofibers with ferric nitrate as an oxidant. *Polym. Sci. Ser. A* **2014**, *56*, 146–151.
(70) David, S.; Nicolau, Y.; Melis, F.; Revillon, A.Molecular weight of polyaniline synthesized by oxidation of aniline with ammonium persulfate and with ferric chloride. *Synth. Met.* **1995**, *69*, 125 –126.
